# Systematic biases in DNA copy number originate from isolation procedures

**DOI:** 10.1186/gb-2013-14-4-r33

**Published:** 2013-04-24

**Authors:** Sebastiaan van Heesch, Michal Mokry, Veronika Boskova, Wade Junker, Rajdeep Mehon, Pim Toonen, Ewart de Bruijn, James D Shull, Timothy J Aitman, Edwin Cuppen, Victor Guryev

**Affiliations:** 1 Hubrecht Institute and University Medical Center Utrecht, Uppsalalaan 8, 3584 CT Utrecht, The Netherlands; 2 Current affiliation: Laboratory of Pediatric Gastroenterology, Wilhelmina Children's Hospital, University Medical Centre, Lundlaan 6, 3584 EA Utrecht, The Netherlands; 3 Department of Genetics, Cell Biology and Anatomy, 985805 University of Nebraska Medical Center, Omaha, Nebraska, 68198-5805, USA; 4 Medical Research Council Clinical Sciences Centre, Imperial College London, Hammersmith Hospital Campus, Du Cane Road, London, W12 0NN, UK; 5 Current affiliation: McArdle Laboratory for Cancer Research, Department of Oncology, University of Wisconsin, Madison, Wisconsin, 53706-1599, USA; 6 Department of Medical Genetics, UMC Utrecht, Universiteitsweg 100, 3584 GG Utrecht, The Netherlands; 7 Current affiliation: Laboratory of Genome Structure and Ageing; European Research Institute for the Biology of Ageing; RuG and UMC Groningen, Antonius Deusinglaan 1, 9713 AV, Groningen, The Netherlands

**Keywords:** Copy number variation, DNA isolation, Technological bias, Tissue specificity

## Abstract

**Background:**

The ability to accurately detect DNA copy number variation in both a sensitive and quantitative manner is important in many research areas. However, genome-wide DNA copy number analyses are complicated by variations in detection signal.

**Results:**

While GC content has been used to correct for this, here we show that coverage biases are tissue-specific and independent of the detection method as demonstrated by next-generation sequencing and array CGH. Moreover, we show that DNA isolation stringency affects the degree of equimolar coverage and that the observed biases coincide with chromatin characteristics like gene expression, genomic isochores, and replication timing.

**Conclusion:**

These results indicate that chromatin organization is a main determinant for differential DNA retrieval. These findings are highly relevant for germline and somatic DNA copy number variation analyses.

## Background

The ability to accurately detect DNA copy number variation (CNV) in both a sensitive and quantitative manner is important in many research areas. While the detection of CNVs previously relied on low resolution techniques like quantitative PCR or MLPA, high-resolution array-based comparative genomic hybridization (aCGH) and next-generation sequencing (NGS)-based depth of read coverage (DOC) approaches [1] now allow for detailed genome-wide analyses. However, both aCGH and DOC are complicated by the presence of 'wave patterns' in the raw data where the measurement deviates systematically from equimolar representation. These regions span up to tens of megabases and pose challenges on CNV calling. To reduce the number of false-positive calls introduced, algorithms were designed to suppress wave effects [2-6]. In these studies, quantity of DNA during hybridization, dye-biases, enzymatic effects, and correlations with GC content were proposed as the main contributors to the wave patterns. However, understanding the source of the observed patterns is important for reliable genome-wide analyses based on aCGH and NGS techniques.

## Results and discussion

**Figure 1:**
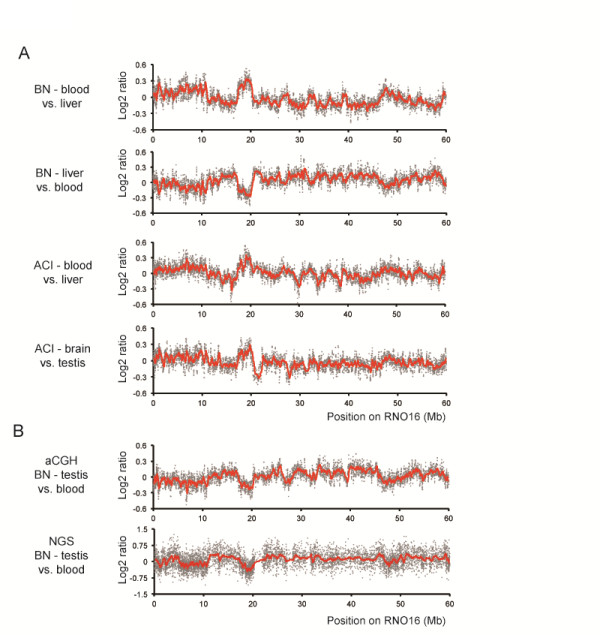
**Reproducible patterns in genome-wide aCGH and NGS data**. (**A**) Pairwise aCGH analysis results of blood, liver, brain, and testis samples for rat chromosome 16. For all panels, the variability in log2 ratios is displayed (each dot represents the median value over 100 consecutive probes). The top two panels show dye swap aCGH (Nimblegen) results using blood and liver samples from a single animal (Brown Norway strain). The third panel shows the comparison of blood and liver from an animal from a different inbred strain (ACI). The bottom panel shows the aCGH analysis results between brain and testis of that same ACI animal. (**B**) Comparison of aCGH hybridization signal with NGS depth of coverage analysis results. DNA isolated from the testis and from blood of the same animal was analyzed by aCGH (Nimblegen) and by low-pass next-generation sequencing (6.3-7.2 M reads; 0.075× - 0.086× genome coverage).

To discover the source of unequal DNA representation in genomic data we performed pairwise aCGH analyses comparing all possible combinations of DNA samples isolated from blood, brain, liver, and testis from two rats from different inbred strains. We observed large-scale tissue-specific variation in hybridization intensities that were reproducible between strains and consistent in dye-swap experiments (Figure 1A). Fold-changes for this variation could computationally be defined as tissue-specific CNVs (within the same strain) and were typically much lower than for germline CNVs (between strains). Even though the amplitude of variation did not exceed 30%, the reproducibility of tissue-specific differences between multiple rat strains was very high, both in terms of pattern and magnitude (Figure 1A). Theoretically, these patterns could reflect somatic copy number changes, in line with recently observed somatic heterogeneity [7-9]. Nevertheless, systematic artifacts of the methods used might also underlie such observations. In support of a potential systematic artifact we noted that the genomic regions involved are often megabases in size, while regular CNVs are typically much shorter. Although aCGH analyses using different platforms (Nimblegen and Agilent) and labeling techniques revealed highly similar patterns (not shown), shared artifacts associated with aCGH such as dye or sequence-dependent hybridization effects cannot be excluded. Therefore, we performed a depth of coverage analysis on four tissues from a single animal using NGS-based low-pass whole genome sequencing (5-10 M reads per sample) (Figure 1B). Interestingly, both aCGH and NGS show highly similar DNA content patterns (r^2 ^= 0.71, *P *< 0.001, Additional file 1), excluding previously proposed array-specific artifacts [2-5,10] as the sole basis for the observed patterns and suggesting a common source for the observed variation.

**Figure 2:**
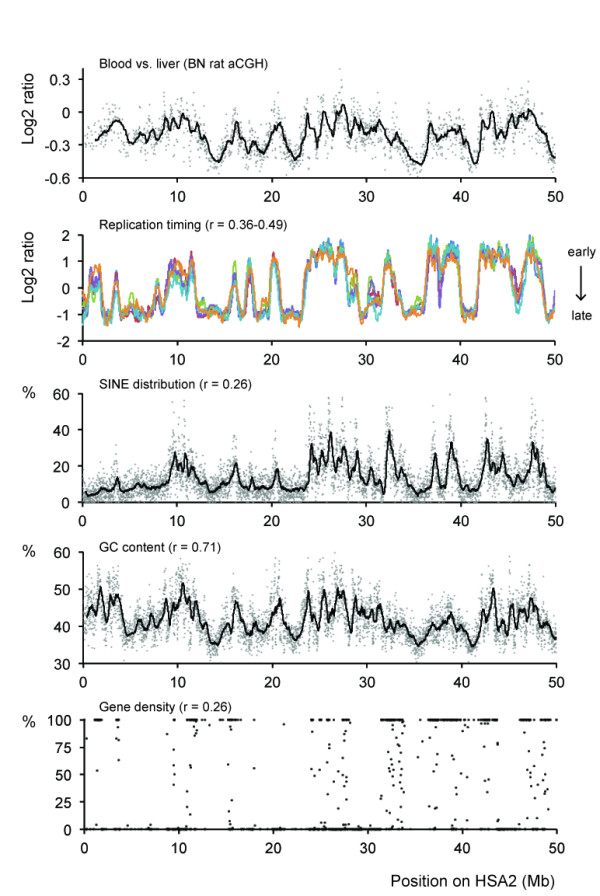
**Correlation of DNA content variability with genome characteristics**. The middle panel shows aCGH results comparing BN blood *versus *BN liver DNA and is aligned with replication timing (early replication is represented by high values, data obtained from Ryba *et al. *[11]), SINE distribution, GC content (100 kb windows), and gene density. Pearson correlation scores (r) are given per comparison. For each, *P *values are < 0.001. For this visualization, genomic positions of rat aCGH data were translated to positions on the first 50 Mb of human chromosome 2 (HSA2) to be able to compare rat data with human data on replication timing.

**Figure 3:**
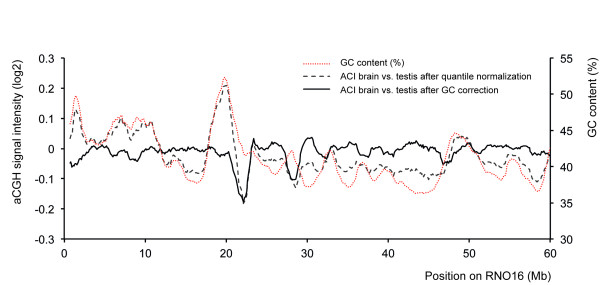
**GC correction reduces, but does not diminish tissue-specificity in aCGH signal intensity**. The red line depicts the GC content in percentage (secondary y-axis) across rat chromosome 16. The dashed black line shows the log2 aCGH signal intensity of brain *versus *testis (ACI rat strain). The black line shows the signal intensity after GC correction. Specific peaks at high GC regions are visibly removed (for example, at 18 Mb), while others are not (for example, at 28 Mb).

Systematic analysis of genomic regions with tissue-specific differences in aCGH hybridization and DOC signal revealed several interesting characteristics. A very clear correlation was found with replication timing [11], gene density, presence of SINE elements, and the relative GC content, which is strongly related to isochores [12,13] (Figure 2, Additional file 2). GC content has been documented to affect a wide range of molecular biological techniques, including PCR and next-generation sequencing [6] and may thus explain part of the observed patterns. Regional high GC content was recently described to affect the thermostability of DNA, resulting in ultra-fastened regions that affect amplification [14]. However, as DNA content is assumed to be largely the same in every cell, the GC content alone cannot explain the observed tissue-specific patterns or differences in signal magnitude (Figure 1). When we perform a GC correction on the aCGH data, this flattens out large parts of the pattern, as expected based on the high correlation with GC. However, the GC correction alone is insufficient to flatten the profile between different tissues from the same animal (Figure 3). As we used asynchronous whole tissue samples with only a very small amount of actively proliferating cells, early replicating genomic regions are also unlikely the cause of apparent copy number gains. Intriguingly, replication timing has been shown to correlate with retrotransposon content, genome isochores, and gene expression activity [15], and all of these factors are known to be highly related to chromatin status. Therefore, we hypothesized that tissue-specific chromatin organization may explain the observed correlations and that non-equimolar representation might be due to DNA retrieval artifacts that result in differential representation of euchromatin compared to more densely packed heterochromatin. In support of this, we do observe prominent tissue-specific gene expression in regions with higher apparent tissue-specific copy number status (Additional file 3).

**Figure 4:**
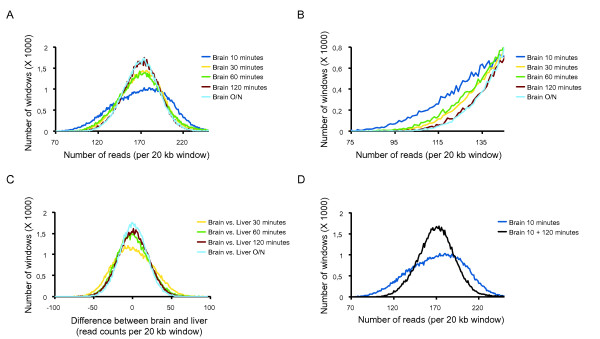
**The duration of proteinase K exposure affects the evenness of genome-wide read distribution**. (**A**) For five different durations of lysis (10, 30, 60, 120 min, and overnight (O/N)), the evenness of coverage is determined by calculating the number of 20 kb windows and the number of sequencing reads therein. The y-axis displays the genome-wide number of windows (X 1,000) while the x-axis depicts the number of quantile normalized reads. The width of the curve shows the genome-wide variation in read-depth between all windows. (**B**) Zoomed-in region of (A) to illustrate the read differences in windows with relatively difficult to cover genomic regions. (**C**) Genome-wide tissue-specific differences in read-depth per window are displayed for brain and liver at four different durations of lysis (30, 60, 120 min, and overnight). (**D**) Comparison of the genome-wide read distribution for a sample treated 10 min with proteinase K (blue line), and the exact same DNA sample after 120 min of extra proteinase K treatment (black line).

To study a potential bias resulting from differential chromatin status and introduced during the DNA isolation procedure, we first isolated DNA using standard phenol/chloroform extraction procedures and a commercial DNA isolation kit. aCGH was used to measure potential differences in relative DNA content between the two extraction methods but no significant differences were observed (Additional file 4). Next, we modulated the stringency of extraction by varying proteinase K treatment conditions prior to phenol/chloroform extraction, and used NGS fragment sequencing to determine the DNA recovery patterns across the whole genome. We compared five different lysis durations in the presence of proteinase K and observed that increased duration of treatment improved the evenness of read distribution across the whole genome (thus lowering the wave-amplitude; Figure 4A). Especially in the more difficult to cover regions the increased treatment duration improved coverage (Figure 4B). Next, we determined if the increased duration of the treatment also reduced the tissue-specific differences as depicted in Figure 1. By comparing sequenced DNA from homogenized brain and liver samples of the same animal at four time points, we indeed find that an increased lysis time results in smaller tissue-specific differences (Figure 4C), although it should be noted that biases are not removed. In agreement with our previous observations, the results of copy number profiling of brain and liver samples are affected by proteinase K treatment duration, even after GC correction. While segments totaling to 45 Mb show copy number differences of at least 10% after a 30-min proteinase K treatment, only 1.3 Mb exhibit changes of this scale when treatment is done overnight (Additional file 5).

These results demonstrate that the observed wave patterns are the result of combined tissue-specific DNA isolation biases. As the magnitude, but not the pattern, of the biases decreases with longer proteinase K treatment (Additional file 6), we postulate that DNA retrieval effects are due to differences in degradation of DNA/protein complexes, which subsequently results in depletion of stable aggregates by early precipitation or separation into the phenol phase. Densely packed heterochromatic regions, but also nuclear lamina attached chromatin, are likely to be most affected by such process.

To test whether the DNA in the under-represented genomic regions was simply absent from the sample, or just inaccessible for subsequent applications like sequencing or aCGH, we modulated the DNA isolation experiments even further. First, DNA was extracted after only 10 min of lysis in the presence of proteinase K. After one initial round of phenol/chloroform extraction and precipitation, the sample was divided in half. One part was treated with proteinase K for 2 additional hours, while the other was used as a control and left untreated. We subsequently extracted the DNA from both samples using a second round of phenol/chloroform extraction. Surprisingly, the NGS data show that an additional 2 h of treatment dramatically improves the evenness of the genome-wide coverage as compared to the control sample (Figure 4D), now resembling the read distribution of samples that were treated for a minimum of 2 h. This suggests either that inaccessible DNA was present after the first phenol/chloroform extraction and made accessible by the additional proteinase K treatment, or that the second phenol/chloroform purification step removed additional protein-bound DNA from the control sample. In any case, these experiments further demonstrate that equimolar DNA representation is affected by differences in DNA isolation conditions.

## Conclusions

We demonstrate that DNA isolation procedures can introduce a systematic bias that contributes to the wave effects in aCGH data and the variation in coverage depth in NGS data. We show that extended lysis with proteinase K treatment results in: (1) more even representation of NGS reads across the genome; (2) more similar representation of DNA derived from different tissue sources; and (3) improved DNA content uniformity for a previously undertreated DNA sample. Our data show that the basis for the observed bias is tissue-specific and related to specific chromatin characteristics. Interestingly, from the four tissues that we sampled in this study, brain showed the lowest variation in NGS read coverage. This could reflect the diversity of cell types within this tissue and the associated increased variety of chromatin conformations. More homogeneous tissues like blood and liver exhibited the largest bias in read coverage (Additional file 7), again supporting a cell type-specific origin of the effects rather than primary DNA characteristics. Tissue-specific chromatin characteristics could originate from protein-DNA interactions, 3D organization, and epigenetic modifications.

The observations presented in this study are relevant for a wide range of genomics techniques. Obviously, the described artifacts affect the accuracy of CNV detection [16,17], in particular somatic CNV analyses such as in cancer where sample heterogeneity requires accurate detection of relatively small changes. Furthermore, genome-wide nucleotide variation analyses using next-generation sequencing may also be affected, as depleted regions will have lower sequencing coverage, which results in lower reliability of variant calling. Accurate experimental reflection of the original amounts of DNA is also important for other genomics techniques, as was recently demonstrated for ChIP-seq experiments [18]. As none of the methods or conditions tested could completely remove the signal bias, special care should be taken to control for potential DNA isolation and tissue-specific effects in experiments involving quantitative DNA interpretation. Furthermore, detection of somatic copy number variation will require independent measurements, for example, using allele imbalances [7,8].

## Materials and methods

### Isolation of genomic DNA

Tissues were collected from BN (BN/Crl, Charles River), WU (HsdCpb:WU), and ACI rats (ACI/Seg/Hsd, Harlan Laboratories B.V., The Netherlands) 6 weeks of age, snap frozen and powdered. A total of 30 mg input material was used for strain and tissue comparisons, 100 mg input material was used for DNA isolation methods comparisons. DNA was isolated using standard phenol/chloroform extraction (1:1, pH 7.9) or Qiagen DNeasy Blood & Tissue kit (cat.no. 69506). Tissues were lysed in lysis buffer (100 mM Tris-HCI pH 8.0 200 mM NaCl, 0.2% SDS, 5 mM EDTA) using a Kontes Dounce tissue grinder (Kimble and Chase, 885300-0002) and incubated for 2 h at 50°C in the presence of RNase A (50 μg/mL) and proteinase K (100 μg/mL). For WU rat brain and liver, additional proteinase K conditions were tested (10 min, 30 min, 60 min, 120 min, and overnight in lysis buffer). These time series were followed by two rounds of standard phenol/chloroform extraction with in-between precipitation of the DNA (1x phenol, 1x phenol/chloroform, and 1x chloroform). DNA precipitation was done with 3 volumes of pure ethanol in the presence of 1/10 volume sodium acetate (3 M). Pelleted DNA was washed with 70% ethanol and dissolved in 10 mM Tris pH 8.0. For the additional proteinase K treatment experiment, 50% of the DNA that was extracted after a 10-min lysis was treated for an additional 120 min of proteinase K (100 μg/mL) in the lysis buffer described above. Next, both samples were cleaned during a second round of phenol/chloroform and ethanol precipitation, similar to the other samples in the time series.

For isolation of blood DNA with the Qiagen kit, all steps were performed exactly according to manufacturer's instructions (Qiagen DNeasy Blood & Tissue handbook, 07/2006). DNA quality and quantity of all isolations were measured using NanoDrop ND-1000 (Thermo scientific) and a Qubit Quant-iT™ dsDNA broad range assay (Invitrogen).

### Array comparative genomic hybridization (aCGH)

NimbleGen whole genome tiling path arrays covering the complete, non-repetitive part of the rat genome were used. The 2.1 M probe arrays had an average probe spacing of 1 probe per 1.3 kb and a GC-content close to 50%. For strain and tissue comparisons, DNA derived from tissues of BN and ACI rats was used for hybridization. The exact quantity of DNA recommended by NimbleGen was used (2 μg input for sonication, 1 μg input for exo- klenow mediated Cy3 and Cy5 labeling, 13 μg for hybridization). DNA labeling (NimbleGen dual-color DNA labeling kit), array hybridization (HX1 mixers, NimbleGen hybridization system 4), washing (NimbleGen wash buffer kit), and scanning were performed exactly according to manufacturer's instructions (NimbleGen Arrays User's Guide - CGH analysis Version 6.0). Image analysis, data normalization, and plotting were performed using NimbleScan 2.4 software using parameters preset by the manufacturer. For platform and extraction method comparisons, Agilent custom designed tiling path arrays (4 × 44 k, ± 1.5 kb probe spacing) were designed for the complete rat chromosome 14 (RNO14). aCGH DNA preparation steps and array hybridization were performed according to manufacturer's instructions (Agilent Oligonucleotide Array-Based CGH for Genomic DNA Analysis V4.0).

### SOLiD mate-pair sequencing and depth of coverage analysis for the sequencing aCGH comparison

For the mate-pair sequencing data presented in Figure 1, 118 microgram of genomic DNA was fragmented by incomplete digestion during a time series of 15 s to 25 min with the *Alu I *restriction enzyme (Promega, R6281). Time points were pooled and the fragmented DNA was loaded on a 1% agarose gel for gel excision of 1-3 kb fragments. Mate-pair libraries were prepared according to the Applied Biosystems User's Guide (12/2007 4391587 Rev. B) and sequenced on SOLiD V2. Sequencing data were mapped against Rat genome assembly RGSC3.4 by BWA 0.5.9 software [19] (parameters -c -l 25 -n 2 -k 6). We calculated the number of reads using genomic windows containing 100 kb of genome sequence (excluding sequence gaps). The number of reads in each sample was normalized as reads per million sequenced reads. These normalized values were used for the calculation of log ratios and plotting. In total, 6,325,428 reads were used for blood, 6,600,672 for brain, 6,764,588 for liver, and 7,183,059 for testis. These numbers equal a low-pass genome coverage ranging between 0.075× and 0.086×.

### SOLiD fragment sequencing and depth of coverage analysis for the time series

For the time series experiments presented in Figure 5, barcoded fragment libraries were produced on an automated system (BioMek), introducing no variation in the library preparation procedure. One microgram of DNA was used as input and libraries were prepared exactly according to manufacturer's instructions for SOLiD 5500XL library preparation. SOLiD libraries were pooled equimolary, quality assessed, and size selected on the Caliper XT system. Sequencing reads were aligned to the Rat reference genome RGSC3.4 using BWA 0.5.9 [19] (parameters -c -k 2 -l 25 -n 10). PCR duplicates were marked in the alignments and were not used in the analysis, resulting in 10 to 35 M unique and unambiguously mapped reads per time point. For tissue comparisons, the coverage of each library was normalized by random removal of reads to 10 M of unambiguously mapped tags (0.2× genome coverage), which corresponds to the liver library with the least amount of mapped reads. For the additional proteinase K treatment comparisons, only brain samples were used and these could thus be normalized to 14.8 M reads (0.3× genome coverage; limited by the brain library with the least number of reads).

The genome was partitioned into windows each containing 20 kb of NGS-accessible sequence (excluding repeats and gaps). The read count and GC content were determined for each window and library. GC-correction: read counts were adjusted for each library by normalization against the median read count in 100 genomic windows with most similar GC content using the following formula: N_corr _= N_med_*N_obs_/N_medGC _where: N_corr_, GC-corrected number of reads; N_obs_, observed number of reads; N_med_, median reads per window for this library; and N_medGC_, median number of reads in 100 windows with the most similar GC content. After GC correction, potential somatic copy number changes were determined using a dynamic window approach (DWAC-seq).

### Correlation of DNA content variability with various genome characteristics and gene expression

GC content, repeat, and gene annotation were extracted from the Ensembl database [20] (v.69). Gene expression data were exported from the UCSC genome browser [21].

## Abbreviations

Array CGH/aCGH: array-comparative genome hybridization; ChIP-seq: chromatin immunoprecipitation sequencing; CNV: copy number variation; DOC: depth of coverage; Mb: megabase; MLPA: multiplex ligation-dependent probe amplification; NGS: next-generation sequencing; PCR: polymerase chain reaction; SINE: short interspersed nuclear element.

## Competing interests

The authors declare no competing interests.

## Authors' contributions

SvH, MM, VB, WJ, RM, PM, EdB, and EC carried out the molecular biological studies. SvH, MM, VB, and EC performed DNA isolation for next-generation sequencing experiments, SvH, MM, WJ, and RM performed aCGH experiments. PT collected tissues and EdB performed next-generation sequencing. SvH and VG performed data analyses. EC and VG conceived of the study and coordinated experiments. JDS and TJA contributed reagents and data, critically discussed results and directions of the research and contributed to drafting the manuscript. SvH, EC, and VG wrote the manuscript. All authors read and approved the final manuscript.

## Data availability

All sequencing data are available from the Sequence Read Archive (SRA) at EBI under accession number (ERP001927). Array CGH data are available from the Gene Expression Omnibus (GEO) database at NCBI under accession number (GSE45308). Whole genome aCGH plots for tissue comparisons are available as Additional file 8 (ACI blood *versus *liver and brain *versus *testis; BN blood *versus *liver and liver *versus *blood).

## Supplementary Material

Additional file 1 **Additional data file 1 is a figure showing the genome-wide correlation between aCGH results and NGS read-depth**. Click here for file

Additional file 2 **Additional data file 2 is an Excel table listing all the correlations between the aCGH data and specific genome characteristics belonging to Figure **2**(replication timing, SINEs, gene density, GC content)**. Click here for file

Additional file 3 **Additional data file 3 shows the relation between tissue-specific aCGH patterns and gene expression data**. Click here for file

Additional file 4 **Additional data file 4 contains a figure illustrating that independent DNA isolation techniques (phenol-chloroform and a commercial column-based DNA isolation kit) have no effect on the aCGH pattern**. Click here for file

Additional file 5 **Additional data file 5 is an excel table listing all putative somatic CNV regions that could be identified by comparing brain and liver tissue after either 30 min or 60 min of proteinase K treatment**. Click here for file

Additional file 6 **Additional data file 6 shows the effects of increase proteinase K treatment on the wave pattern on rat chromosome 16 (30 min *versus *overnight treatment)**. Also, it shows all time points on a genome-wide scale compared to a fully random read distribution. Click here for file

Additional file 7 **Additional data file 7 is a figure showing that NGS read coverage depends on the homogeneity of a tissue, independent of the GC content**. Click here for file

Additional file 8 **Additional data file 8 provides additional genome-wide aCGH plots for the tissue and strain comparisons presented in Figure **1A. Click here for file
