# Supplementary material for: Systematic biases in DNA copy number originate from isolation procedures
Source: Genome Biol. 2013 Apr 24;14(4):R33. doi: 10.1186/gb-2013-14-4-r33 (PMC4054094; doi:10.1186/gb-2013-14-4-r33)
Supplement: Additional file 1 — Additional data file 1 is a figure showing the genome-wide correlation between aCGH results and NGS read-depth. [file gb-2013-14-4-r33-S1.PDF]

Additional file 1

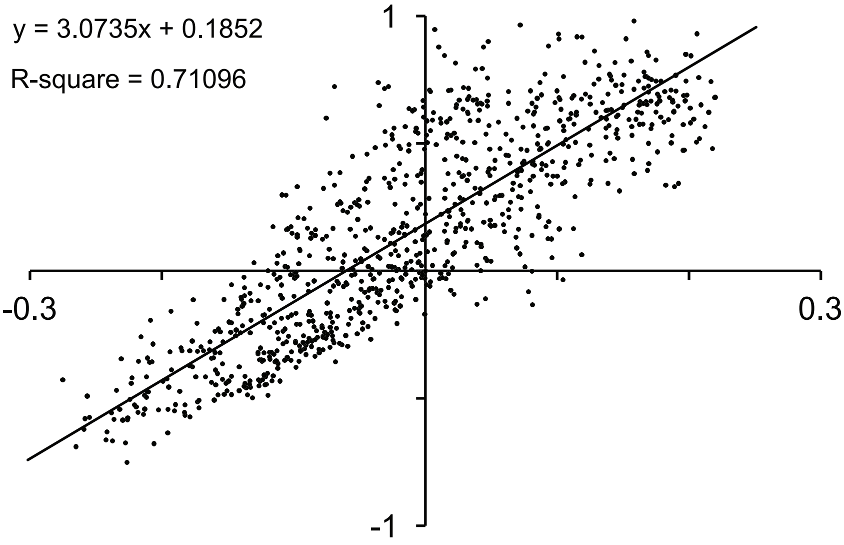

Additional file 1) Genome-wide correlation plot of NGS read depth and aCGH signal intensity.
Log2 ratios of NGS depth of coverage differences (y-axis) are plotted against log2 ratios for aCGH data (x-axis) for window sizes of 100 kb resulting in an R2 value of 0.71.
