# Supplementary material for: Systematic biases in DNA copy number originate from isolation procedures
Source: Genome Biol. 2013 Apr 24;14(4):R33. doi: 10.1186/gb-2013-14-4-r33 (PMC4054094; doi:10.1186/gb-2013-14-4-r33)
Supplement: Additional file 3 — Additional data file 3 shows the relation between tissue-specific aCGH patterns and gene expression data. [file gb-2013-14-4-r33-S3.PDF]

Additional file 3

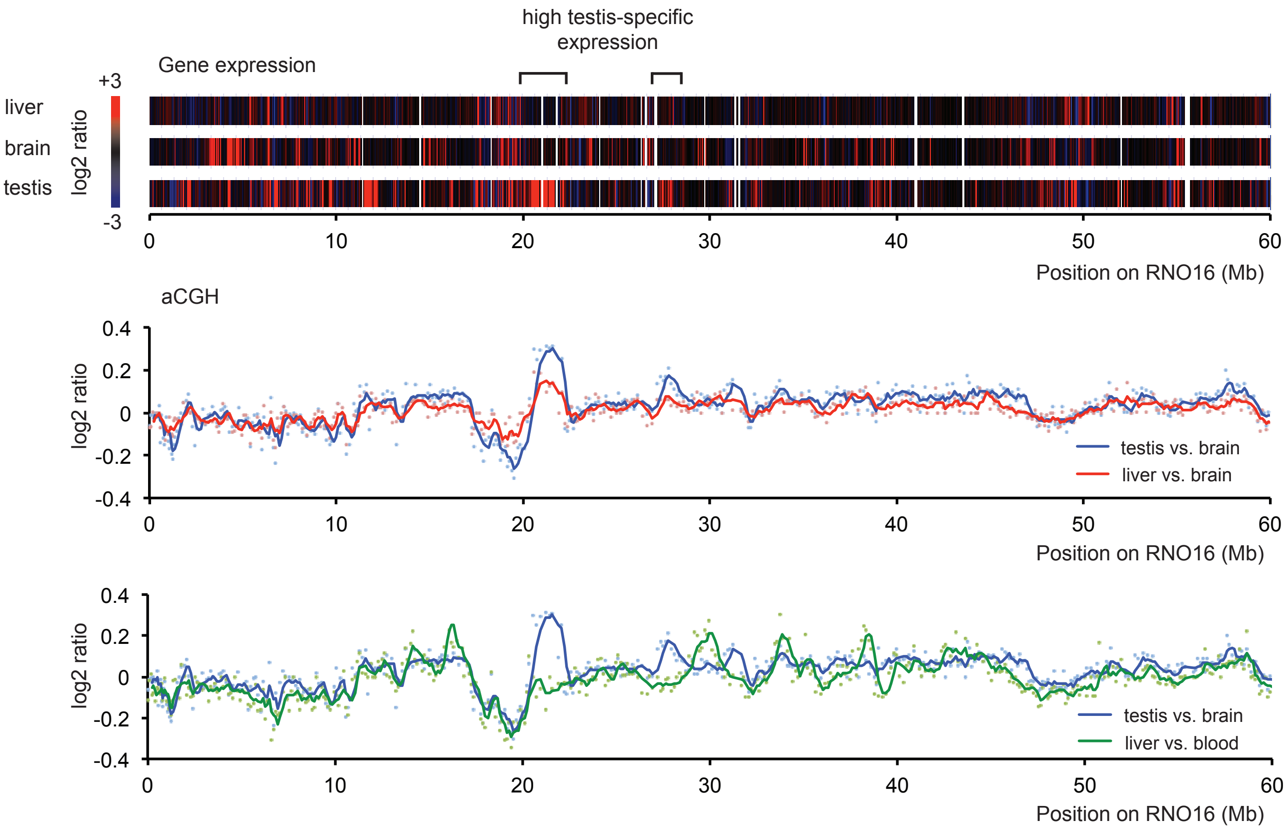

Additional file 3) Overlap of intensity variation in the DNA pattern with tissue-specific gene expression profiles. The top panel shows gene expression levels (obtained by Affymetrix exon-arrays) from liver, brain and testis, as derived from the UCSC genome browser (Pohl, Bioinformatics, 2009). Expression data for blood were not available. Log2 expression ratios varying between +3 (highly expressed, red) and -3 (lowly expressed, blue) are visualized along rat chromosome 16. Below the expression track aCGH hybridization signals for ACI rat testis versus brain (blue), liver versus brain (red) and liver versus blood (green) are shown. Two regions with high testis-specific gene expression that clearly correspond to increased aCGH signal intensities are indicated above the expression tracks.
