# Supplementary material for: Systematic biases in DNA copy number originate from isolation procedures
Source: Genome Biol. 2013 Apr 24;14(4):R33. doi: 10.1186/gb-2013-14-4-r33 (PMC4054094; doi:10.1186/gb-2013-14-4-r33)
Supplement: Additional file 4 — Additional data file 4 contains a figure illustrating that independent DNA isolation techniques (phenol-chloroform and a commercial column-based DNA isolation kit) have no effect on the aCGH pattern. [file gb-2013-14-4-r33-S4.PDF]

Additional file 4

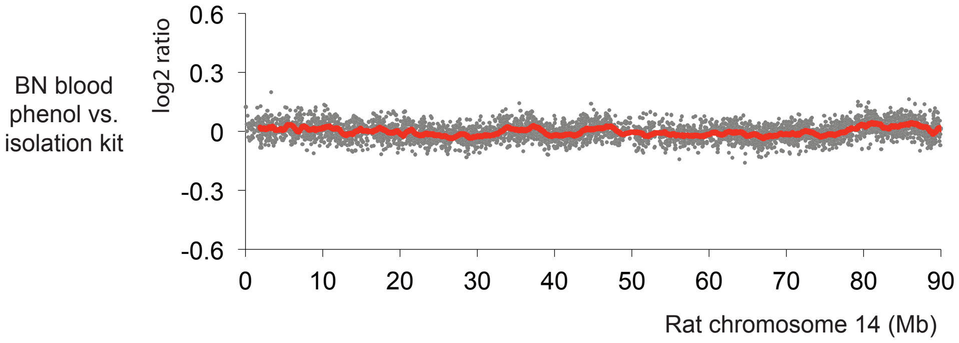

Additional file 4) DNA extraction methods do not contribute to the observed differential DNA representation. Phenol-chloroform (pH 7.9) extracted DNA from blood and column-based extracted DNA were hybridized against aCGH tiling arrays of rat chromosome 14. Scaling of the log2 ratio (y-axis) is identical to figure 1a.
