# Supplementary material for: Systematic biases in DNA copy number originate from isolation procedures
Source: Genome Biol. 2013 Apr 24;14(4):R33. doi: 10.1186/gb-2013-14-4-r33 (PMC4054094; doi:10.1186/gb-2013-14-4-r33)
Supplement: Additional file 6 — Additional data file 6 shows the effects of increase proteinase K treatment on the wave pattern on rat chromosome 16 (30 min versus overnight treatment). Also, it shows all time points on a genome-wide scale compared to a fully random read distribution. [file gb-2013-14-4-r33-S6.PDF]

Additional file 6

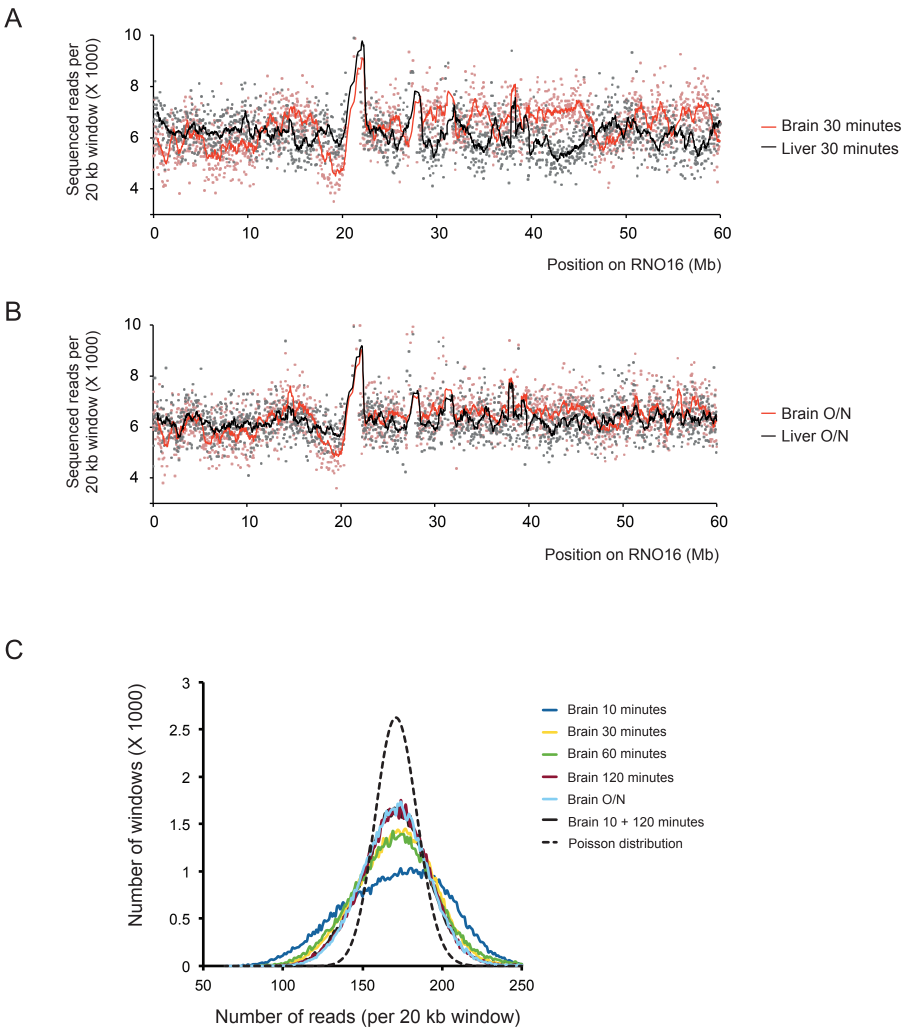

Additional file 6) Increased duration of lysis improves the patter similarity between tissues. (A) Fragment sequencing data of rat brain and liver is quantile normalized and plotted along rat chromosome 16. Both samples were lysed in the presence of proteinase K for 30 minutes before DNA was extracted. The y-axis shows the number of reads per 20 kb window (x 1000). (B) Again, fragment sequencing data was generated for rat brain and liver, but this time after an over night incubation in lysis buffer in the presence of proteinase K. As in panel A, the pattern remains clearly visible. The tissue-specific differences, however, decrease when compared to shorter treatment times. (C) The genome-wide distribution of reads per 20 kb window, as compared to the poisson distribution. The latter shows the distribution of sequencing reads throughout the genome if this would be completely random. The longer proteinase K treatment files approach this ideal distribution best.
