# Supplementary material for: Systematic biases in DNA copy number originate from isolation procedures
Source: Genome Biol. 2013 Apr 24;14(4):R33. doi: 10.1186/gb-2013-14-4-r33 (PMC4054094; doi:10.1186/gb-2013-14-4-r33)
Supplement: Additional file 7 — Additional data file 7 is a figure showing that NGS read coverage depends on the homogeneity of a tissue, independent of the GC content. [file gb-2013-14-4-r33-S7.PDF]

Additional file 7

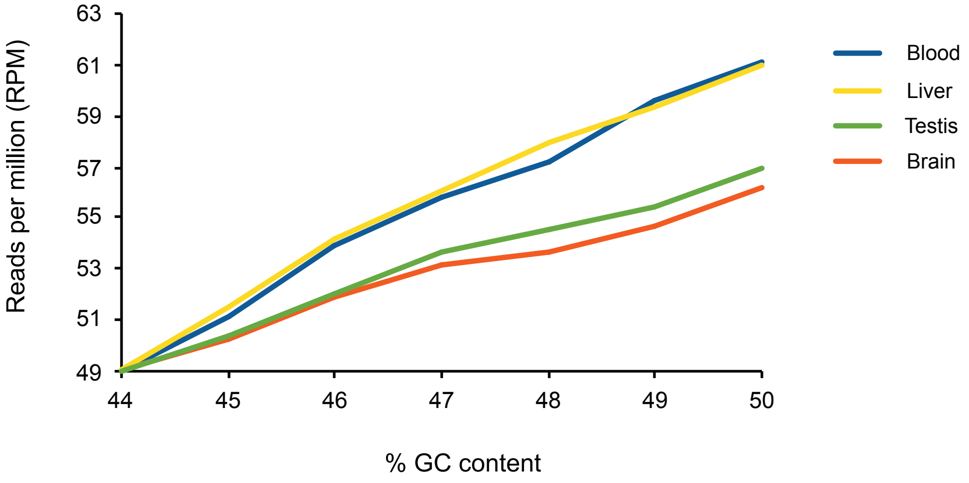

Additional file 7) NGS read coverage depends on tissue homogeneity. The median number of normalized reads per million (RPM) is displayed against the percentage of GC in 100 kb windows of the rat genome. Gaps in the reference assembly were excluded for this analysis. Tracks are displayed for two relatively homogeneous tissues (blood and liver) and two heterogeneous tissues (brain and testis). Changes in GC content show differential effects on both tissue types. A strong increase in RPM is observed for tissues consisting of relatively few cell types and a more mild increase in RPM is observed for complex tissues consisting of numerous cells with different functions, like the brain.
