# Supplementary material for: Systematic biases in DNA copy number originate from isolation procedures
Source: Genome Biol. 2013 Apr 24;14(4):R33. doi: 10.1186/gb-2013-14-4-r33 (PMC4054094; doi:10.1186/gb-2013-14-4-r33)
Supplement: Additional file 8 — Additional data file 8 provides additional genome-wide aCGH plots for the tissue and strain comparisons presented in Figure 1A. [file gb-2013-14-4-r33-S8.PDF]

Additional file 8) aCGH tissue comparison data plots for all chromosomes. This PDF contains chromosome plots for the BN and ACI tissue comparisons presented in figure 1A (ACI blood versus liver and brain versus testis; BN blood versus liver and liver versus blood). *See separate PDF file.*

chr1 segmentation for 16771102_532 vs. 16771102_635 (16771102_11000bp) - 78 segments

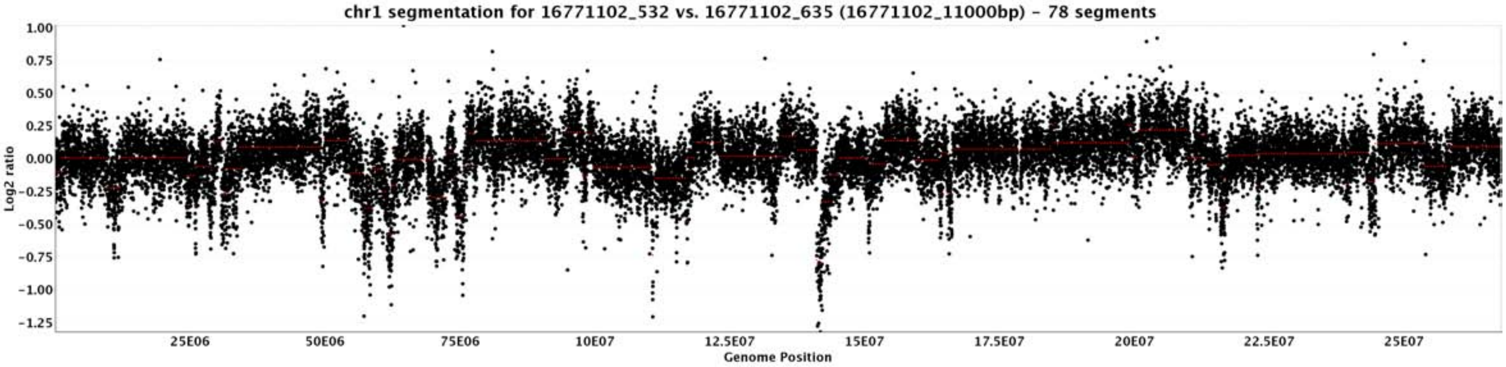

chr1_random segmentation for 16771102_532 vs. 16771102_635 (16771102_11000bp) - 3 segments

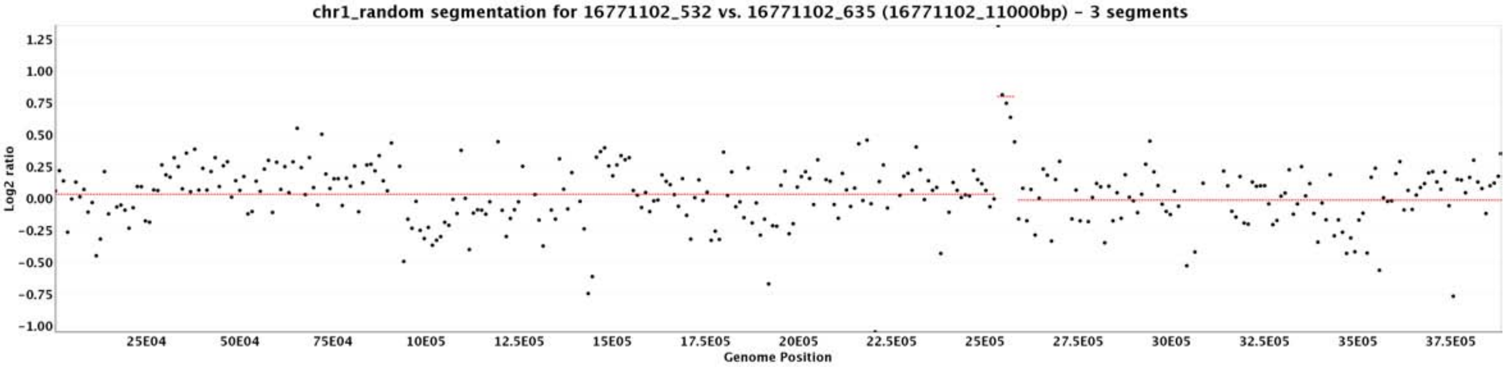

chr2 segmentation for 16771102_532 vs. 16771102_635 (16771102_11000bp) - 94 segments

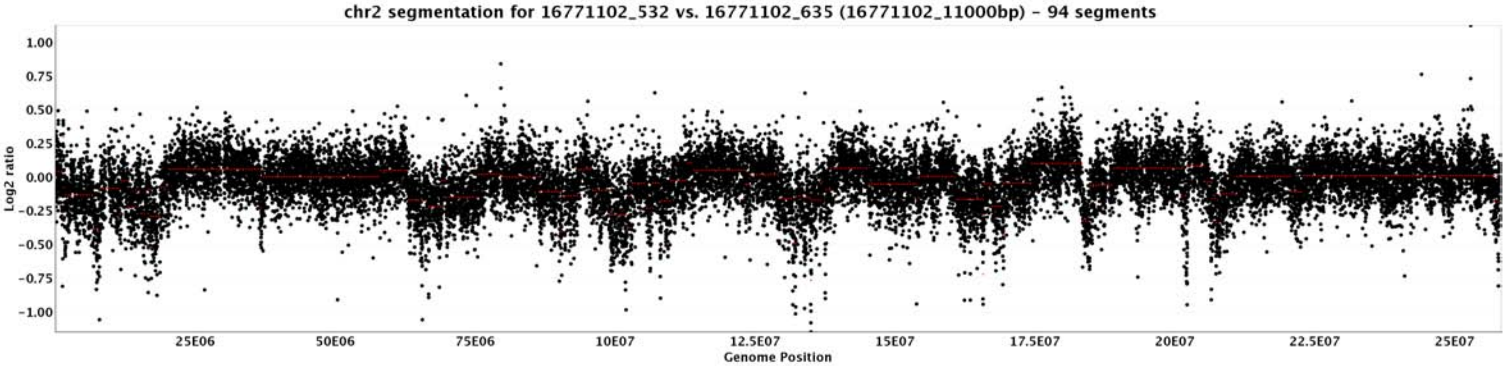

chr2_random segmentation for 16771102_532 vs. 16771102_635 (16771102_11000bp) - 3 segments

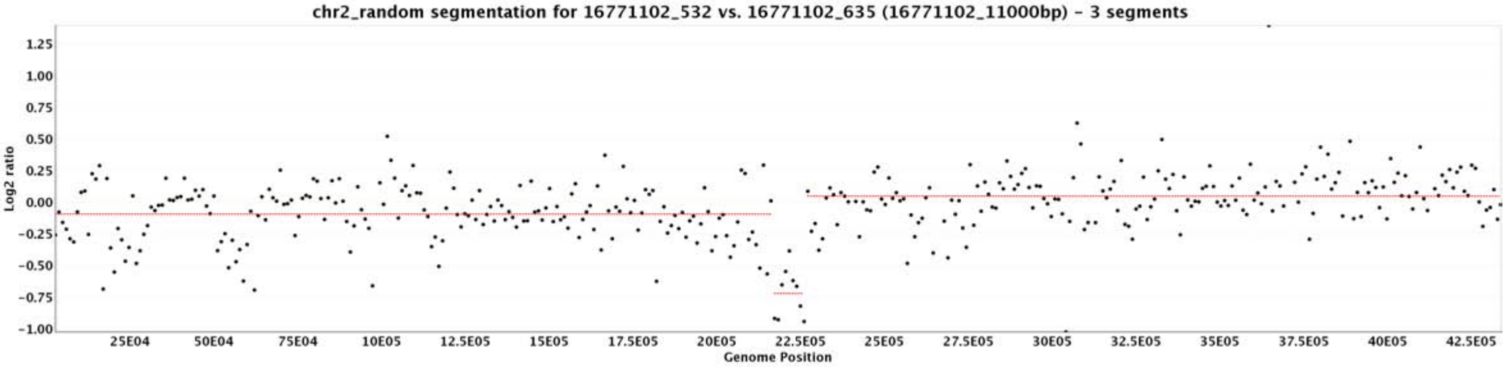

chr3 segmentation for 16771102_532 vs. 16771102_635 (16771102_11000bp) - 74 segments

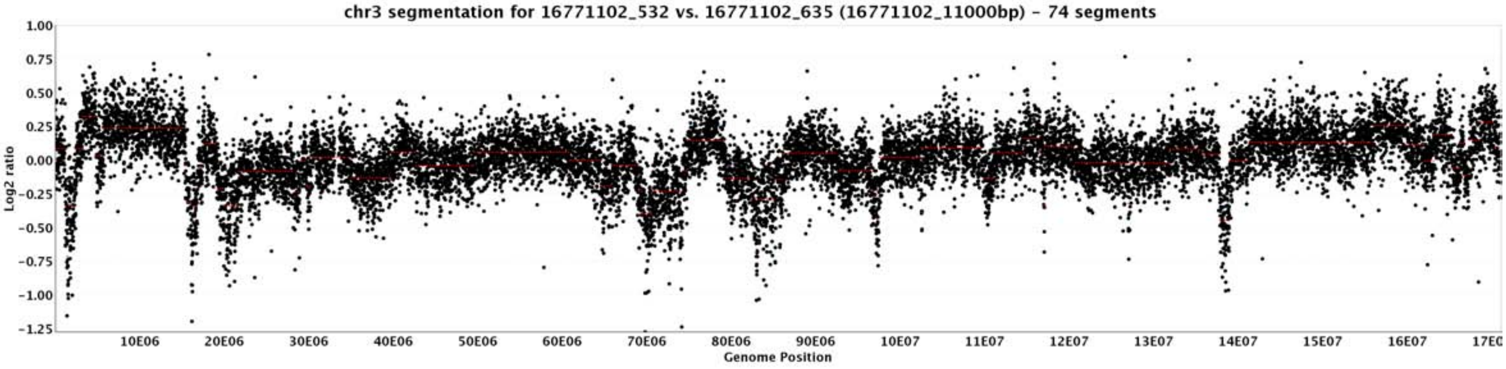

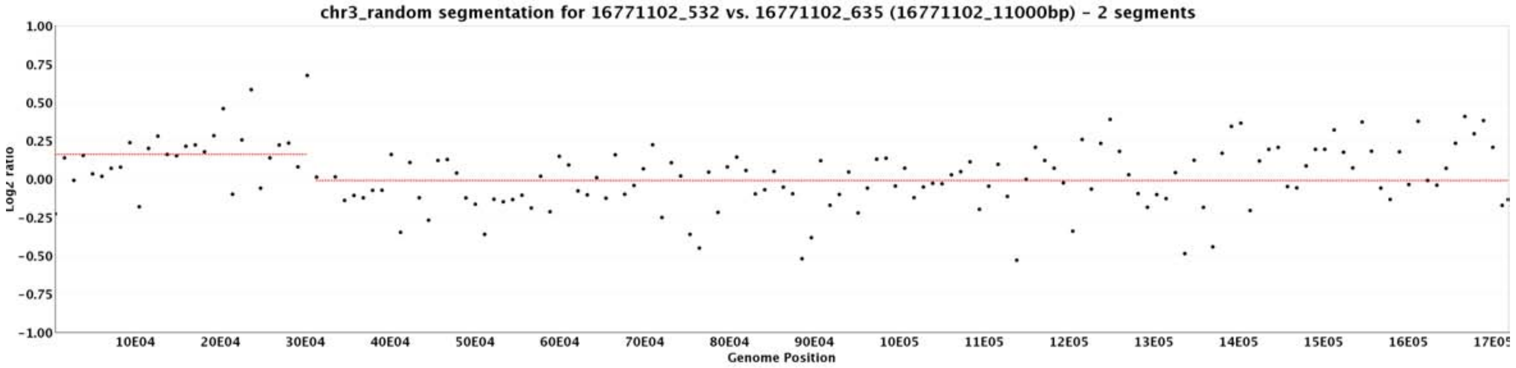
chr3_random segmentation for 16771102_532 vs. 16771102_635 (16771102_11000bp) - 2 segments
Log2 ratio
Genome Position

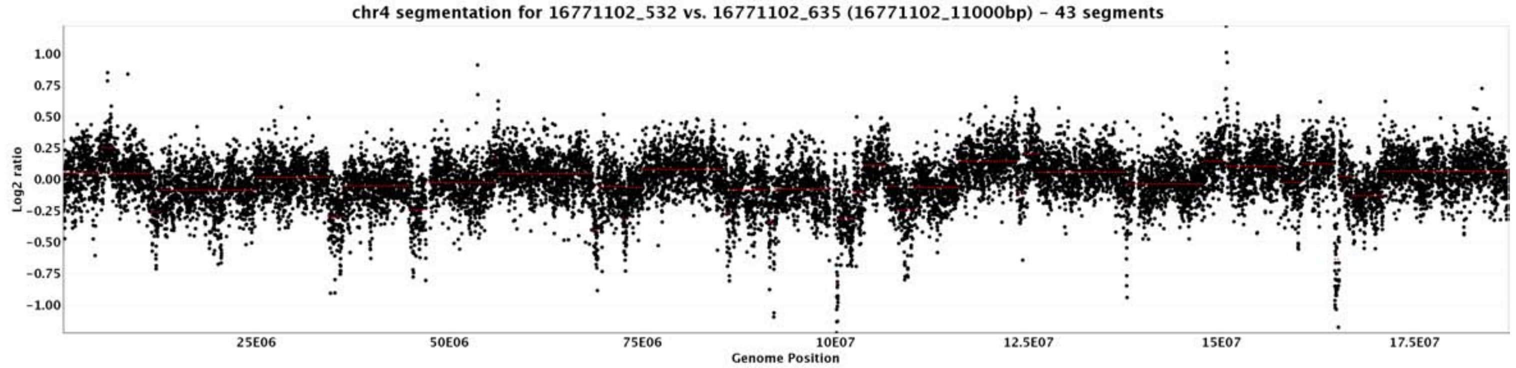
chr4 segmentation for 16771102_532 vs. 16771102_635 (16771102_11000bp) - 43 segments
Log2 ratio
Genome Position

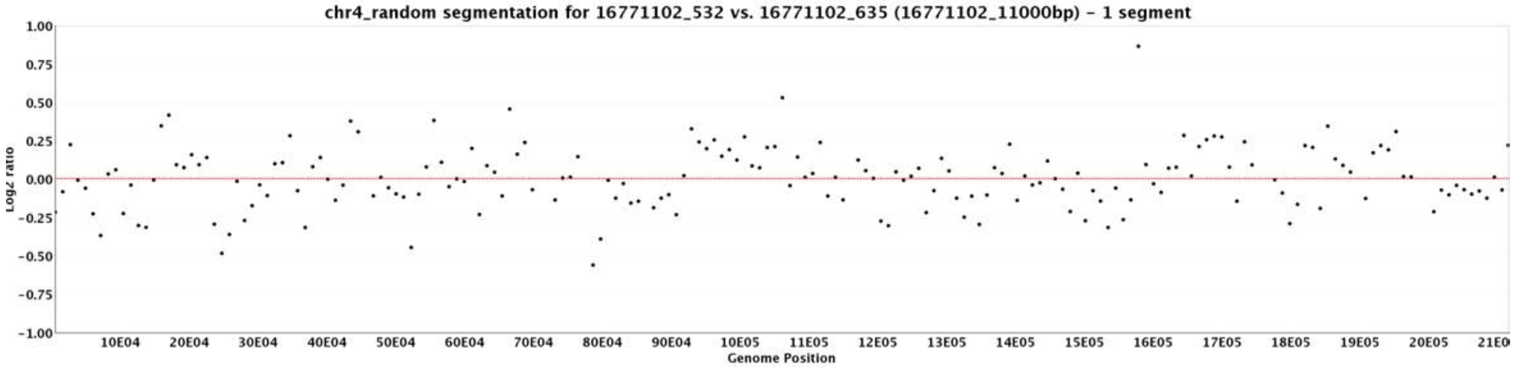
chr4_random segmentation for 16771102_532 vs. 16771102_635 (16771102_11000bp) - 1 segment
Log2 ratio
Genome Position

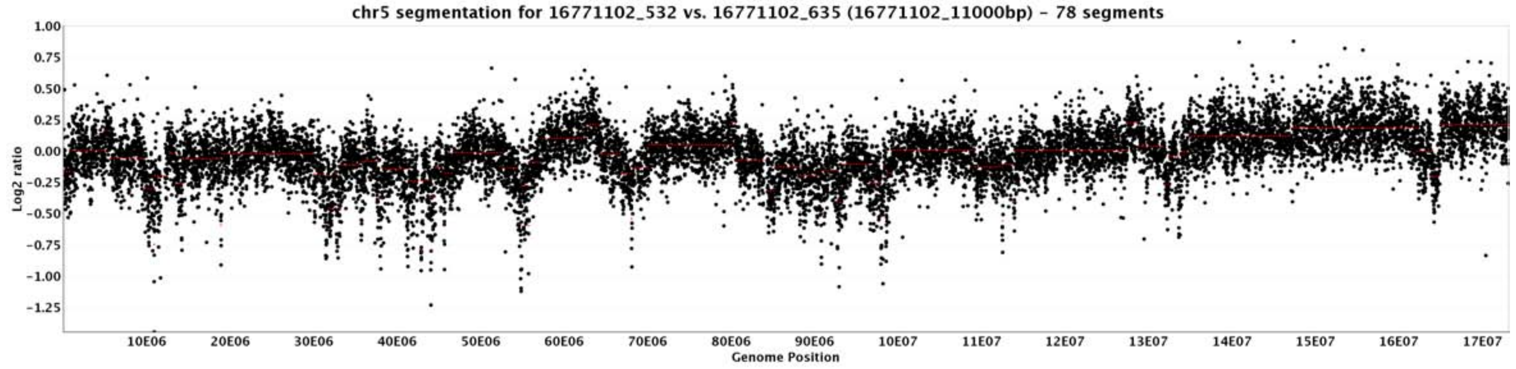
chr5 segmentation for 16771102_532 vs. 16771102_635 (16771102_11000bp) - 78 segments
Log2 ratio
Genome Position

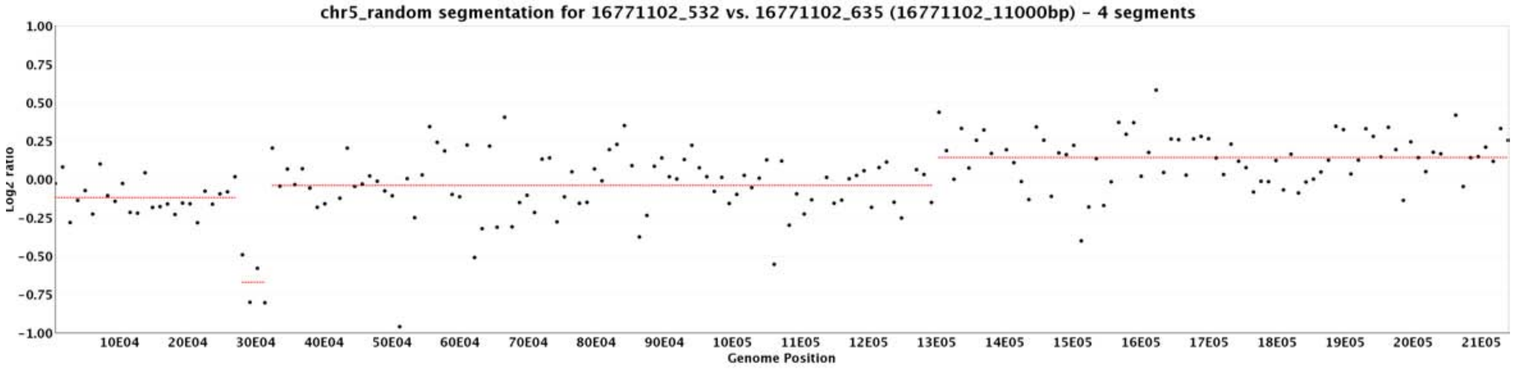
chr5_random segmentation for 16771102_532 vs. 16771102_635 (16771102_11000bp) - 4 segments
Log2 ratio
Genome Position

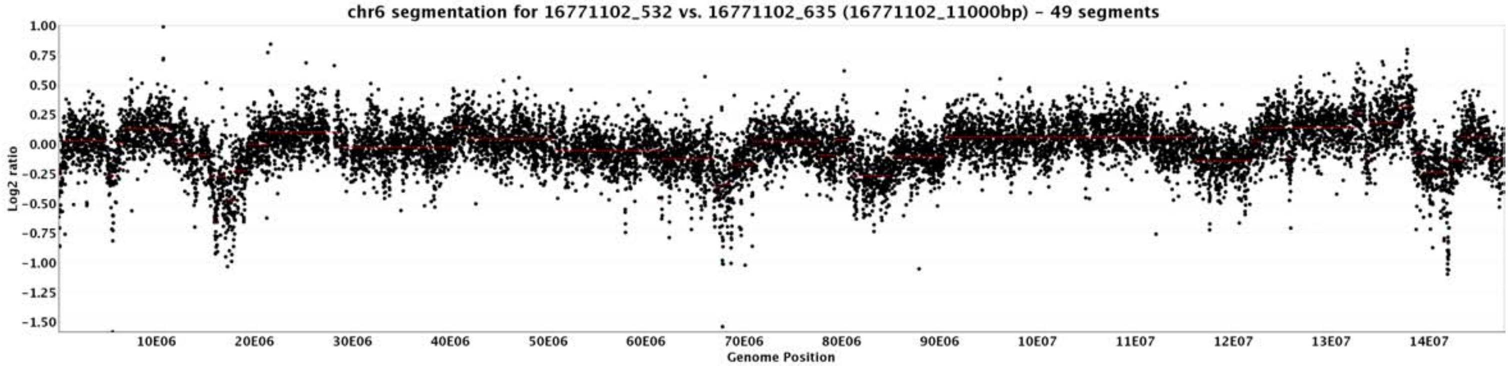
chr6 segmentation for 16771102_532 vs. 16771102_635 (16771102_11000bp) - 49 segments
Log2 ratio
Genome Position

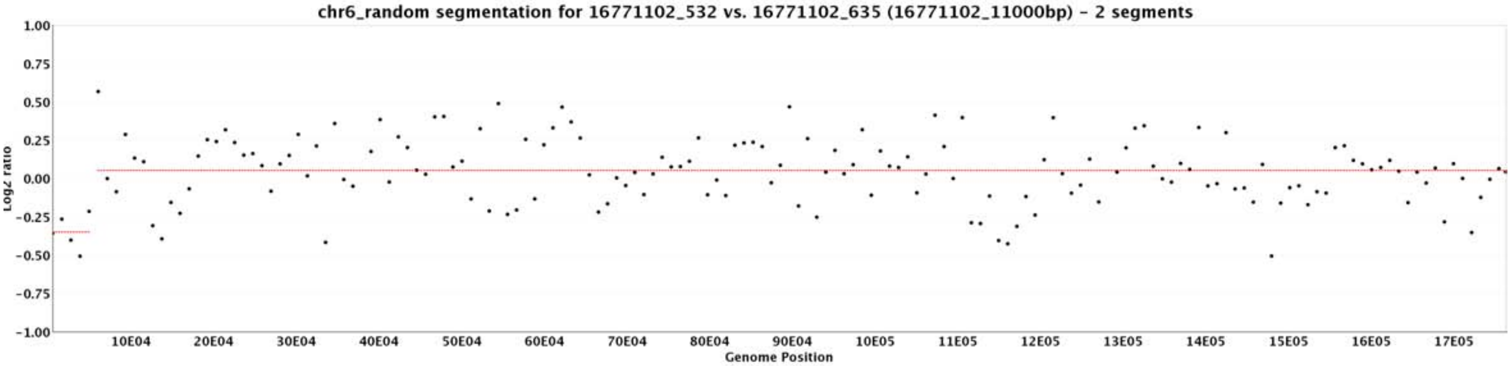
chr6_random segmentation for 16771102_532 vs. 16771102_635 (16771102_11000bp) - 2 segments
Log2 ratio
Genome Position

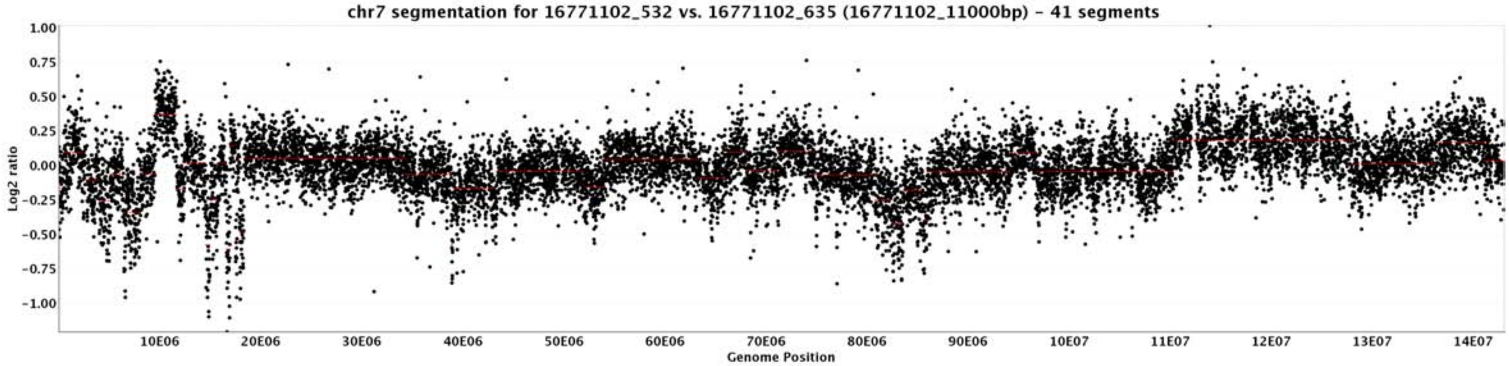
chr7 segmentation for 16771102_532 vs. 16771102_635 (16771102_11000bp) - 41 segments
Log2 ratio
Genome Position

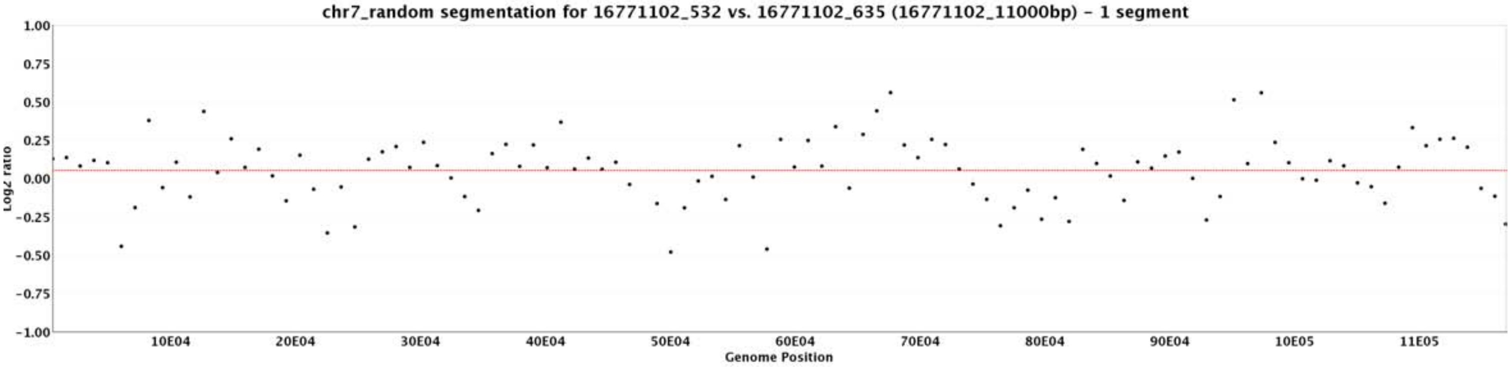
chr7_random segmentation for 16771102_532 vs. 16771102_635 (16771102_11000bp) - 1 segment
Log2 ratio
Genome Position

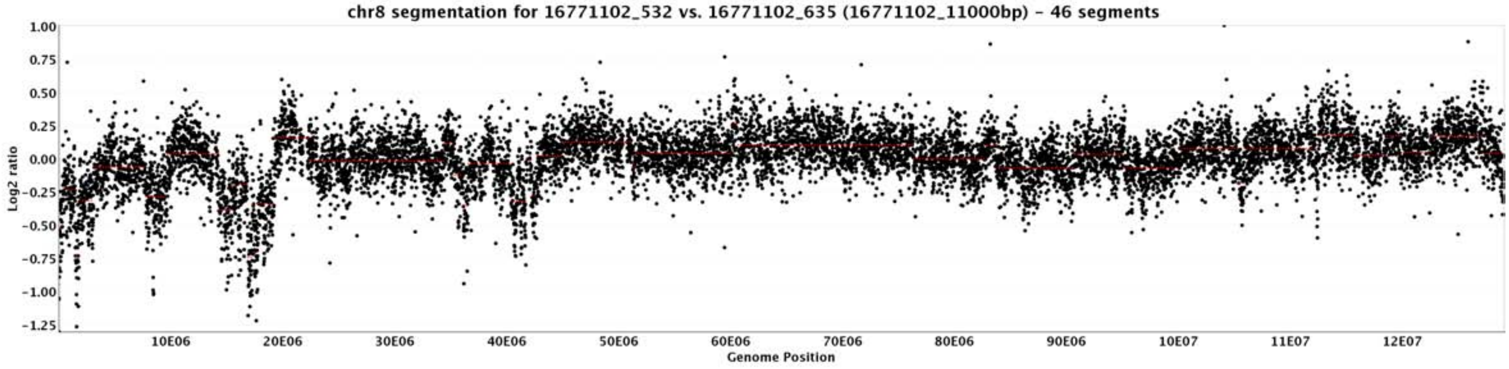
chr8 segmentation for 16771102_532 vs. 16771102_635 (16771102_11000bp) - 46 segments
Log2 ratio
Genome Position

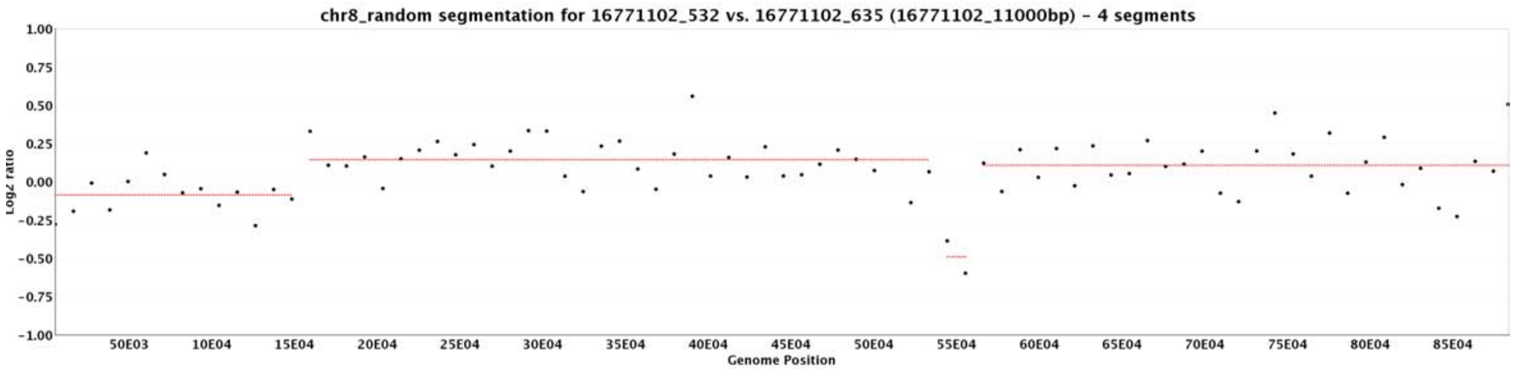
chr8_random segmentation for 16771102_532 vs. 16771102_635 (16771102_11000bp) - 4 segments
Log2 ratio
1.00 0.75 0.50 0.25 0.00 -0.25 -0.50 -0.75 -1.00
50E03 10E04 15E04 20E04 25E04 30E04 35E04 40E04 45E04 50E04 55E04 60E04 65E04 70E04 75E04 80E04 85E04
Genome Position

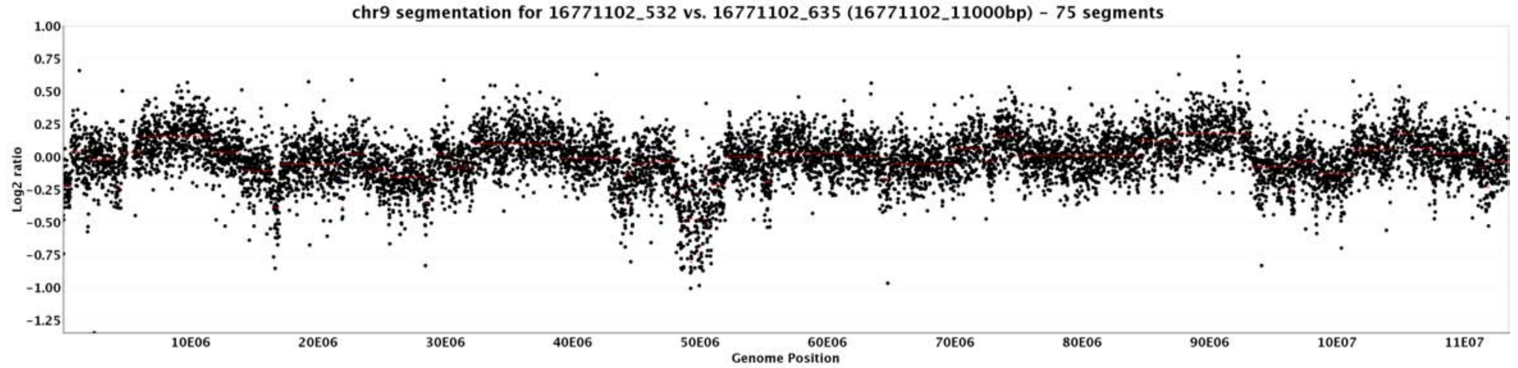
chr9 segmentation for 16771102_532 vs. 16771102_635 (16771102_11000bp) - 75 segments
Log2 ratio
1.00 0.75 0.50 0.25 0.00 -0.25 -0.50 -0.75 -1.00 -1.25
10E06 20E06 30E06 40E06 50E06 60E06 70E06 80E06 90E06 10E07 11E07
Genome Position

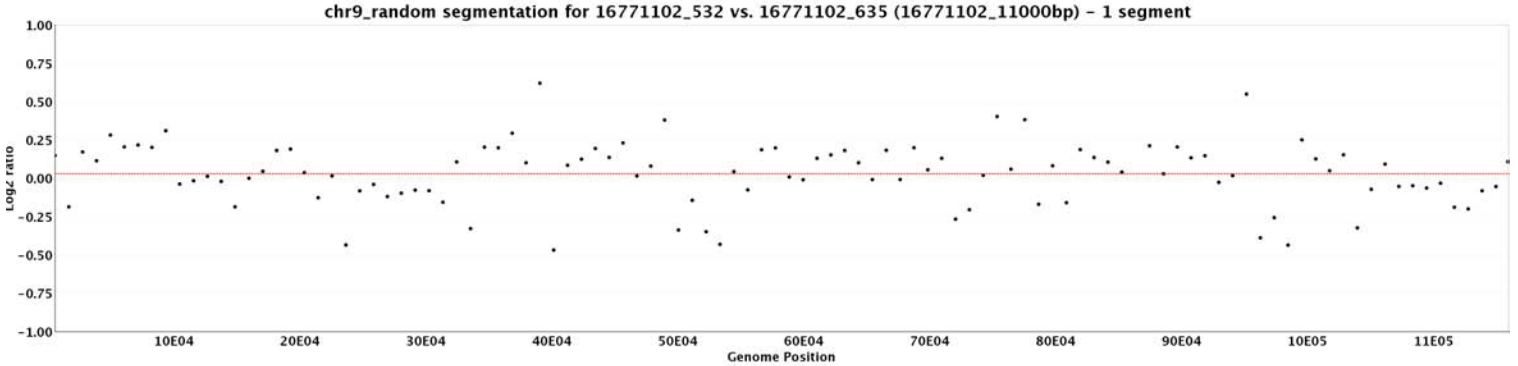
chr9_random segmentation for 16771102_532 vs. 16771102_635 (16771102_11000bp) - 1 segment
Log2 ratio
1.00 0.75 0.50 0.25 0.00 -0.25 -0.50 -0.75 -1.00
10E04 20E04 30E04 40E04 50E04 60E04 70E04 80E04 90E04 10E05 11E05
Genome Position

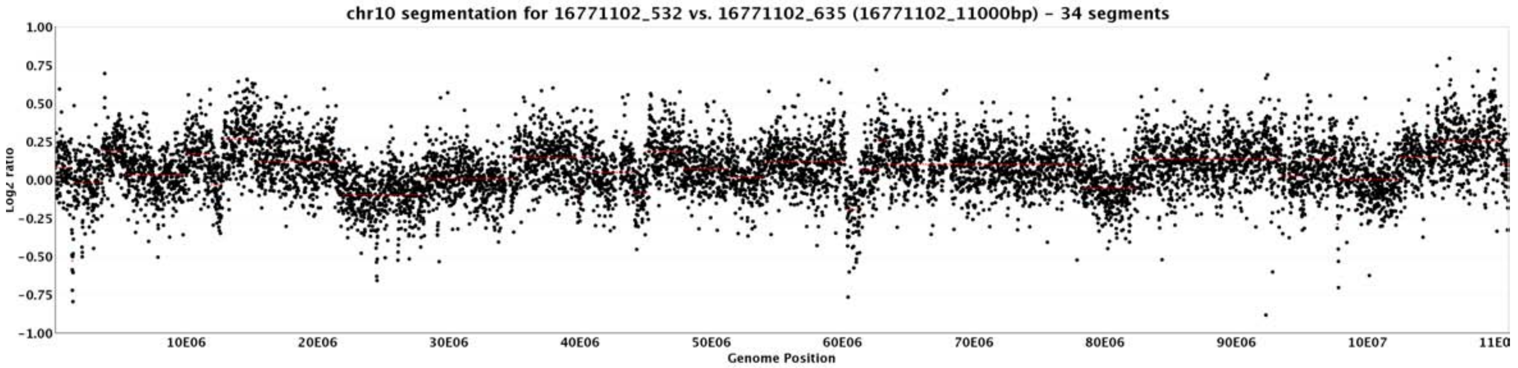
chr10 segmentation for 16771102_532 vs. 16771102_635 (16771102_11000bp) - 34 segments
Log2 ratio
1.00 0.75 0.50 0.25 0.00 -0.25 -0.50 -0.75 -1.00
10E06 20E06 30E06 40E06 50E06 60E06 70E06 80E06 90E06 10E07 11E0
Genome Position

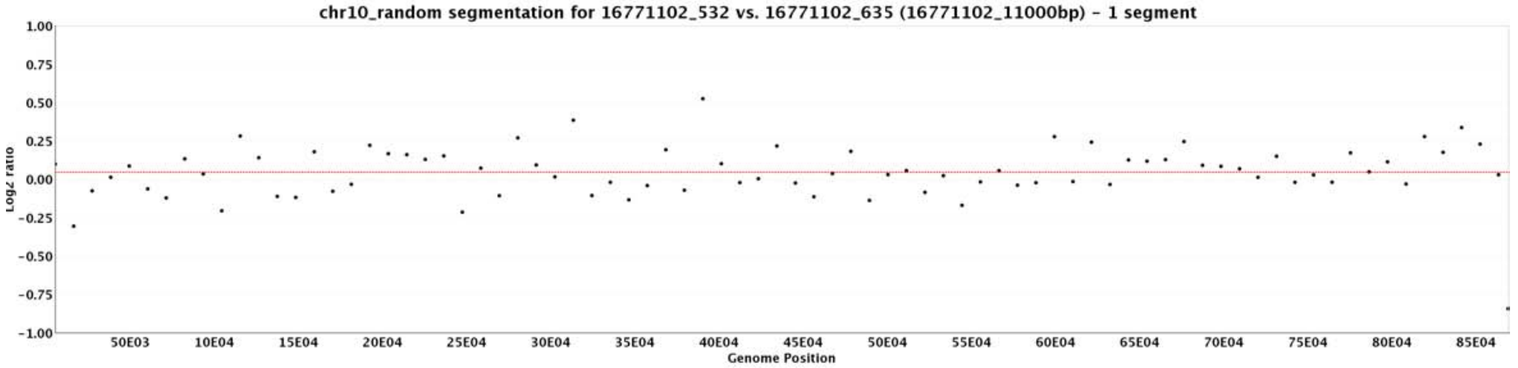
chr10_random segmentation for 16771102_532 vs. 16771102_635 (16771102_11000bp) - 1 segment
Log2 ratio
1.00 0.75 0.50 0.25 0.00 -0.25 -0.50 -0.75 -1.00
50E03 10E04 15E04 20E04 25E04 30E04 35E04 40E04 45E04 50E04 55E04 60E04 65E04 70E04 75E04 80E04 85E04
Genome Position

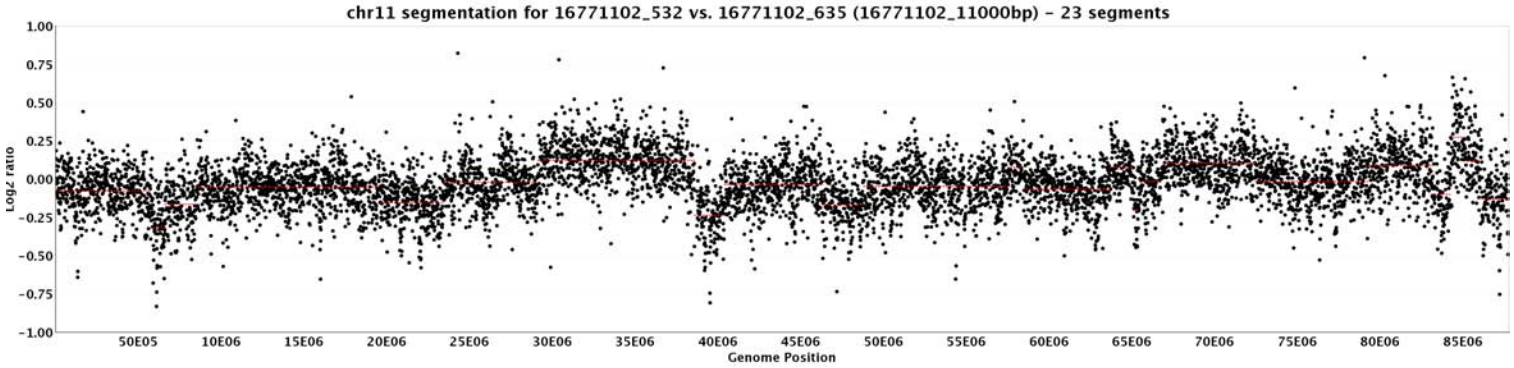
chr11 segmentation for 16771102_532 vs. 16771102_635 (16771102_11000bp) - 23 segments
Log2 ratio
Genome Position

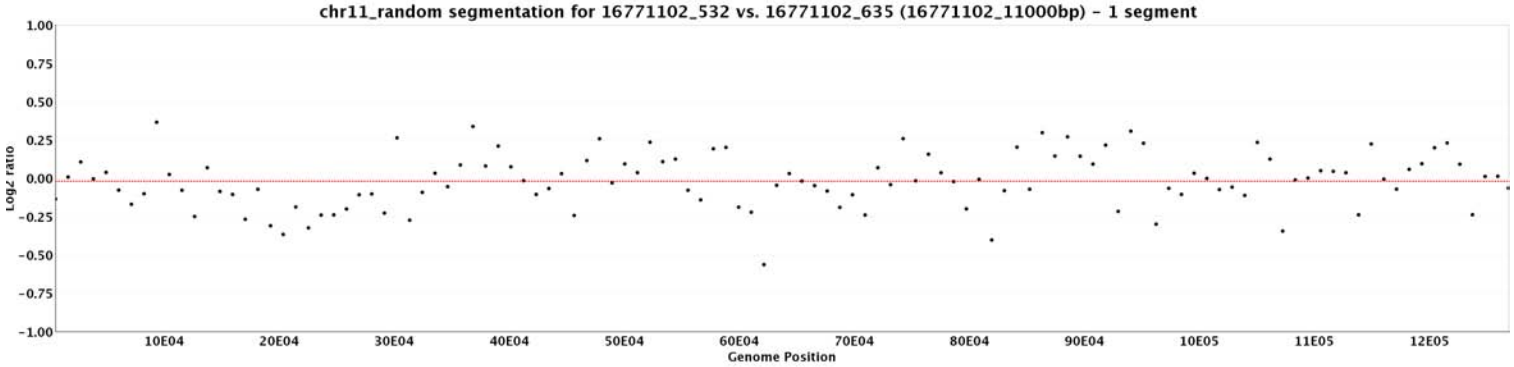
chr11_random segmentation for 16771102_532 vs. 16771102_635 (16771102_11000bp) - 1 segment
Log2 ratio
Genome Position

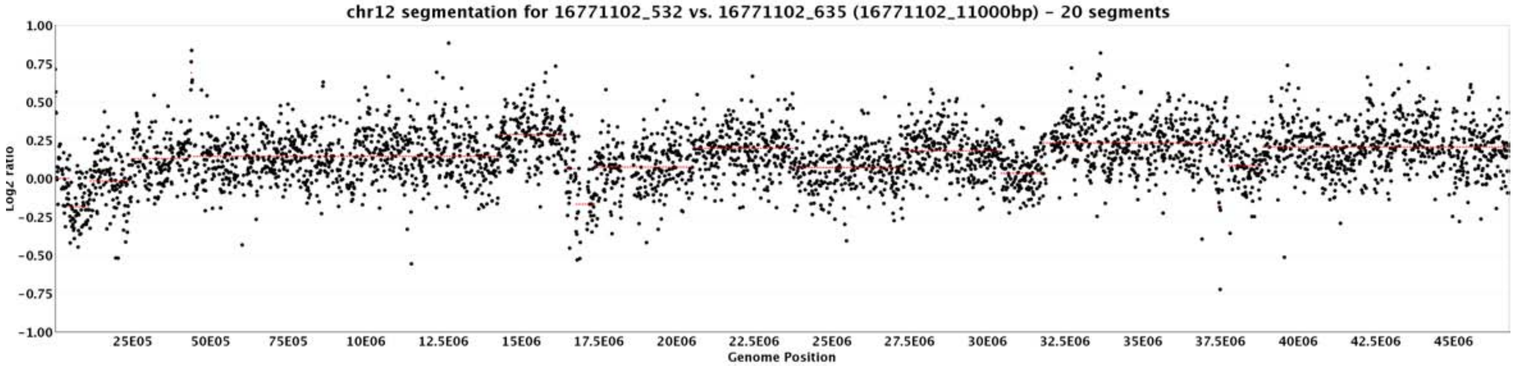
chr12 segmentation for 16771102_532 vs. 16771102_635 (16771102_11000bp) - 20 segments
Log2 ratio
Genome Position

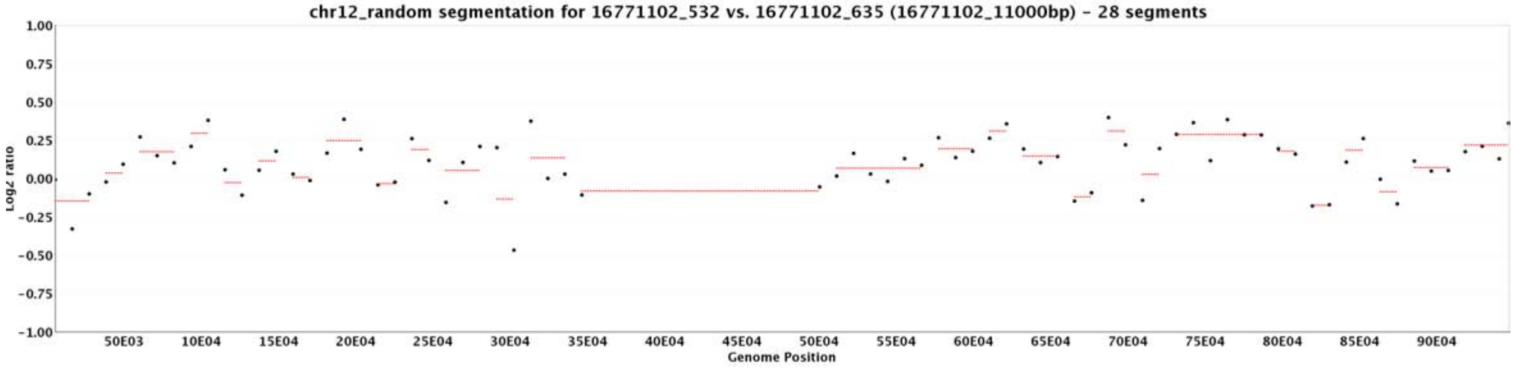
chr12_random segmentation for 16771102_532 vs. 16771102_635 (16771102_11000bp) - 28 segments
Log2 ratio
Genome Position

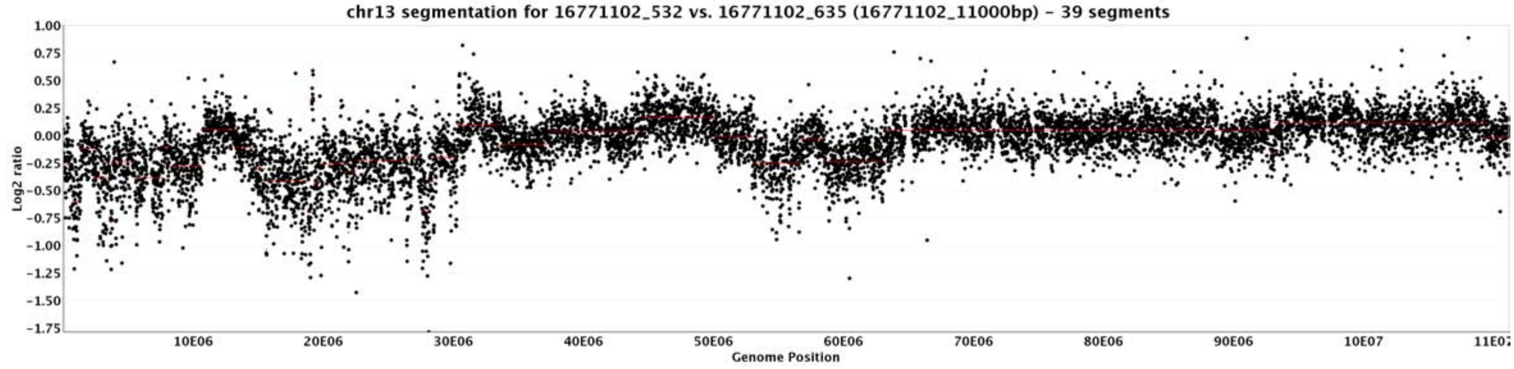
chr13 segmentation for 16771102_532 vs. 16771102_635 (16771102_11000bp) - 39 segments
Log2 ratio
Genome Position

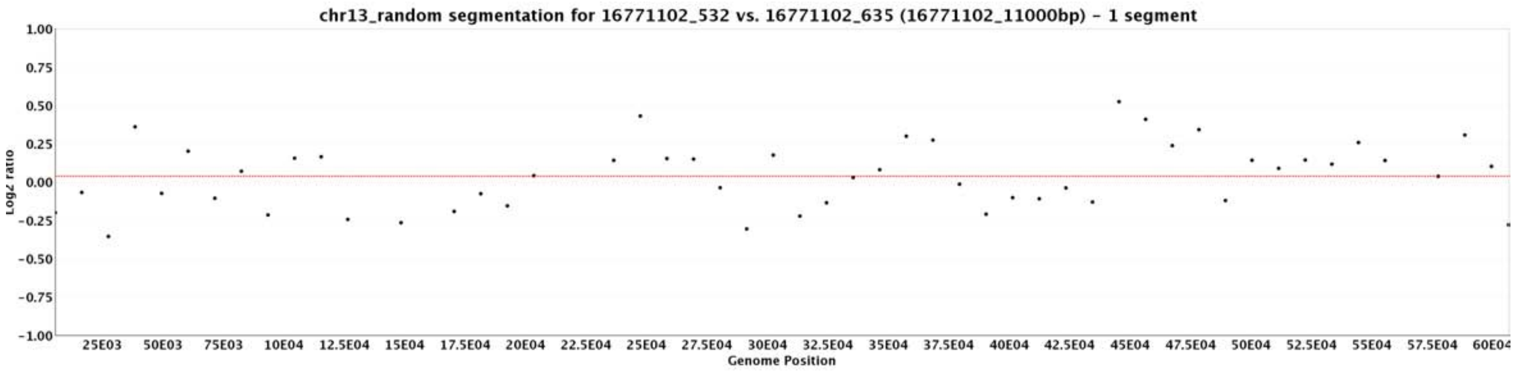
chr13_random segmentation for 16771102_532 vs. 16771102_635 (16771102_11000bp) - 1 segment
Log2 ratio
Genome Position

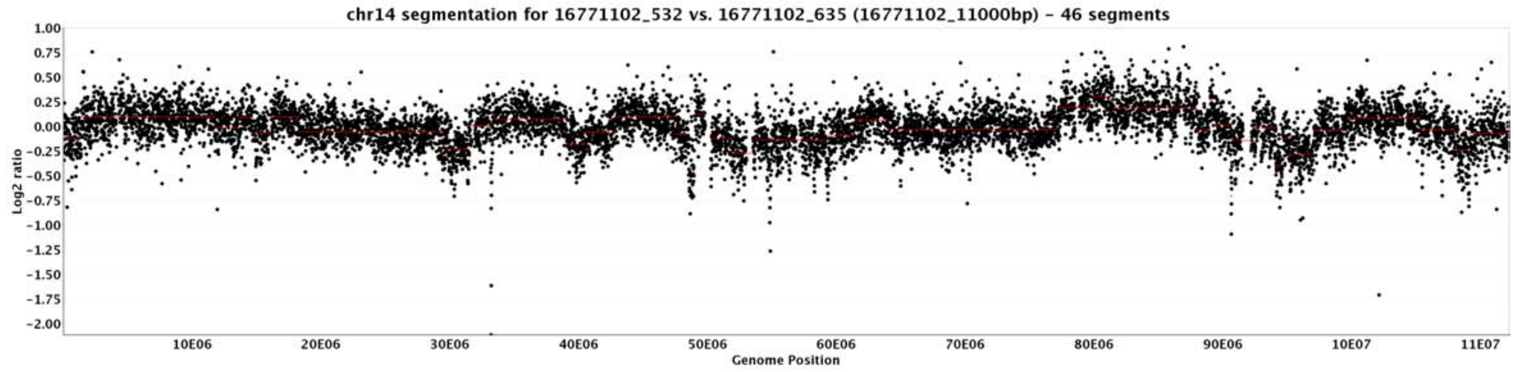
chr14 segmentation for 16771102_532 vs. 16771102_635 (16771102_11000bp) - 46 segments
Log2 ratio
Genome Position

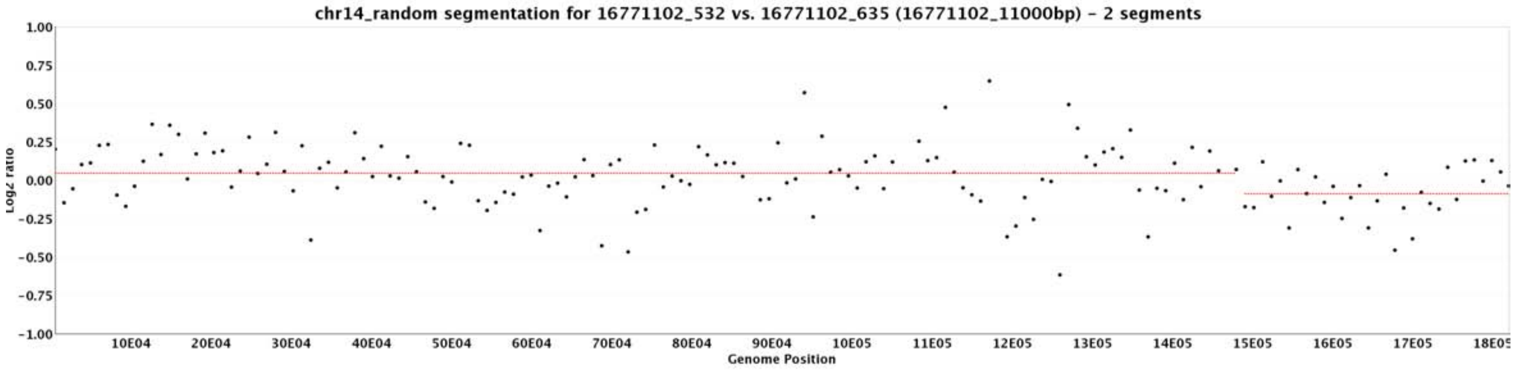
chr14_random segmentation for 16771102_532 vs. 16771102_635 (16771102_11000bp) - 2 segments
Log2 ratio
Genome Position

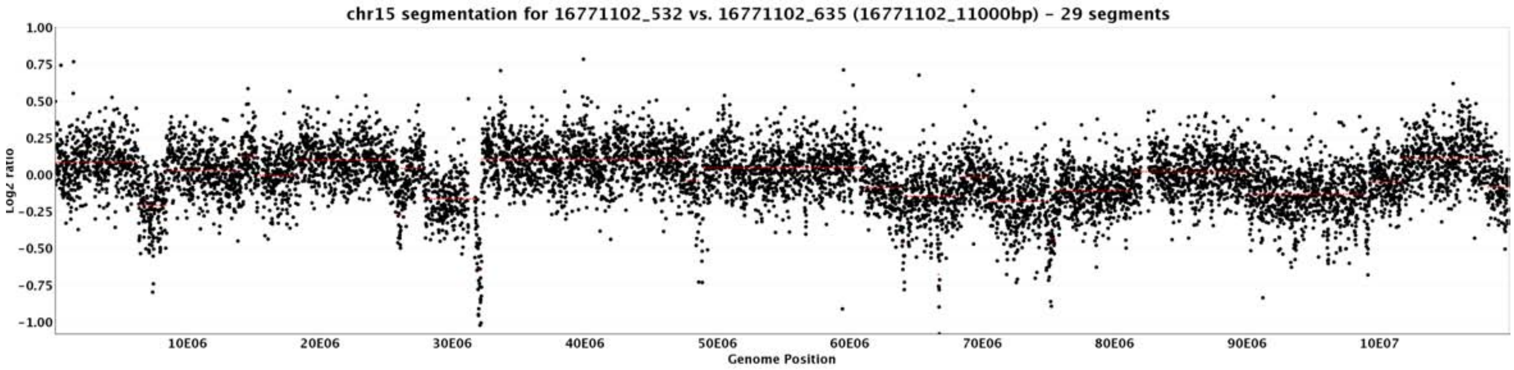
chr15 segmentation for 16771102_532 vs. 16771102_635 (16771102_11000bp) - 29 segments
Log2 ratio
Genome Position

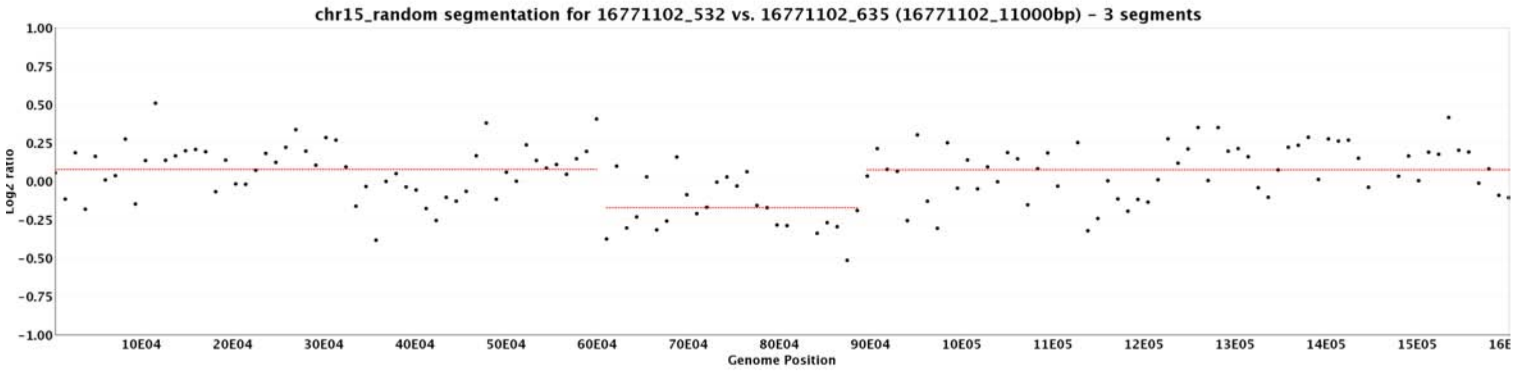
chr15_random segmentation for 16771102_532 vs. 16771102_635 (16771102_11000bp) - 3 segments
Log2 ratio
Genome Position

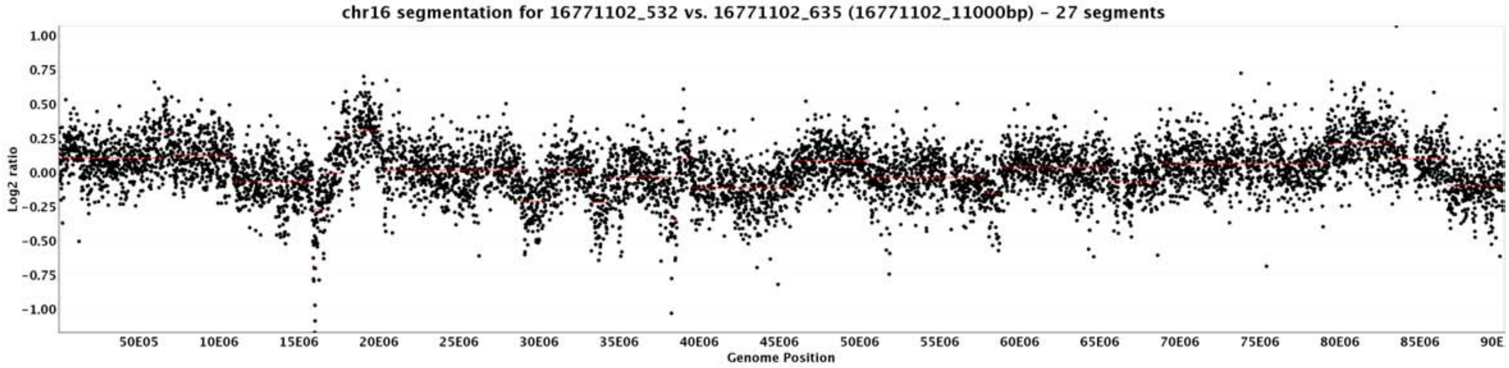
chr16 segmentation for 16771102_532 vs. 16771102_635 (16771102_11000bp) - 27 segments
Log2 ratio
Genome Position

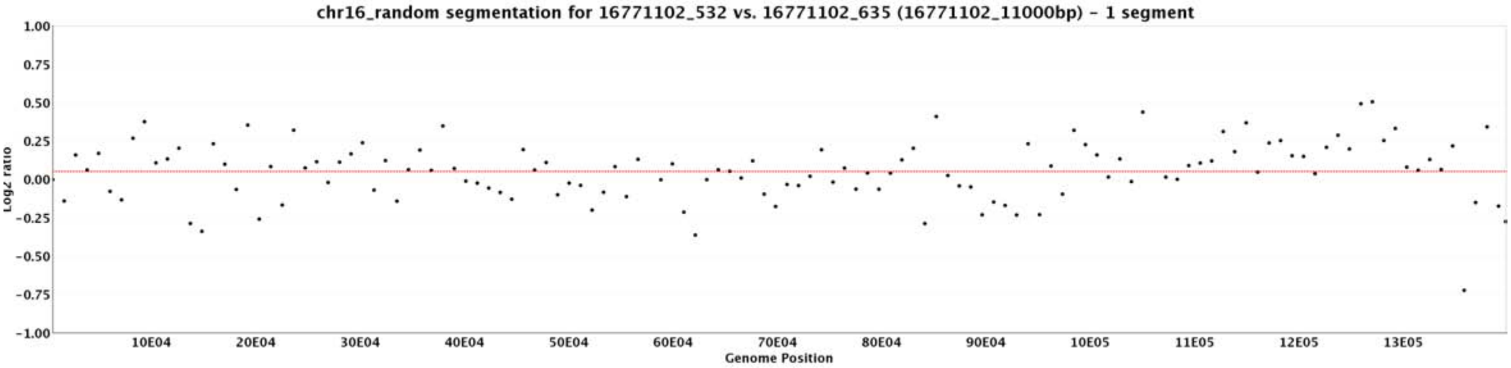
chr16_random segmentation for 16771102_532 vs. 16771102_635 (16771102_11000bp) - 1 segment
Log2 ratio
Genome Position

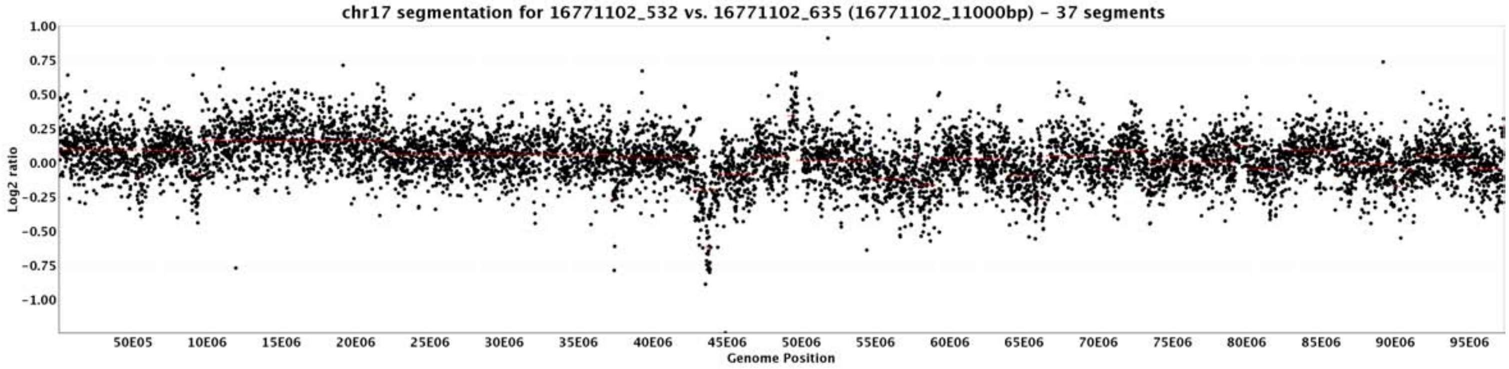
chr17 segmentation for 16771102_532 vs. 16771102_635 (16771102_11000bp) - 37 segments
Log2 ratio
Genome Position

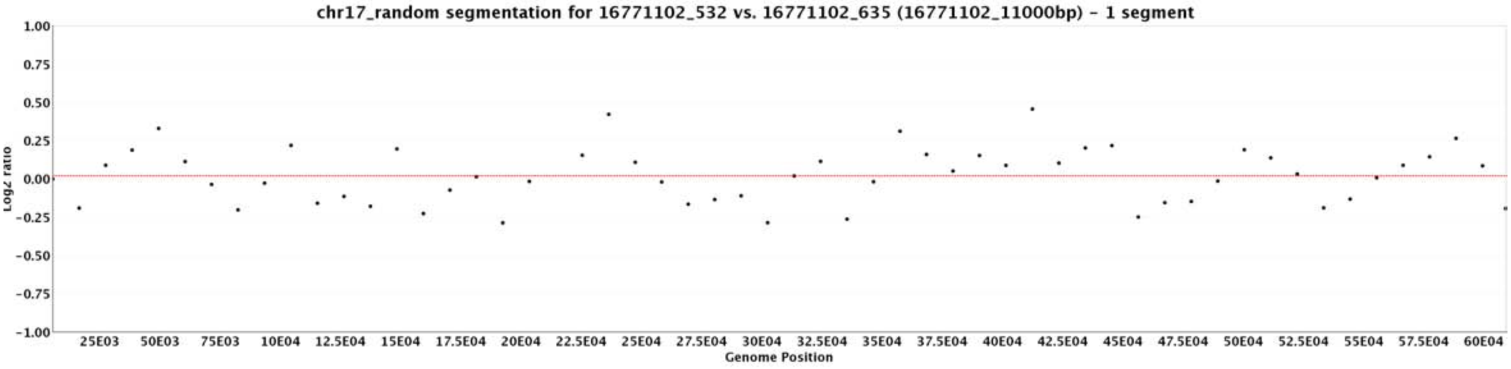
chr17_random segmentation for 16771102_532 vs. 16771102_635 (16771102_11000bp) - 1 segment
Log2 ratio
Genome Position

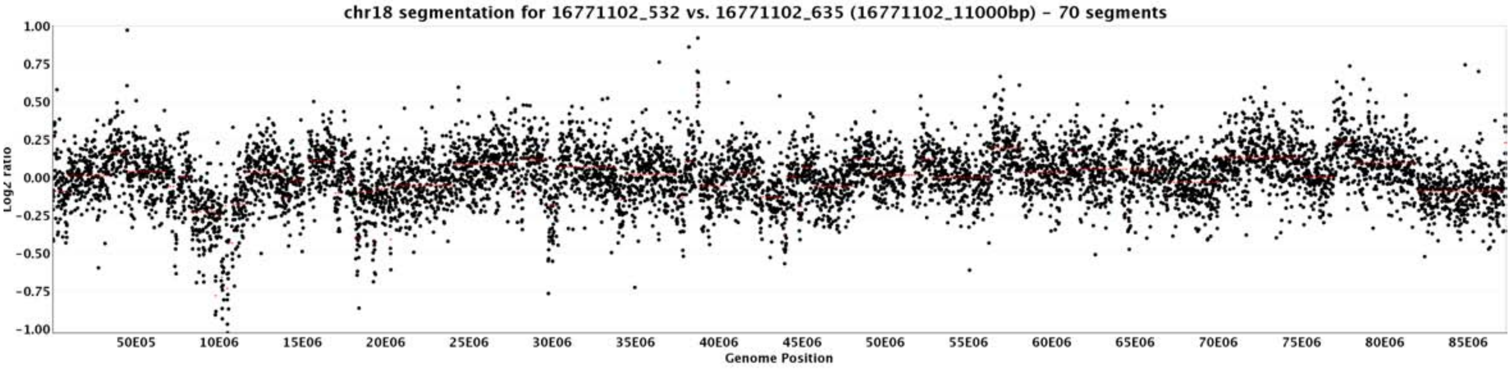
chr18 segmentation for 16771102_532 vs. 16771102_635 (16771102_11000bp) - 70 segments
Log2 ratio
Genome Position

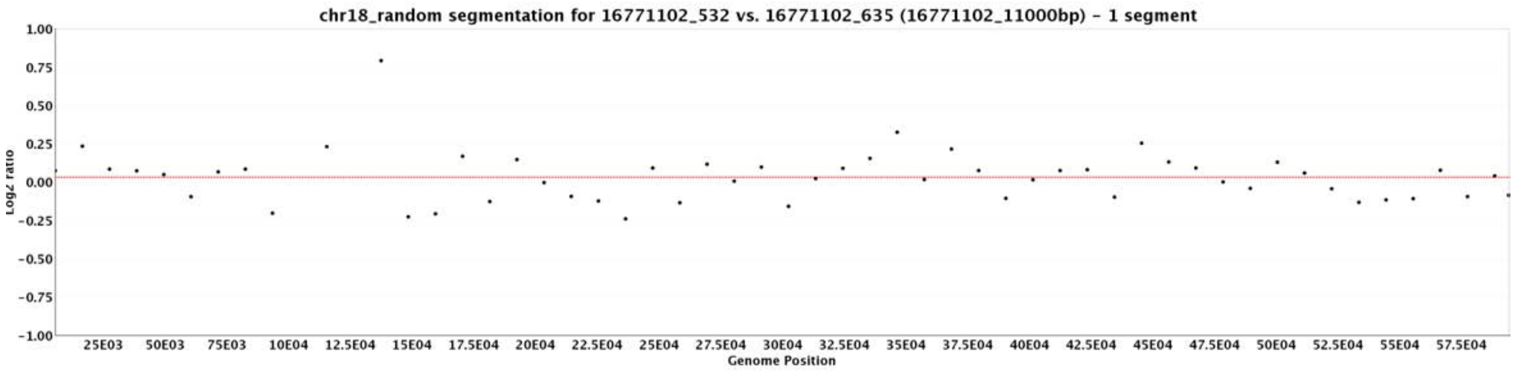
chr18_random segmentation for 16771102_532 vs. 16771102_635 (16771102_11000bp) - 1 segment
Log2 ratio
Genome Position

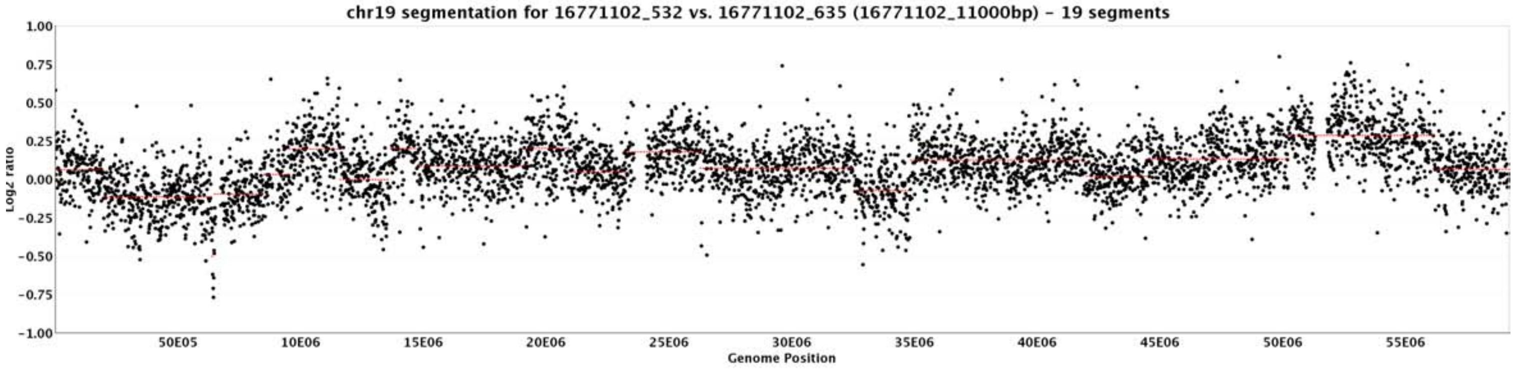
chr19 segmentation for 16771102_532 vs. 16771102_635 (16771102_11000bp) - 19 segments
Log2 ratio
Genome Position

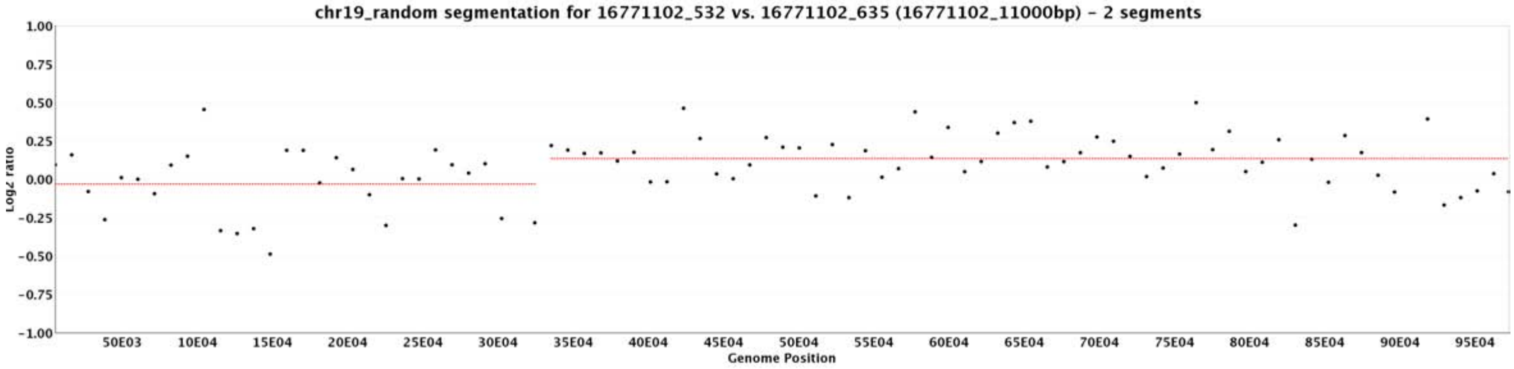
chr19_random segmentation for 16771102_532 vs. 16771102_635 (16771102_11000bp) - 2 segments
Log2 ratio
Genome Position

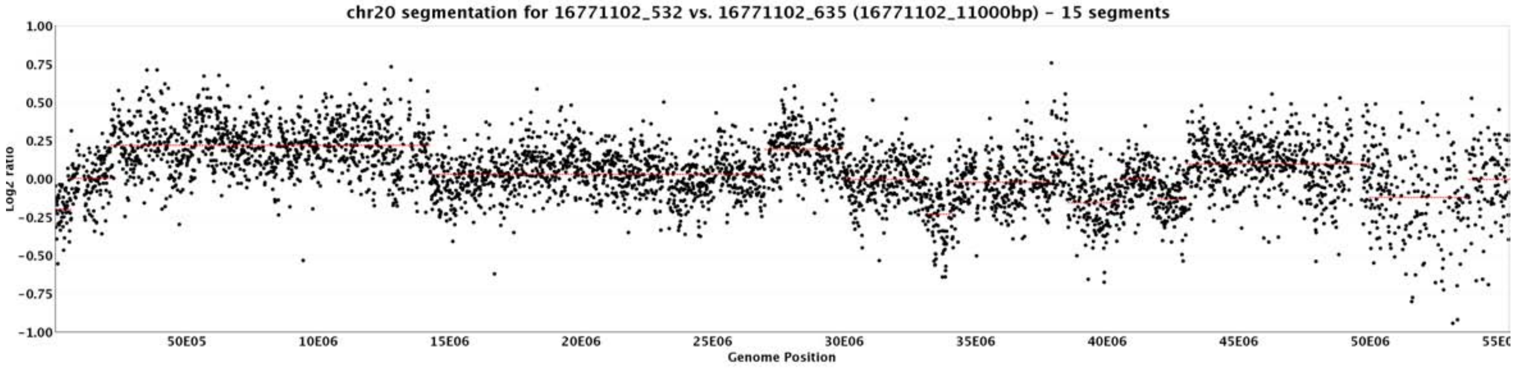
chr20 segmentation for 16771102_532 vs. 16771102_635 (16771102_11000bp) - 15 segments
Log2 ratio
Genome Position

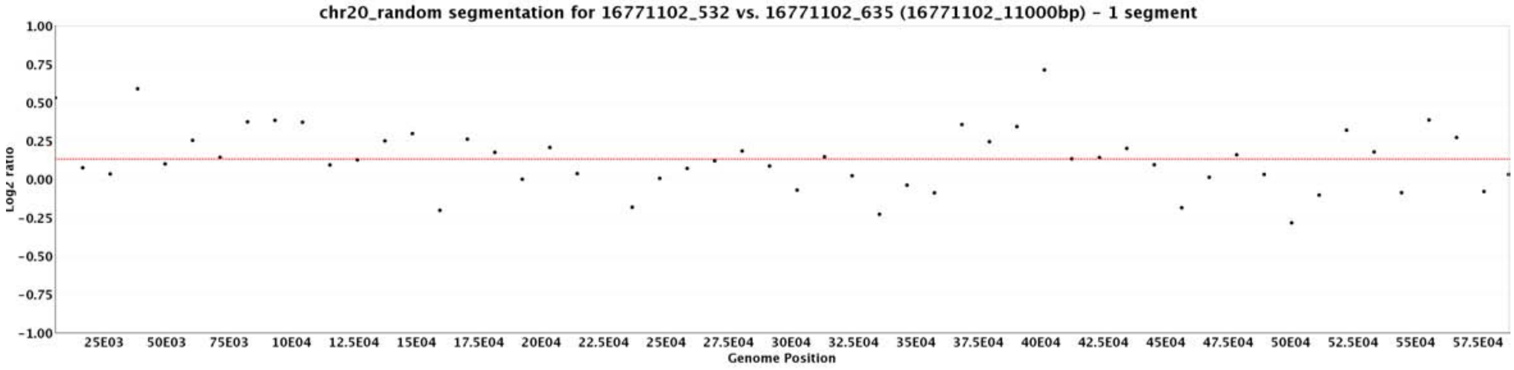
chr20_random segmentation for 16771102_532 vs. 16771102_635 (16771102_11000bp) - 1 segment
Log2 ratio
Genome Position

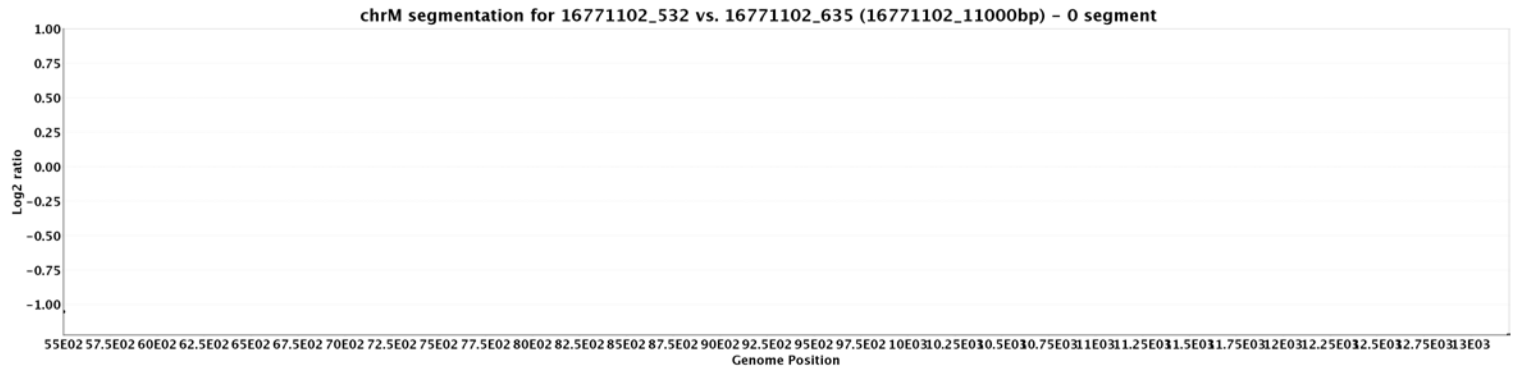
chrM segmentation for 16771102_532 vs. 16771102_635 (16771102_11000bp) - 0 segment
Log2 ratio
Genome Position

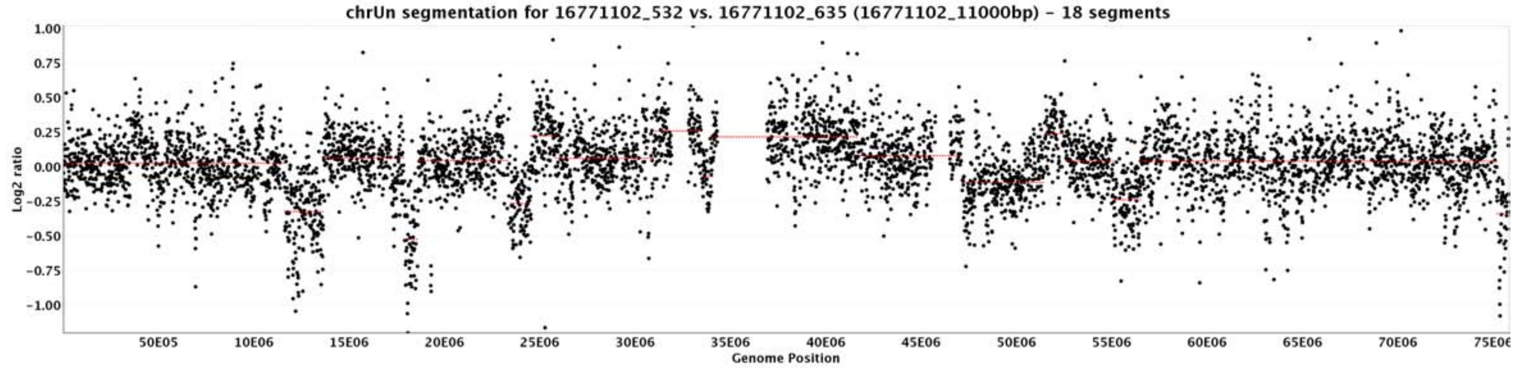
chrUn segmentation for 16771102_532 vs. 16771102_635 (16771102_11000bp) - 18 segments
Log2 ratio
Genome Position

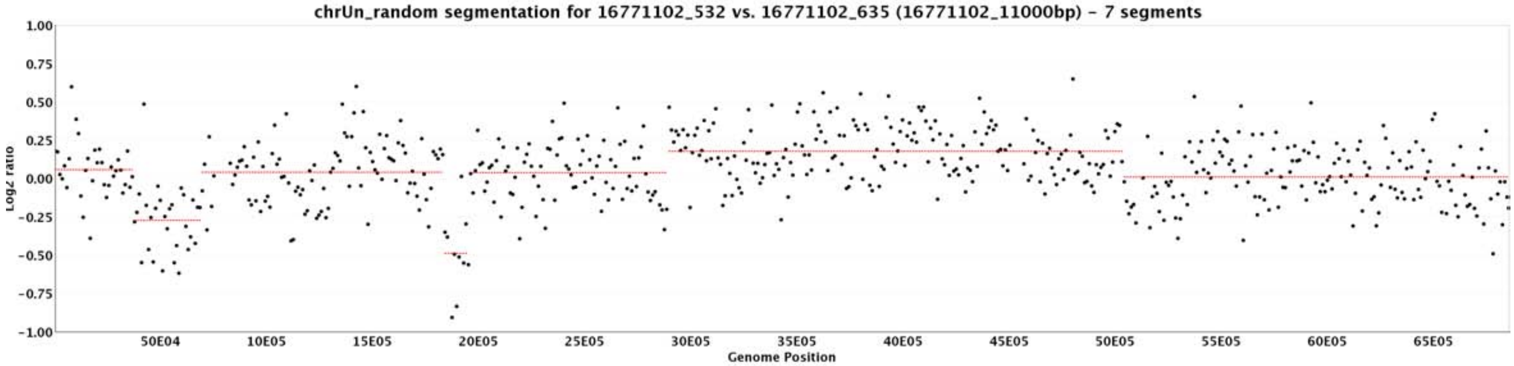
chrUn_random segmentation for 16771102_532 vs. 16771102_635 (16771102_11000bp) - 7 segments
Log2 ratio
Genome Position

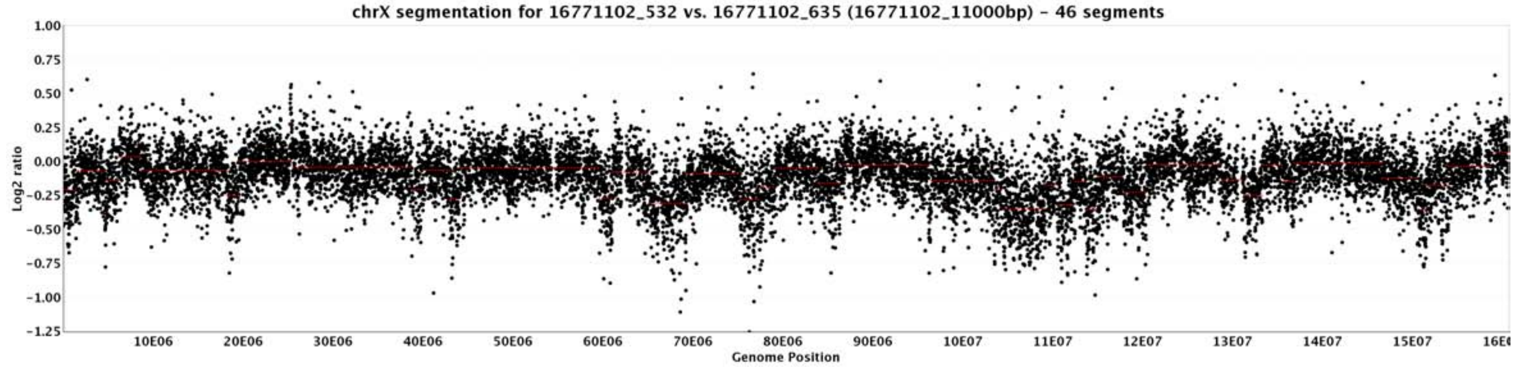
chrX segmentation for 16771102_532 vs. 16771102_635 (16771102_11000bp) - 46 segments
Log2 ratio
Genome Position

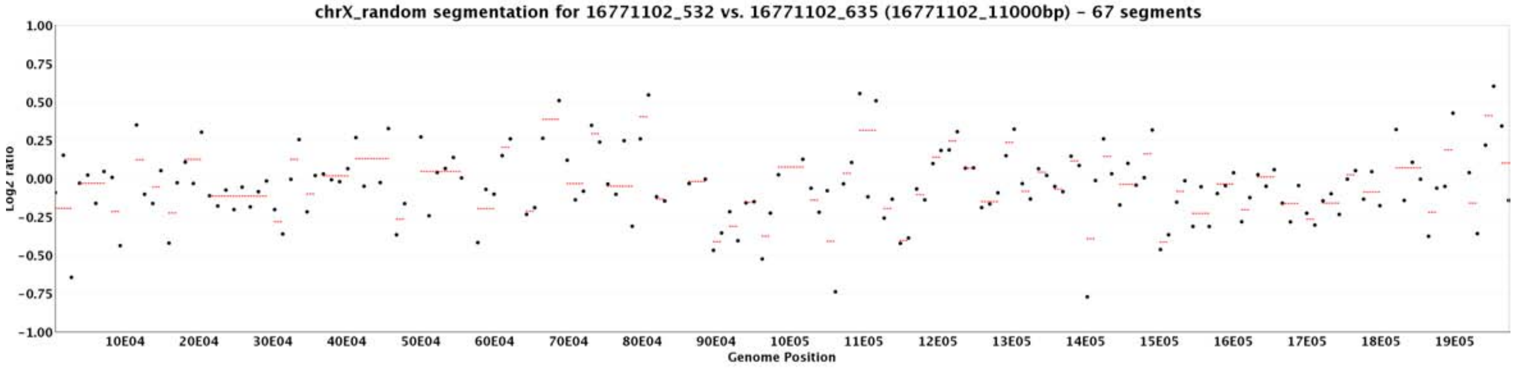
chrX_random segmentation for 16771102_532 vs. 16771102_635 (16771102_11000bp) - 67 segments
Log2 ratio
Genome Position

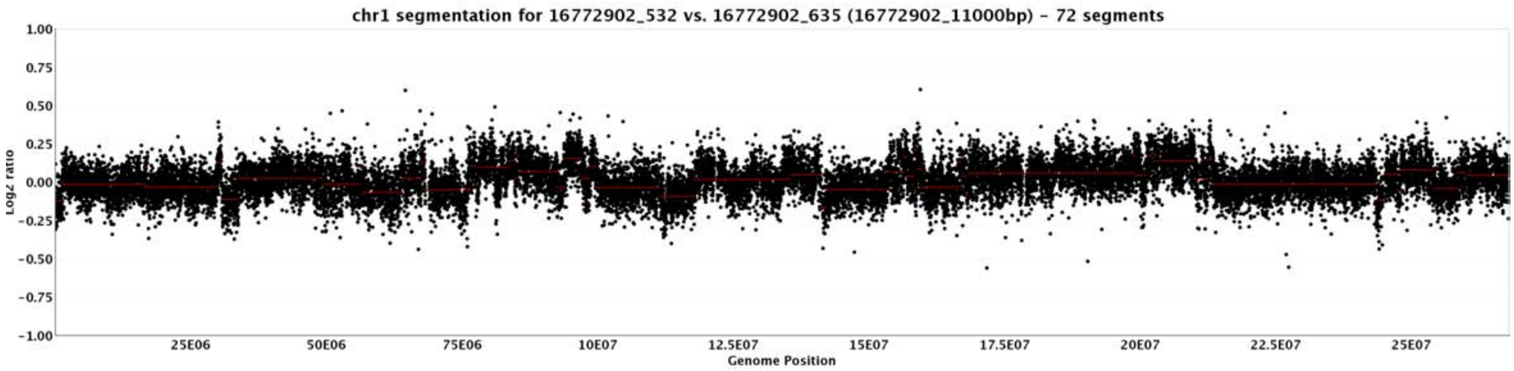
chr1 segmentation for 16772902_532 vs. 16772902_635 (16772902_11000bp) - 72 segments
Log2 ratio
1.00
0.75
0.50
0.25
0.00
-0.25
-0.50
-0.75
-1.00
25E06
50E06
75E06
10E07
12.5E07
15E07
17.5E07
20E07
22.5E07
25E07
Genome Position

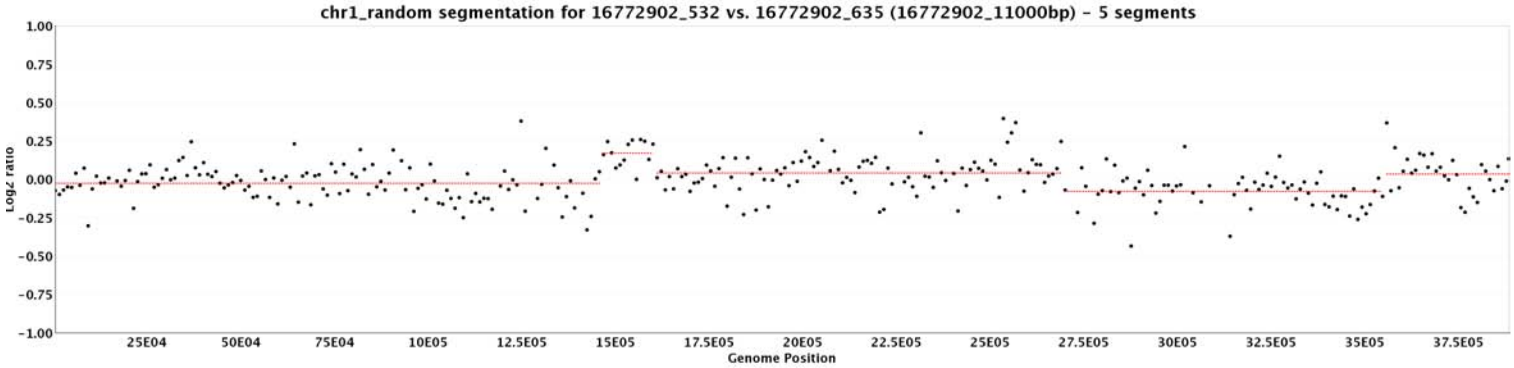
chr1_random segmentation for 16772902_532 vs. 16772902_635 (16772902_11000bp) - 5 segments
Log2 ratio
1.00
0.75
0.50
0.25
0.00
-0.25
-0.50
-0.75
-1.00
25E04
50E04
75E04
10E05
12.5E05
15E05
17.5E05
20E05
22.5E05
25E05
27.5E05
30E05
32.5E05
35E05
37.5E05
Genome Position

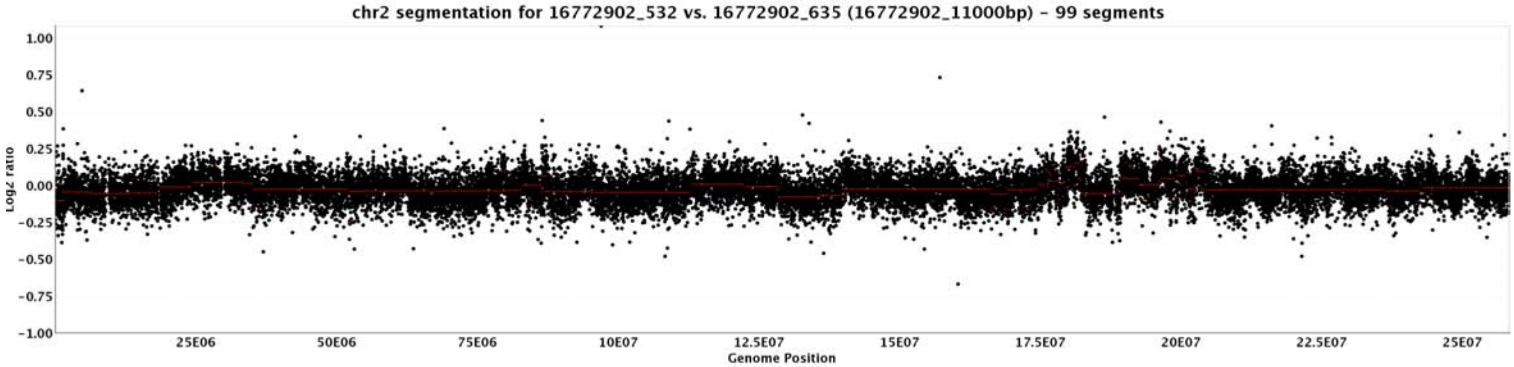
chr2 segmentation for 16772902_532 vs. 16772902_635 (16772902_11000bp) - 99 segments
Log2 ratio
1.00
0.75
0.50
0.25
0.00
-0.25
-0.50
-0.75
-1.00
25E06
50E06
75E06
10E07
12.5E07
15E07
17.5E07
20E07
22.5E07
25E07
Genome Position

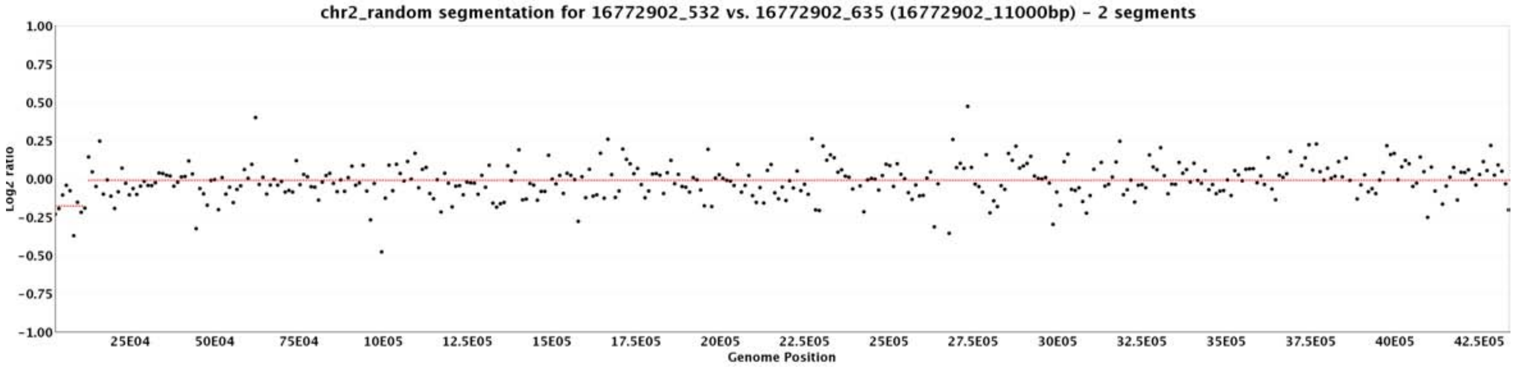
chr2_random segmentation for 16772902_532 vs. 16772902_635 (16772902_11000bp) - 2 segments
Log2 ratio
1.00
0.75
0.50
0.25
0.00
-0.25
-0.50
-0.75
-1.00
25E04
50E04
75E04
10E05
12.5E05
15E05
17.5E05
20E05
22.5E05
25E05
27.5E05
30E05
32.5E05
35E05
37.5E05
40E05
42.5E05
Genome Position

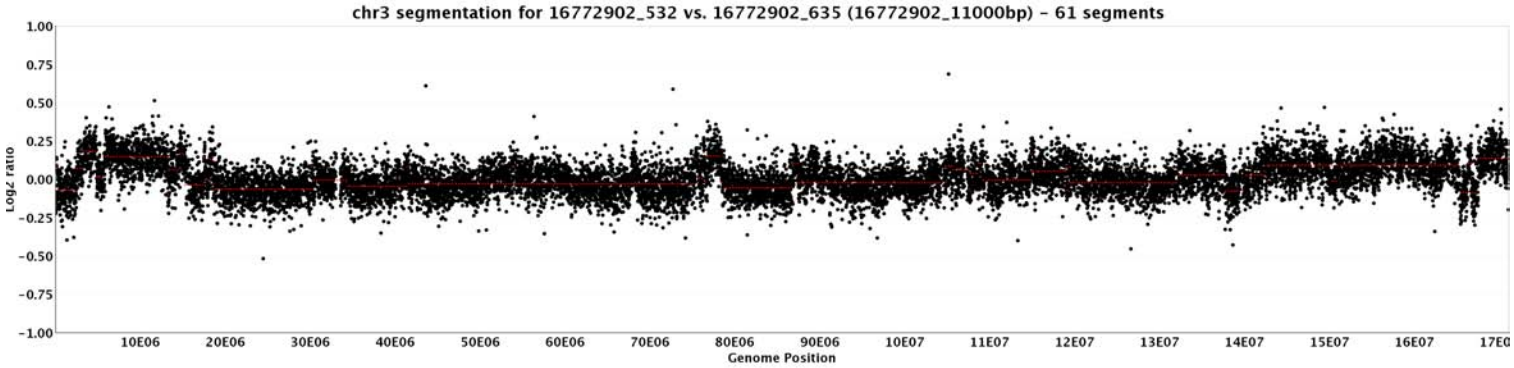
chr3 segmentation for 16772902_532 vs. 16772902_635 (16772902_11000bp) - 61 segments
Log2 ratio
1.00
0.75
0.50
0.25
0.00
-0.25
-0.50
-0.75
-1.00
10E06
20E06
30E06
40E06
50E06
60E06
70E06
80E06
90E06
10E07
11E07
12E07
13E07
14E07
15E07
16E07
17E0
Genome Position

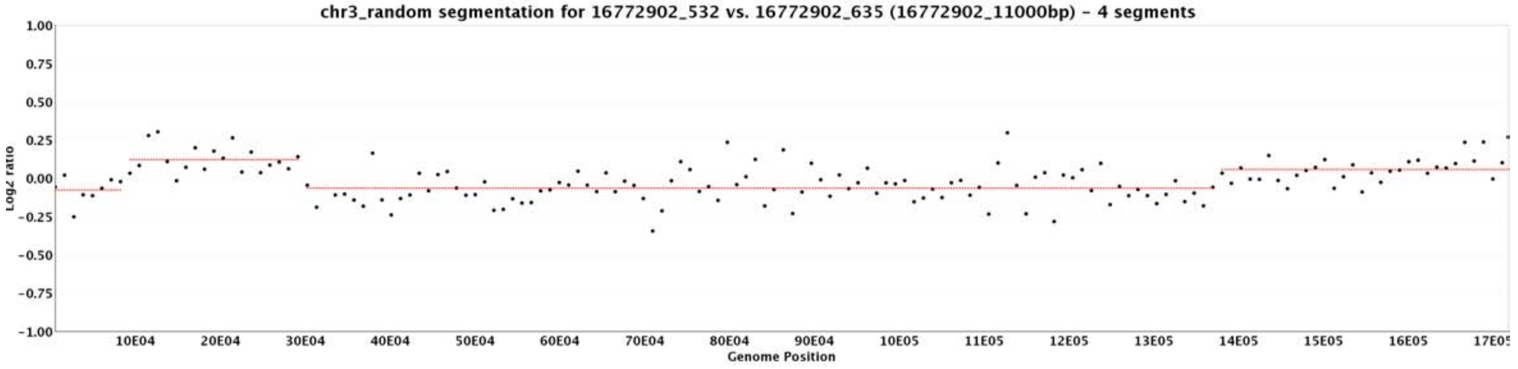
chr3_random segmentation for 16772902_532 vs. 16772902_635 (16772902_11000bp) - 4 segments
Log2 ratio
Genome Position

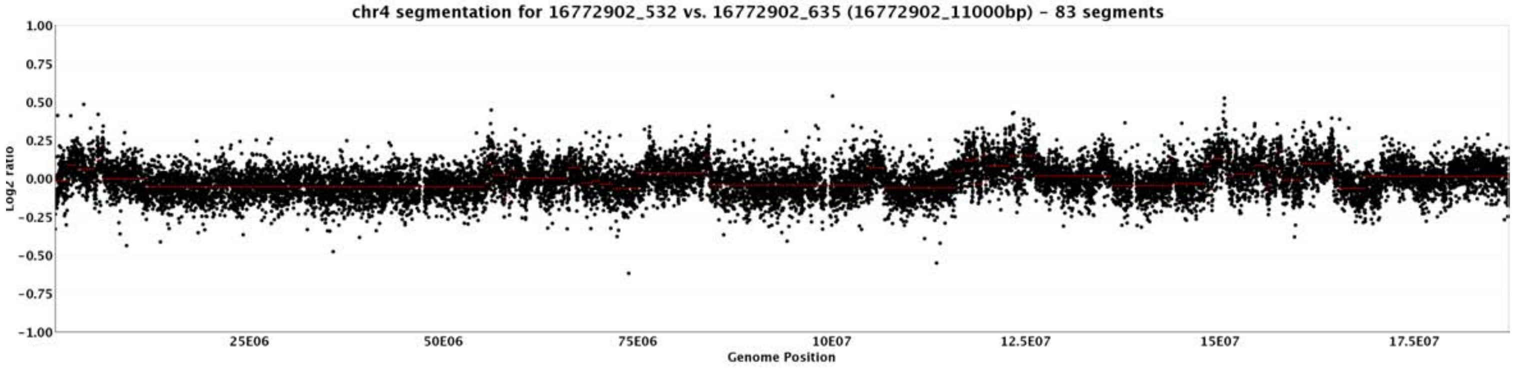
chr4 segmentation for 16772902_532 vs. 16772902_635 (16772902_11000bp) - 83 segments
Log2 ratio
Genome Position

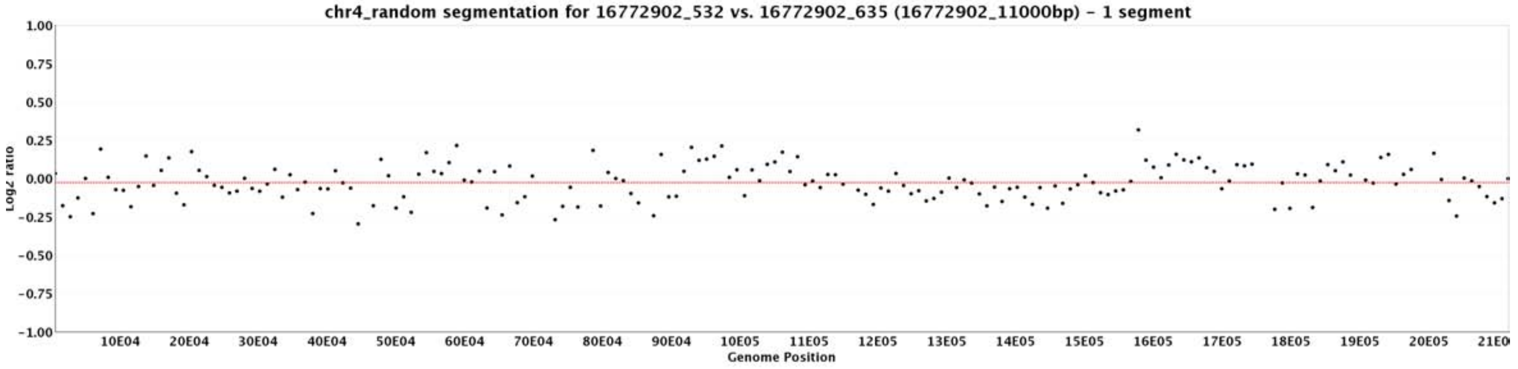
chr4_random segmentation for 16772902_532 vs. 16772902_635 (16772902_11000bp) - 1 segment
Log2 ratio
Genome Position

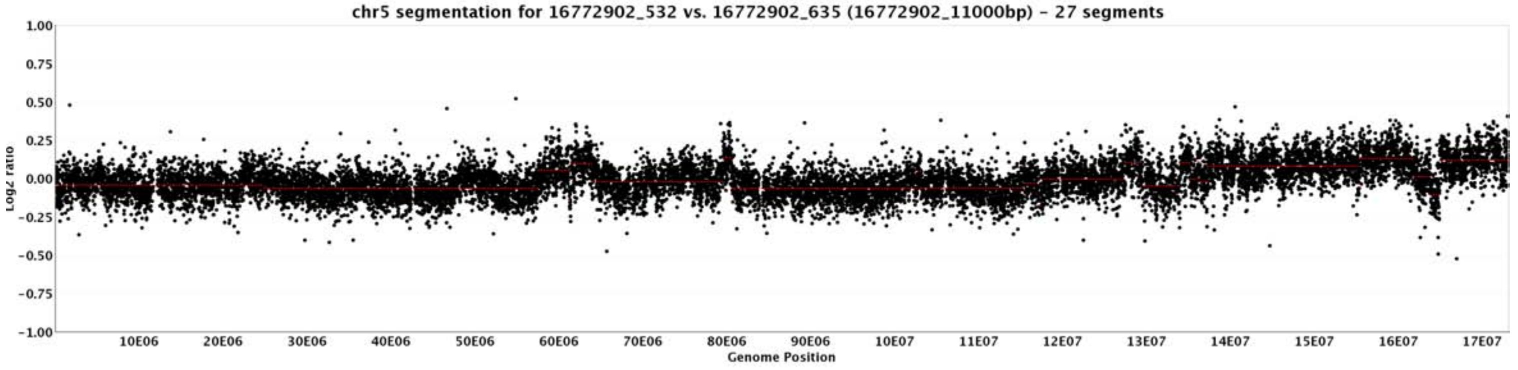
chr5 segmentation for 16772902_532 vs. 16772902_635 (16772902_11000bp) - 27 segments
Log2 ratio
Genome Position

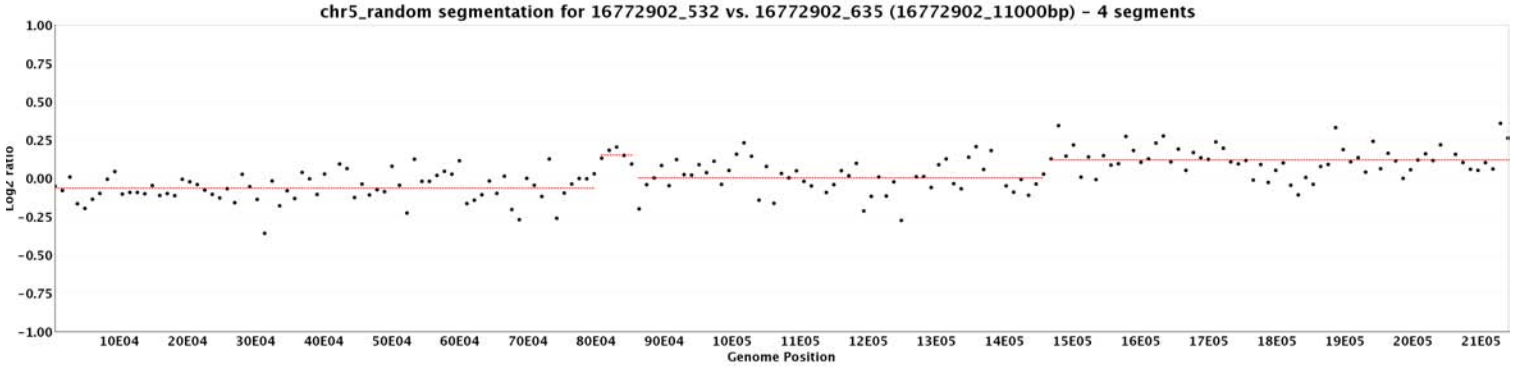
chr5_random segmentation for 16772902_532 vs. 16772902_635 (16772902_11000bp) - 4 segments
Log2 ratio
Genome Position

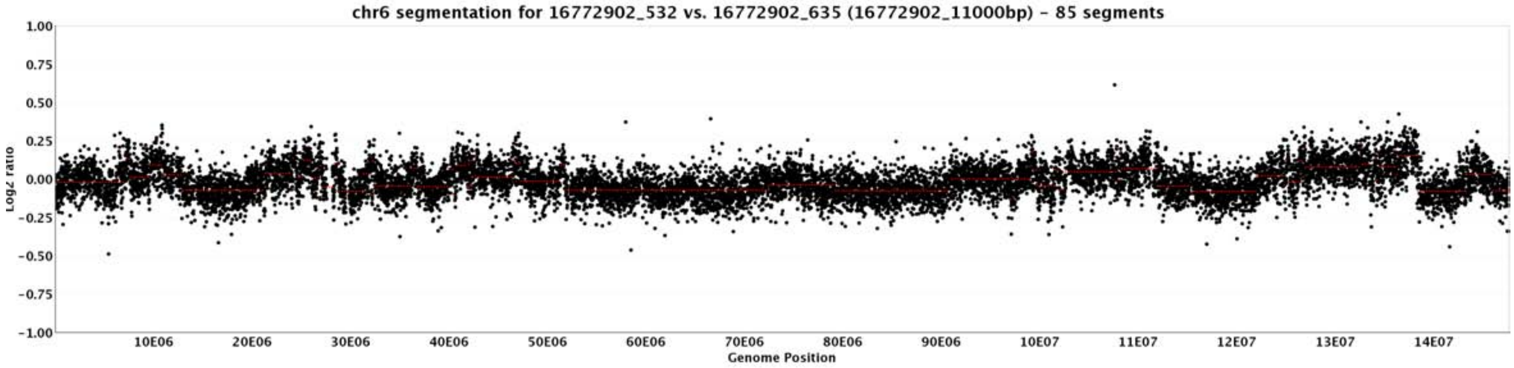
chr6 segmentation for 16772902_532 vs. 16772902_635 (16772902_11000bp) - 85 segments
Log2 ratio
Genome Position

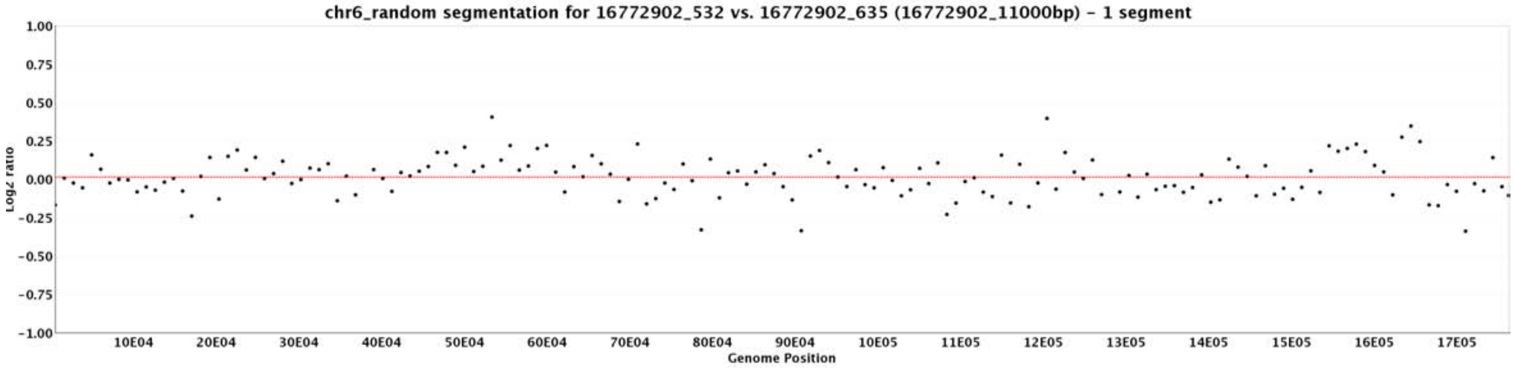
chr6_random segmentation for 16772902_532 vs. 16772902_635 (16772902_11000bp) - 1 segment
Log2 ratio
Genome Position

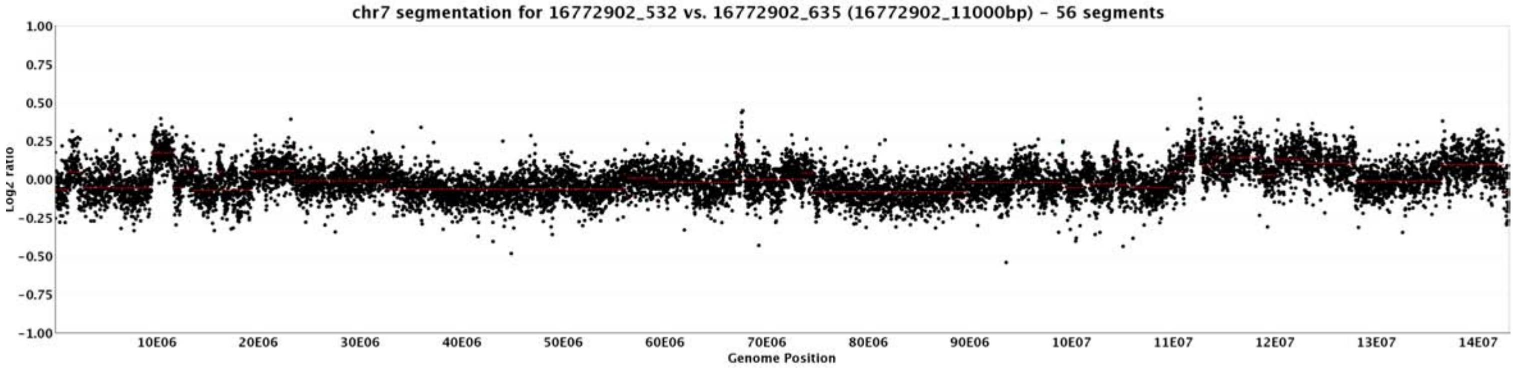
chr7 segmentation for 16772902_532 vs. 16772902_635 (16772902_11000bp) - 56 segments
Log2 ratio
Genome Position

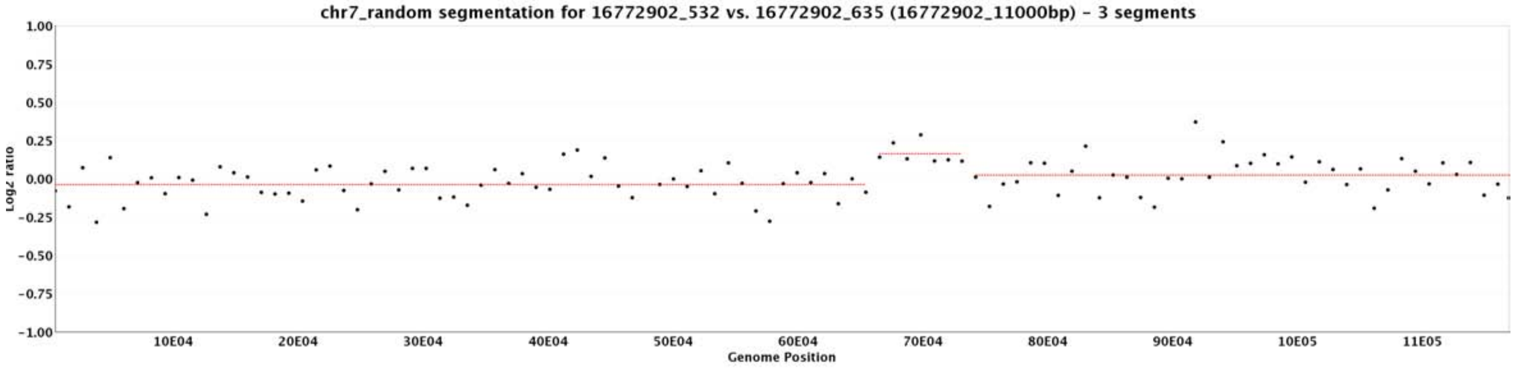
chr7_random segmentation for 16772902_532 vs. 16772902_635 (16772902_11000bp) - 3 segments
Log2 ratio
Genome Position

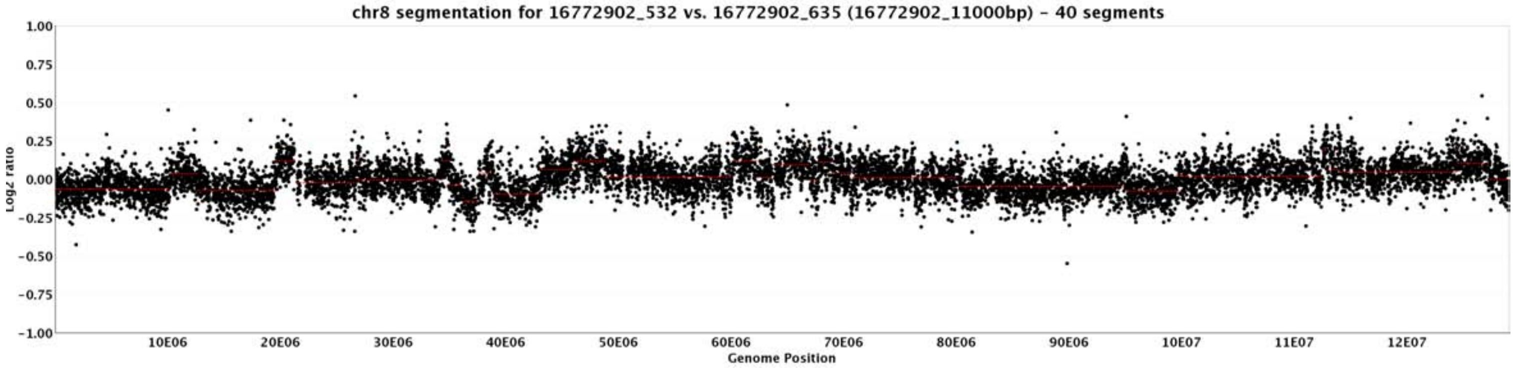
chr8 segmentation for 16772902_532 vs. 16772902_635 (16772902_11000bp) - 40 segments
Log2 ratio
Genome Position

chr8_random segmentation for 16772902_532 vs. 16772902_635 (16772902_11000bp) - 1 segment

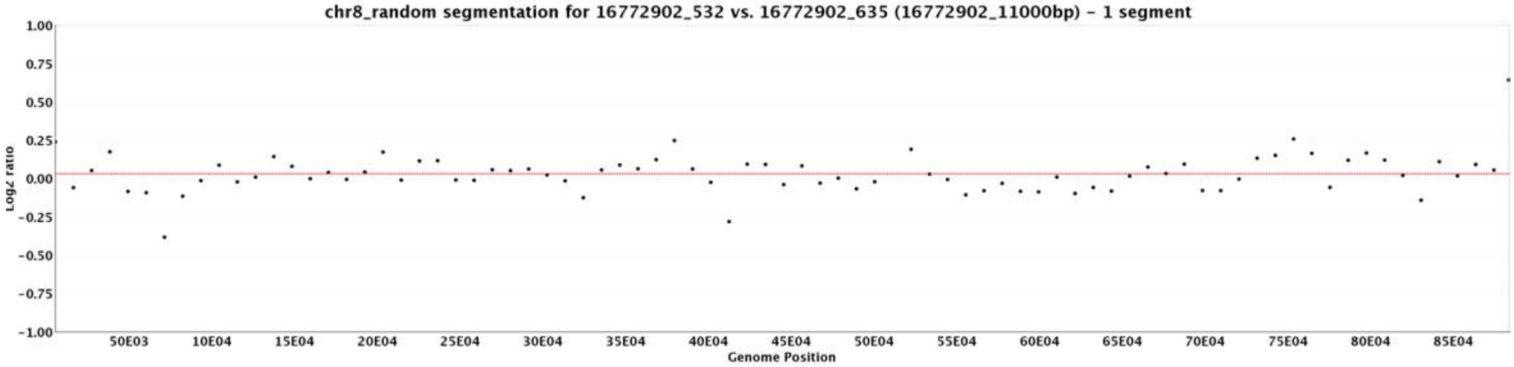

chr9 segmentation for 16772902_532 vs. 16772902_635 (16772902_11000bp) - 69 segments

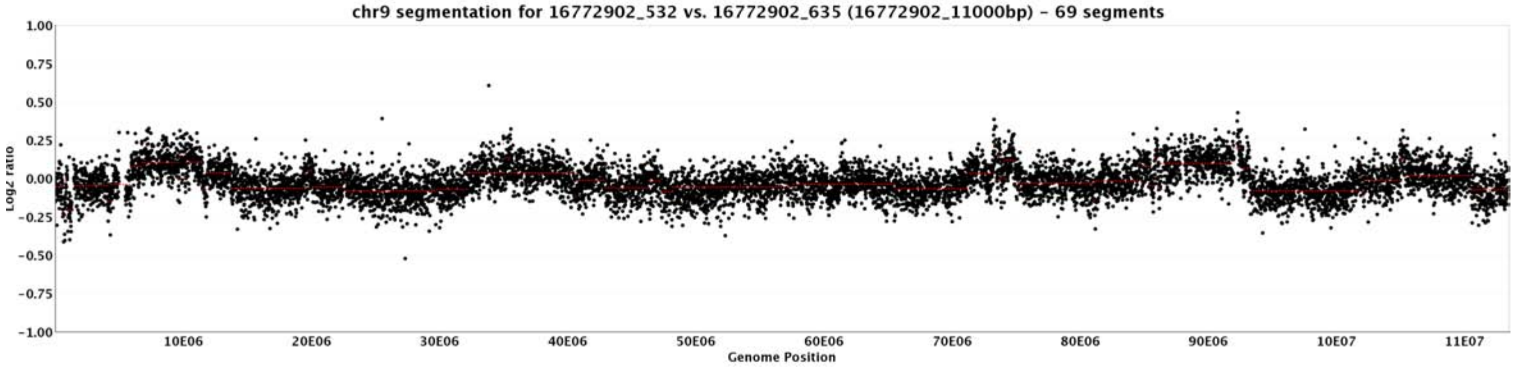

chr9_random segmentation for 16772902_532 vs. 16772902_635 (16772902_11000bp) - 2 segments

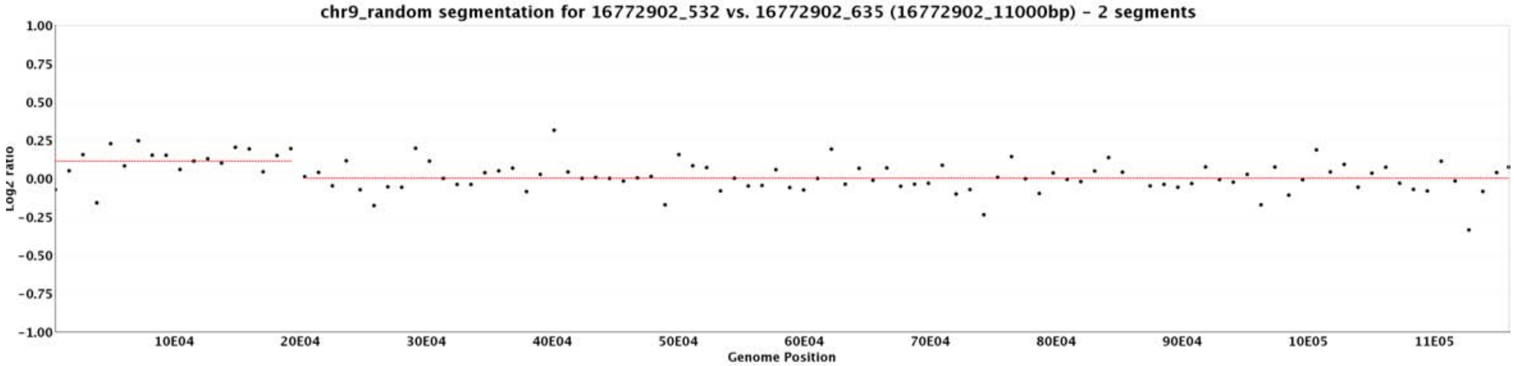

chr10 segmentation for 16772902_532 vs. 16772902_635 (16772902_11000bp) - 69 segments

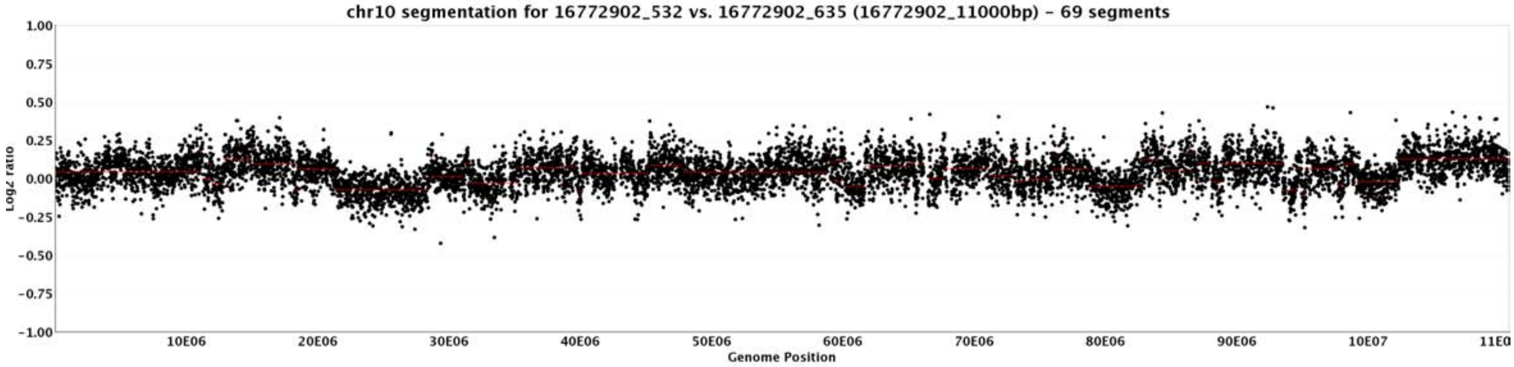

chr10_random segmentation for 16772902_532 vs. 16772902_635 (16772902_11000bp) - 1 segment

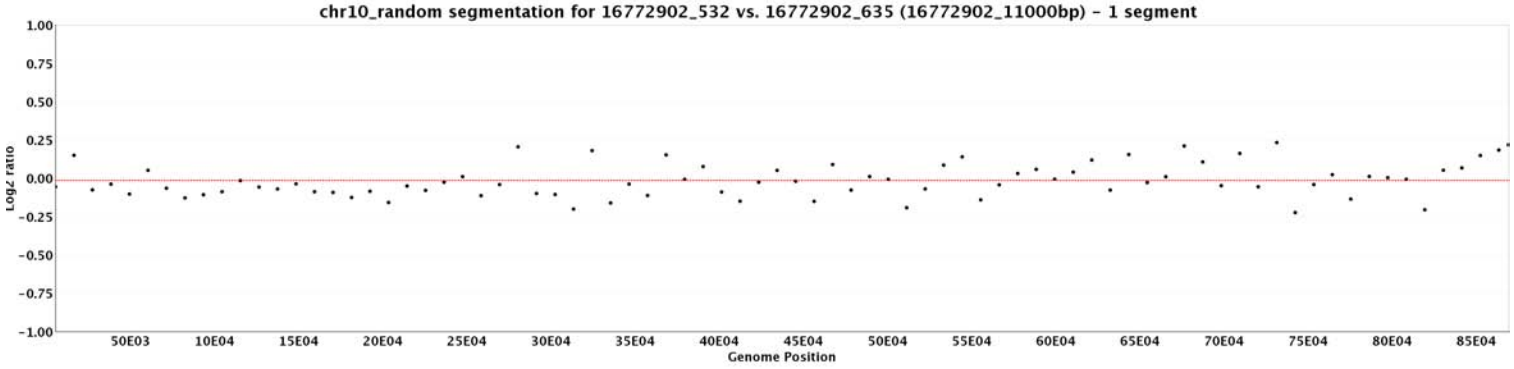

chr11 segmentation for 16772902_532 vs. 16772902_635 (16772902_11000bp) - 38 segments

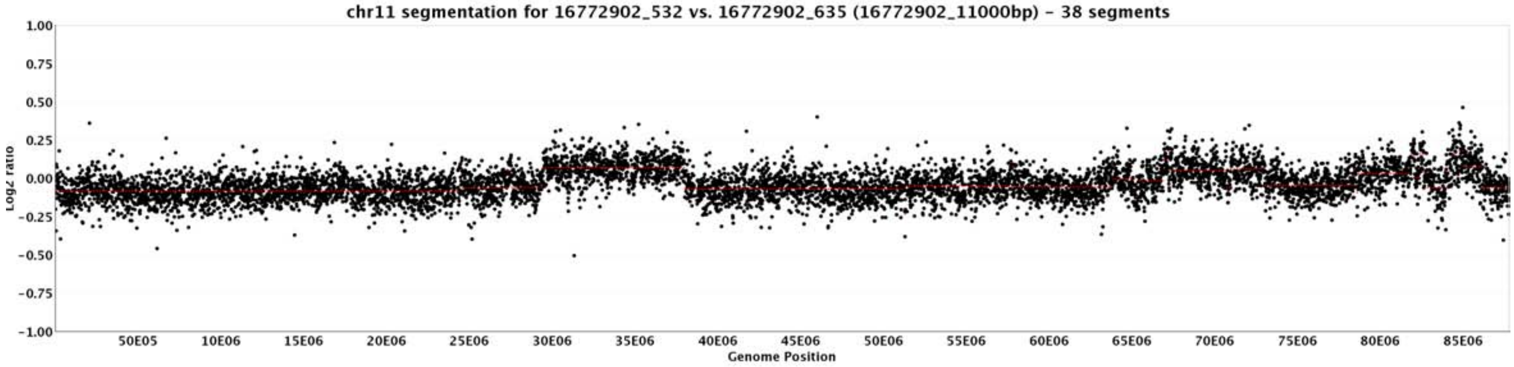

chr11_random segmentation for 16772902_532 vs. 16772902_635 (16772902_11000bp) - 2 segments

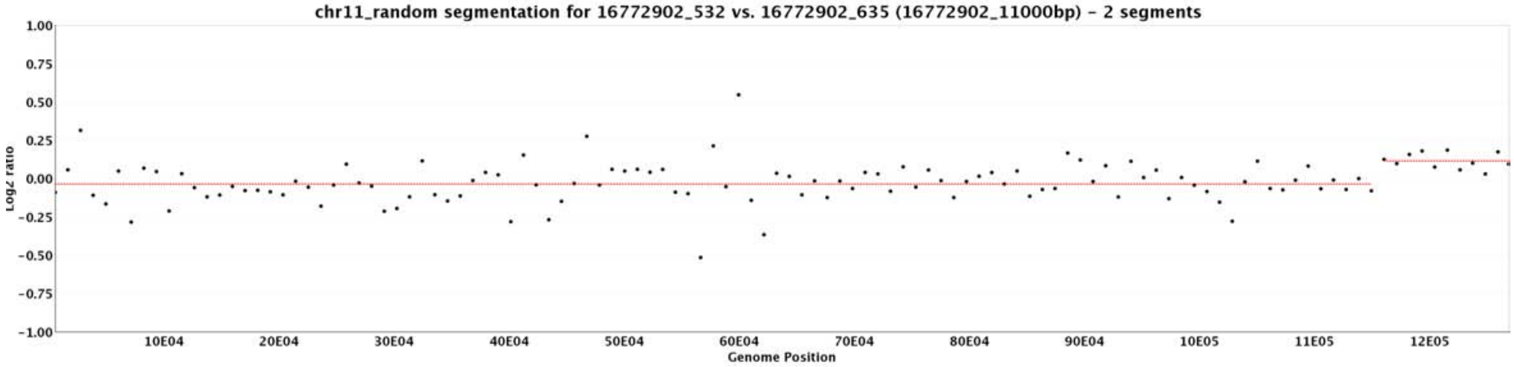

chr12 segmentation for 16772902_532 vs. 16772902_635 (16772902_11000bp) - 44 segments

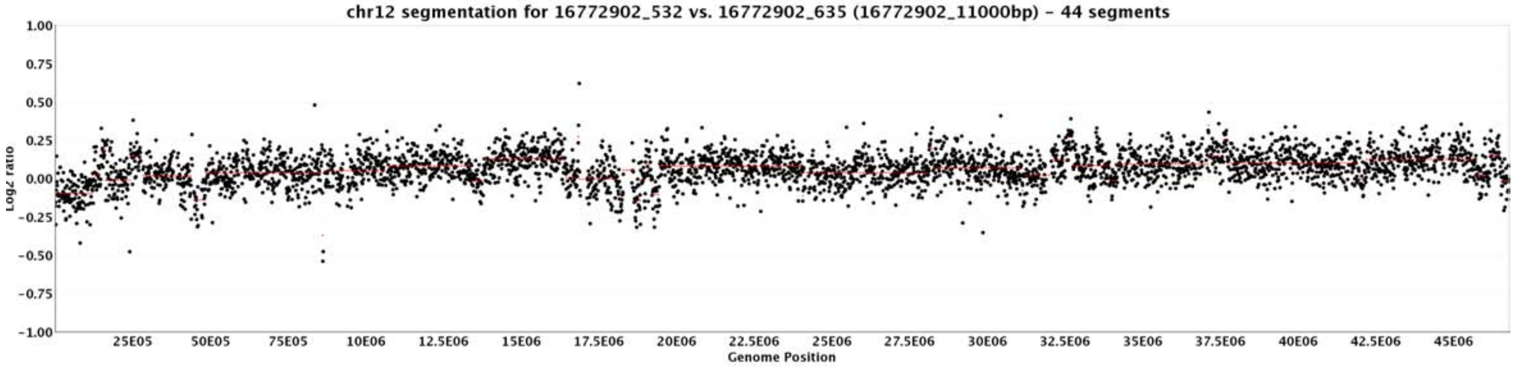

chr12_random segmentation for 16772902_532 vs. 16772902_635 (16772902_11000bp) - 1 segment

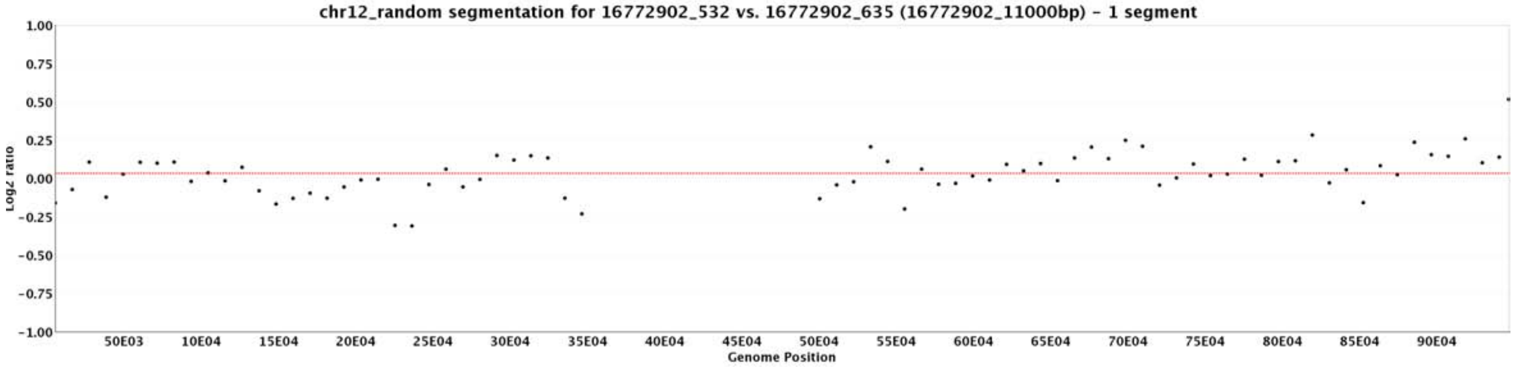

chr13 segmentation for 16772902_532 vs. 16772902_635 (16772902_11000bp) - 38 segments

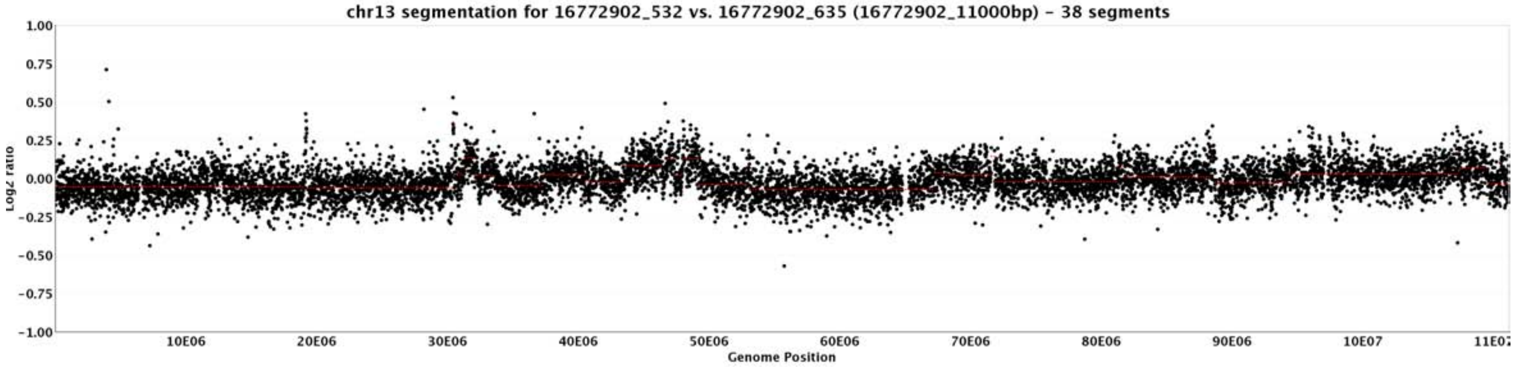

chr13_random segmentation for 16772902_532 vs. 16772902_635 (16772902_11000bp) - 1 segment

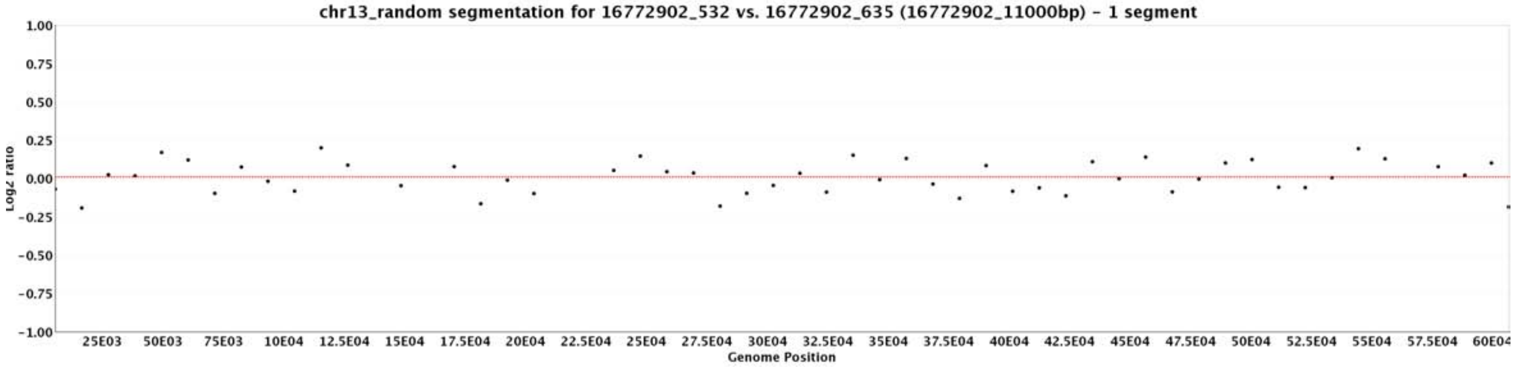

chr14 segmentation for 16772902_532 vs. 16772902_635 (16772902_11000bp) - 52 segments

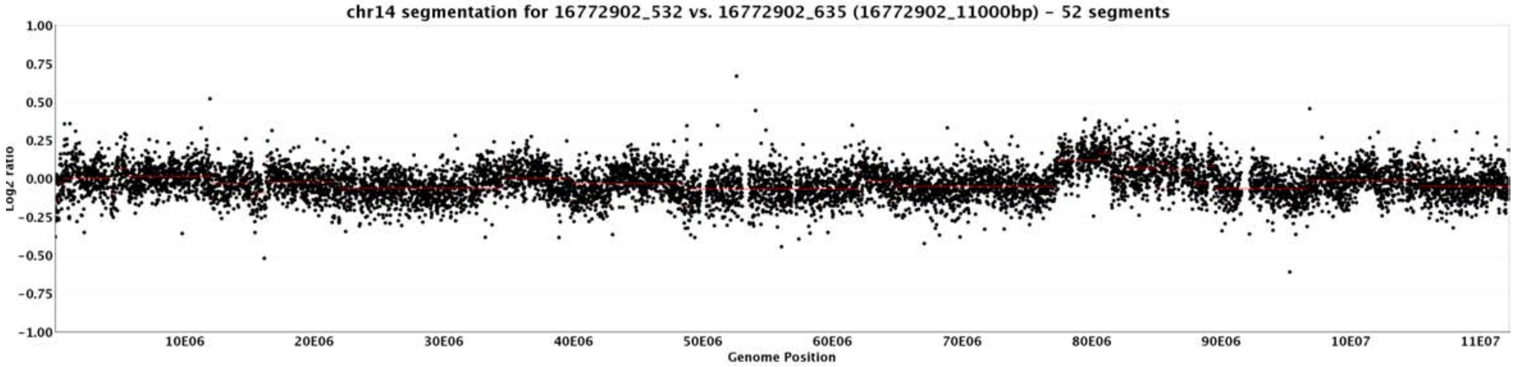

chr14_random segmentation for 16772902_532 vs. 16772902_635 (16772902_11000bp) - 1 segment

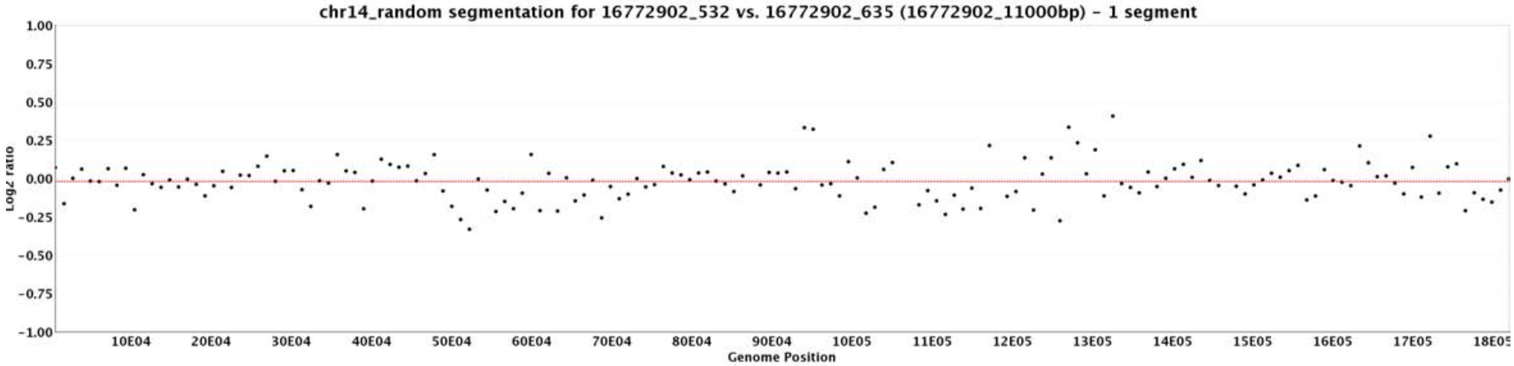

chr15 segmentation for 16772902_532 vs. 16772902_635 (16772902_11000bp) - 30 segments

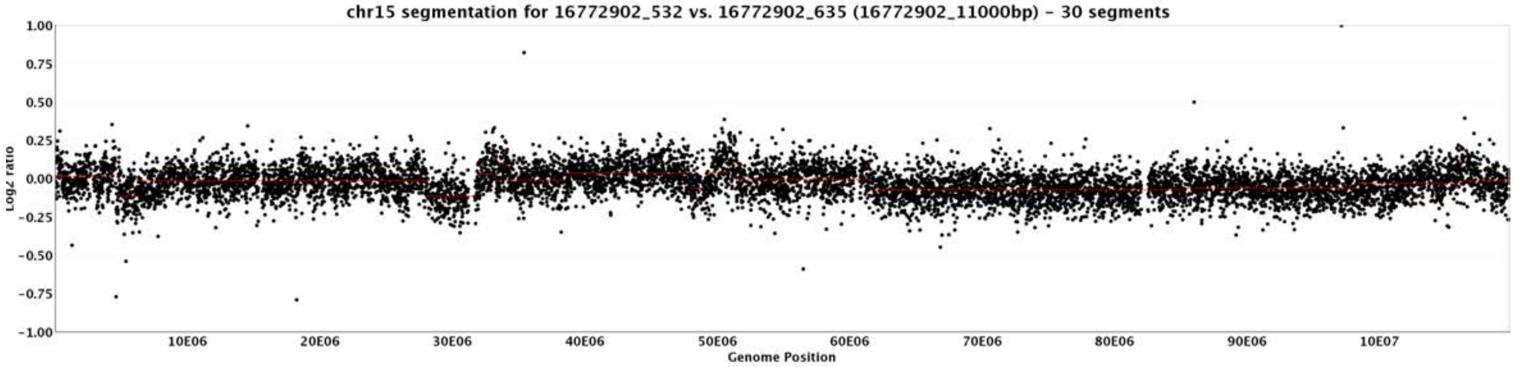

chr15_random segmentation for 16772902_532 vs. 16772902_635 (16772902_11000bp) - 2 segments

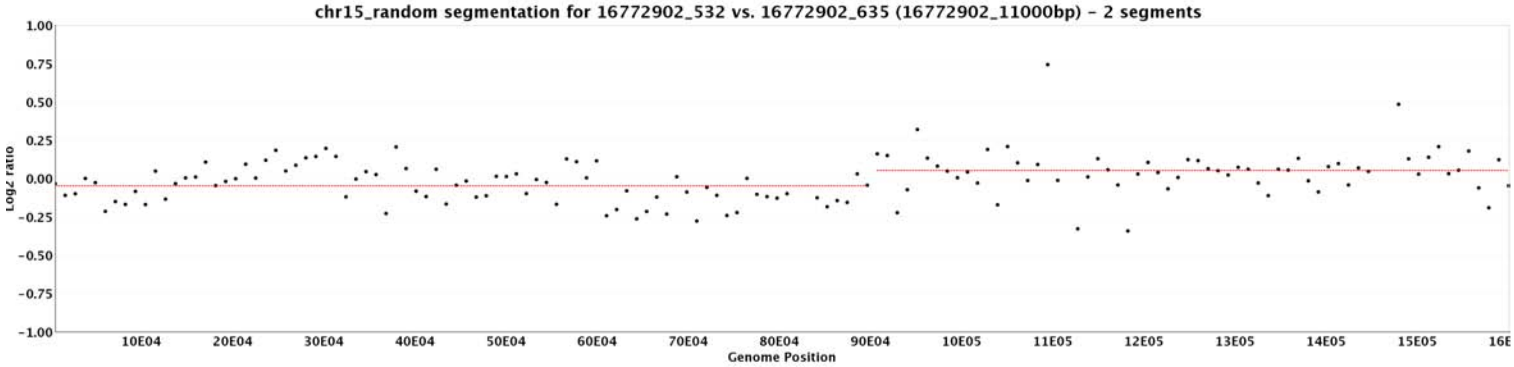

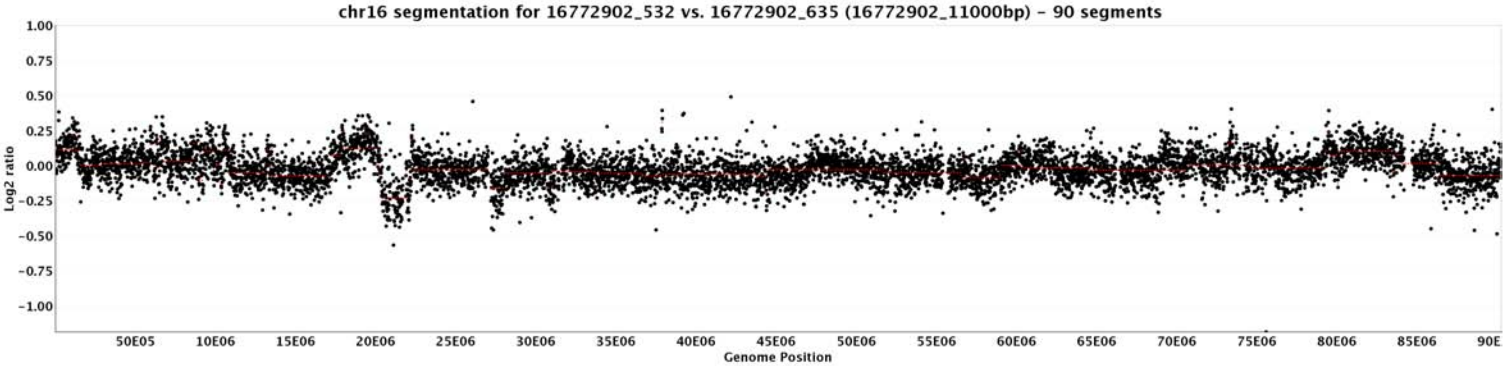
chr16 segmentation for 16772902_532 vs. 16772902_635 (16772902_11000bp) - 90 segments
Log2 ratio
Genome Position

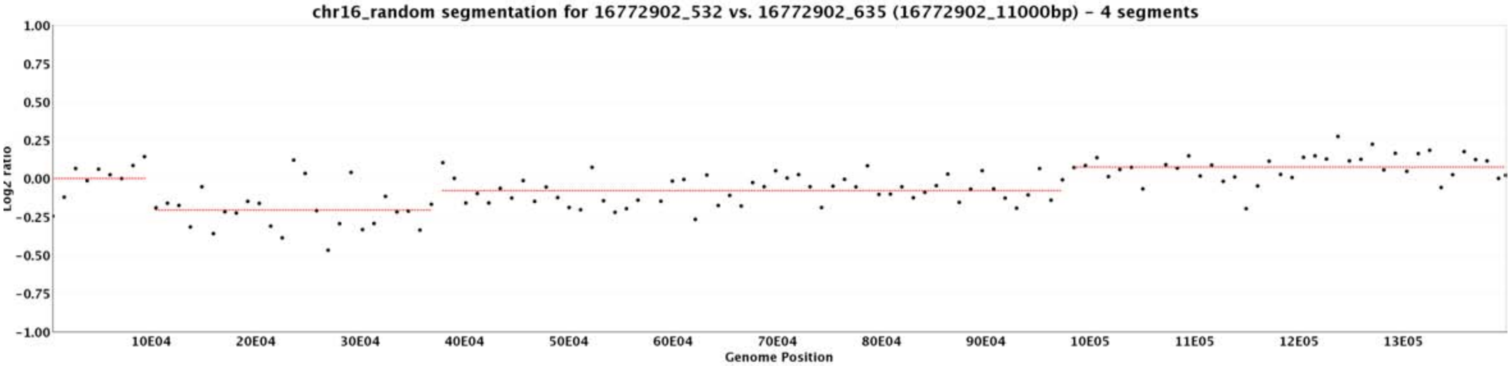
chr16_random segmentation for 16772902_532 vs. 16772902_635 (16772902_11000bp) - 4 segments
Log2 ratio
Genome Position

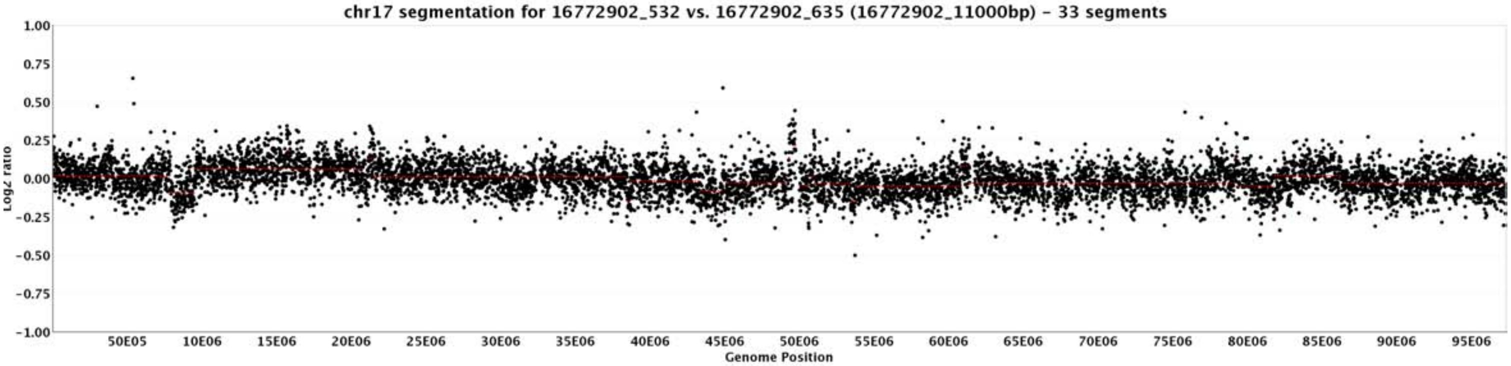
chr17 segmentation for 16772902_532 vs. 16772902_635 (16772902_11000bp) - 33 segments
Log2 ratio
Genome Position

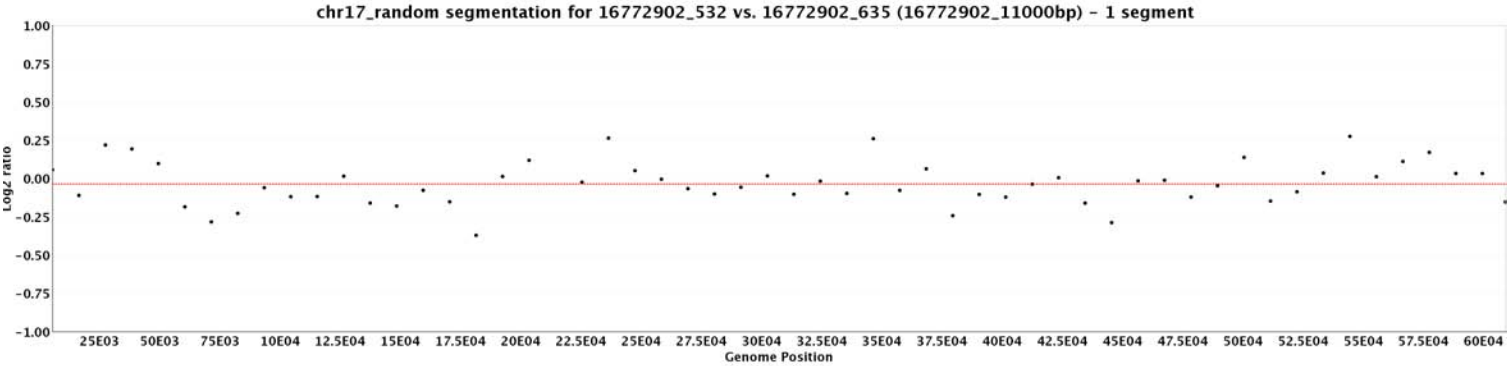
chr17_random segmentation for 16772902_532 vs. 16772902_635 (16772902_11000bp) - 1 segment
Log2 ratio
Genome Position

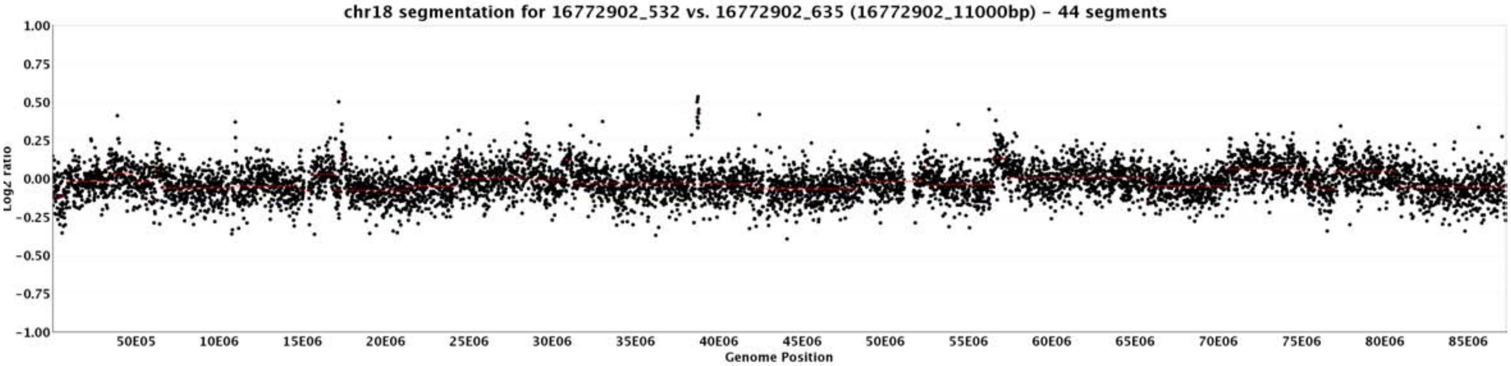
chr18 segmentation for 16772902_532 vs. 16772902_635 (16772902_11000bp) - 44 segments
Log2 ratio
Genome Position

chr18_random segmentation for 16772902_532 vs. 16772902_635 (16772902_11000bp) - 1 segment

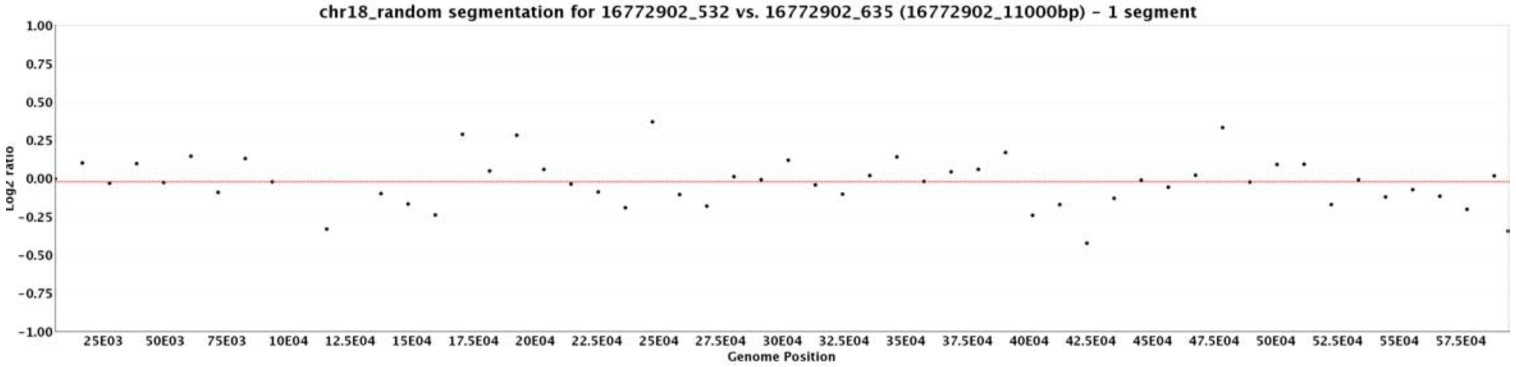

chr19 segmentation for 16772902_532 vs. 16772902_635 (16772902_11000bp) - 48 segments

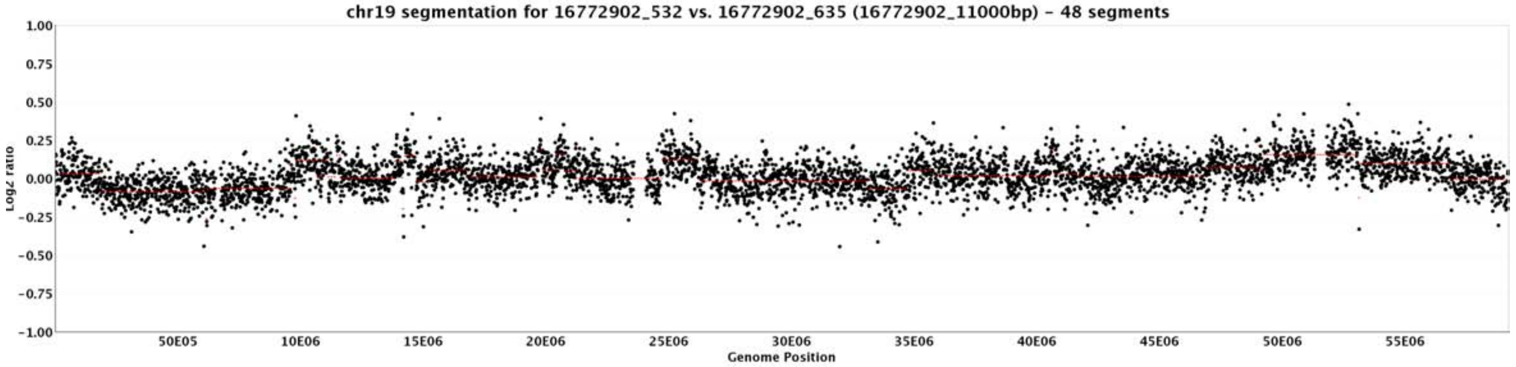

chr19_random segmentation for 16772902_532 vs. 16772902_635 (16772902_11000bp) - 3 segments

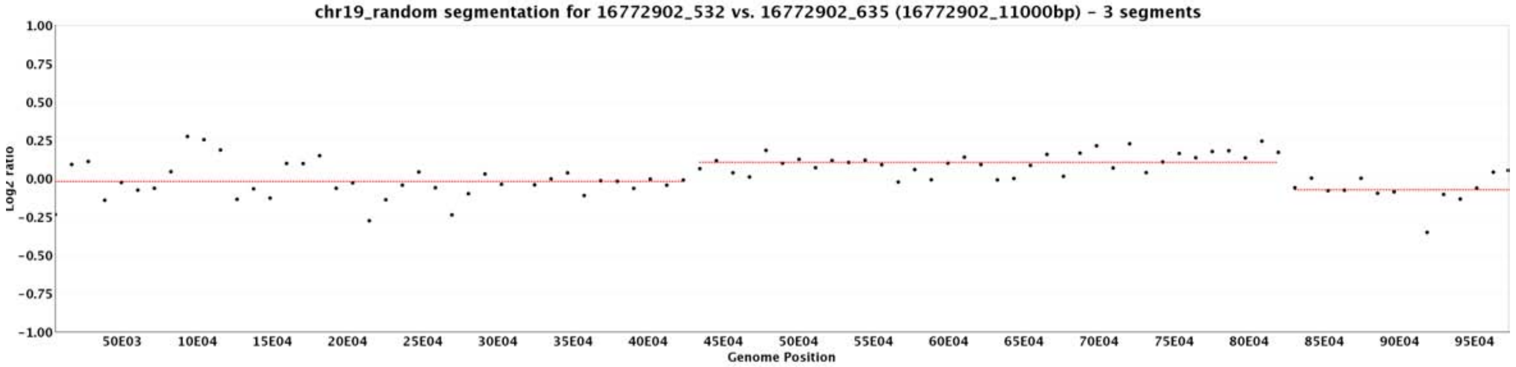

chr20 segmentation for 16772902_532 vs. 16772902_635 (16772902_11000bp) - 24 segments

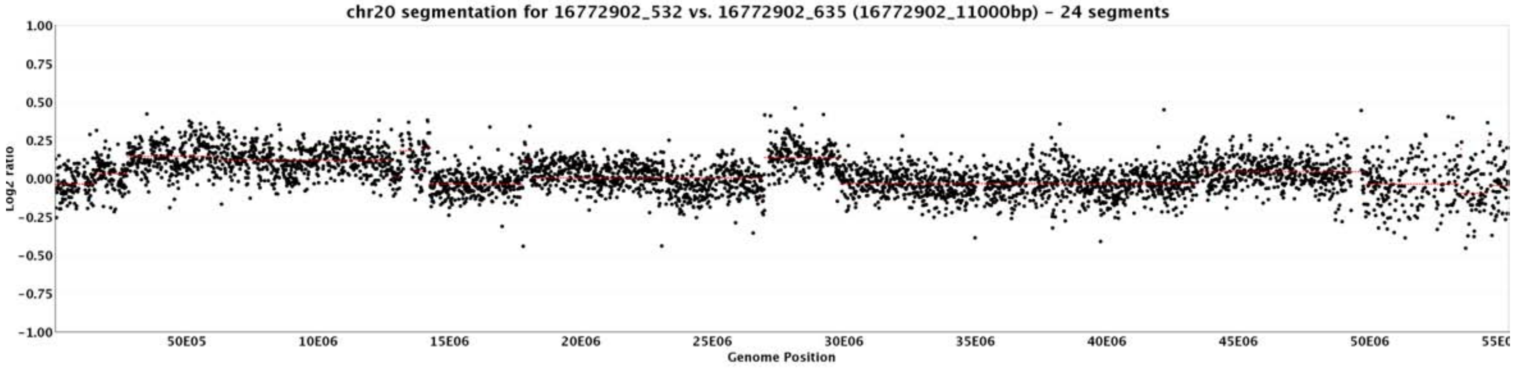

chr20_random segmentation for 16772902_532 vs. 16772902_635 (16772902_11000bp) - 2 segments

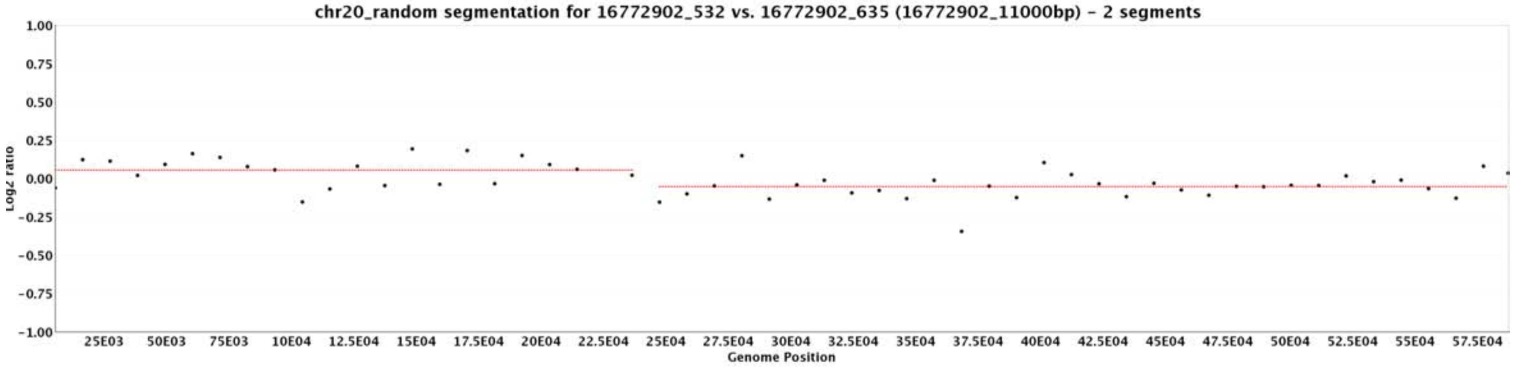

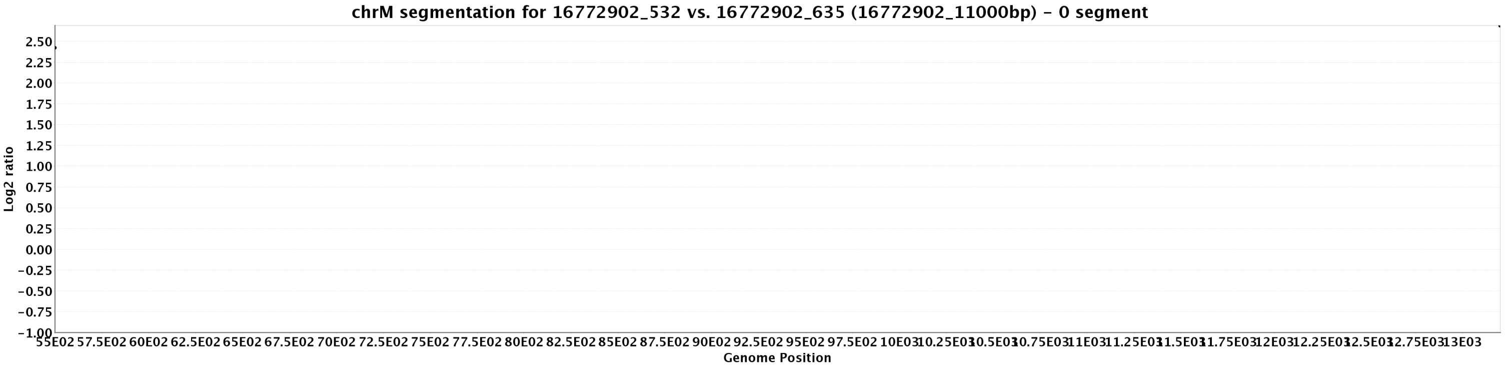
chrM segmentation for 16772902_532 vs. 16772902_635 (16772902_11000bp) - 0 segment
Log2 ratio
2.50 2.25 2.00 1.75 1.50 1.25 1.00 0.75 0.50 0.25 0.00 -0.25 -0.50 -0.75 -1.00
55E02 57.5E02 60E02 62.5E02 65E02 67.5E02 70E02 72.5E02 75E02 77.5E02 80E02 82.5E02 85E02 87.5E02 90E02 92.5E02 95E02 97.5E02 10E03 10.25E03 10.5E03 10.75E03 11E03 11.25E03 11.5E03 11.75E03 12E03 12.25E03 12.5E03 12.75E03 13E03
Genome Position

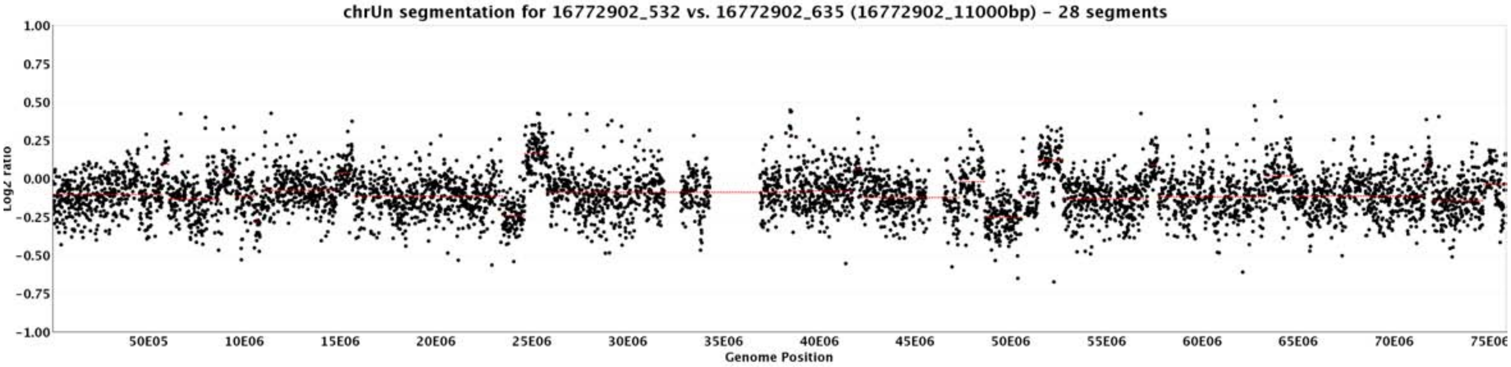
chrUn segmentation for 16772902_532 vs. 16772902_635 (16772902_11000bp) - 28 segments
Log2 ratio
1.00 0.75 0.50 0.25 0.00 -0.25 -0.50 -0.75 -1.00
50E05 10E06 15E06 20E06 25E06 30E06 35E06 40E06 45E06 50E06 55E06 60E06 65E06 70E06 75E06
Genome Position

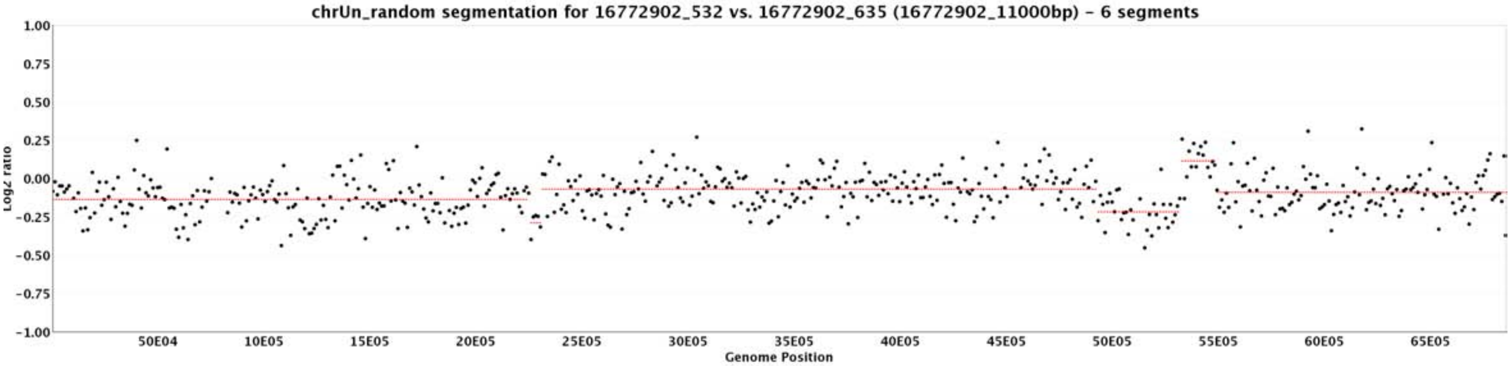
chrUn_random segmentation for 16772902_532 vs. 16772902_635 (16772902_11000bp) - 6 segments
Log2 ratio
1.00 0.75 0.50 0.25 0.00 -0.25 -0.50 -0.75 -1.00
50E04 10E05 15E05 20E05 25E05 30E05 35E05 40E05 45E05 50E05 55E05 60E05 65E05
Genome Position

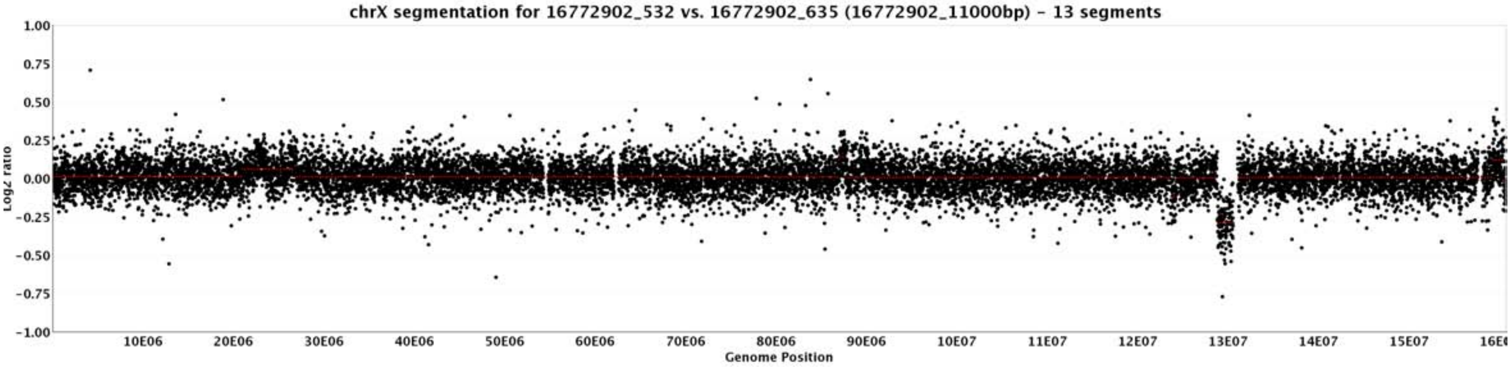
chrX segmentation for 16772902_532 vs. 16772902_635 (16772902_11000bp) - 13 segments
Log2 ratio
1.00 0.75 0.50 0.25 0.00 -0.25 -0.50 -0.75 -1.00
10E06 20E06 30E06 40E06 50E06 60E06 70E06 80E06 90E06 10E07 11E07 12E07 13E07 14E07 15E07
Genome Position

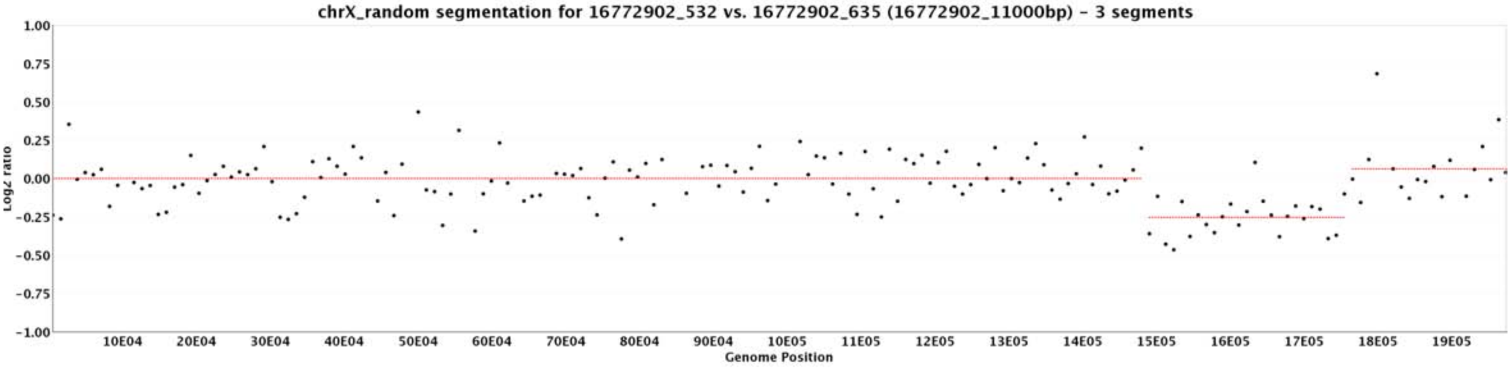
chrX_random segmentation for 16772902_532 vs. 16772902_635 (16772902_11000bp) - 3 segments
Log2 ratio
1.00 0.75 0.50 0.25 0.00 -0.25 -0.50 -0.75 -1.00
10E04 20E04 30E04 40E04 50E04 60E04 70E04 80E04 90E04 10E05 11E05 12E05 13E05 14E05 15E05 16E05 17E05 18E05 19E05
Genome Position

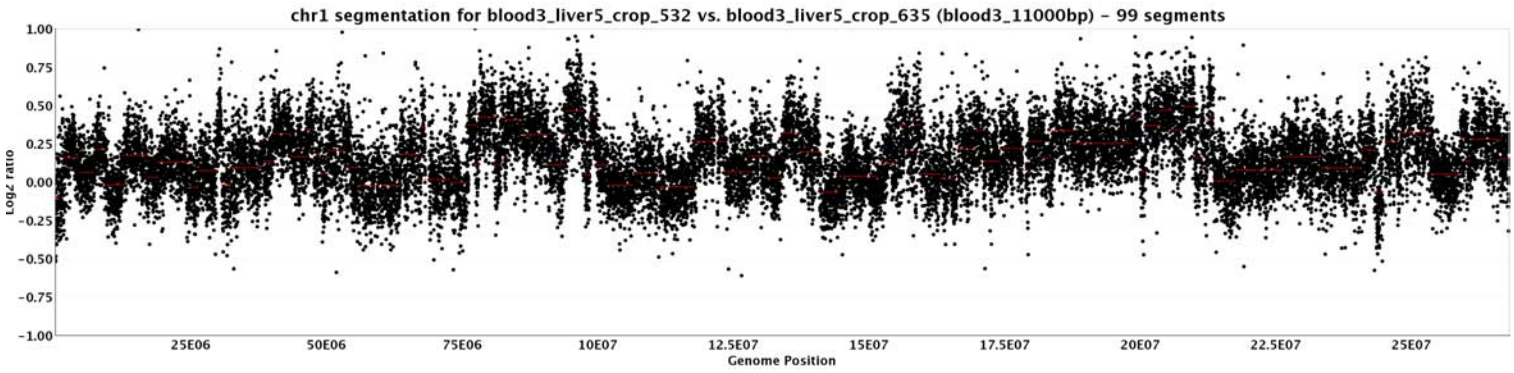
chr1 segmentation for blood3_liver5_crop_532 vs. blood3_liver5_crop_635 (blood3_11000bp) - 99 segments
Log2 ratio
1.00
0.75
0.50
0.25
0.00
-0.25
-0.50
-0.75
-1.00
25E06
50E06
75E06
10E07
12.5E07
15E07
17.5E07
20E07
22.5E07
25E07
Genome Position

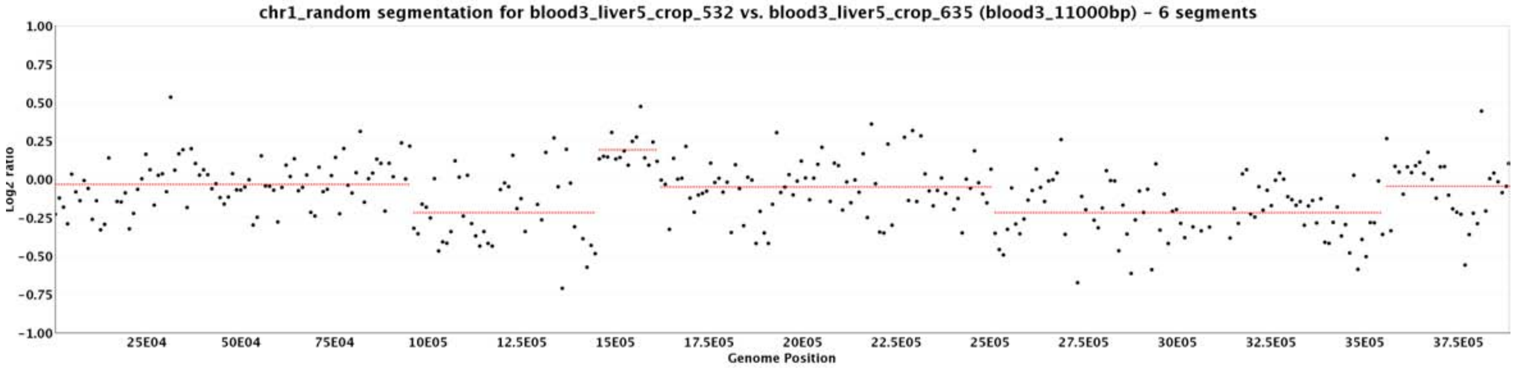
chr1_random segmentation for blood3_liver5_crop_532 vs. blood3_liver5_crop_635 (blood3_11000bp) - 6 segments
Log2 ratio
1.00
0.75
0.50
0.25
0.00
-0.25
-0.50
-0.75
-1.00
25E04
50E04
75E04
10E05
12.5E05
15E05
17.5E05
20E05
22.5E05
25E05
27.5E05
30E05
32.5E05
35E05
37.5E05
Genome Position

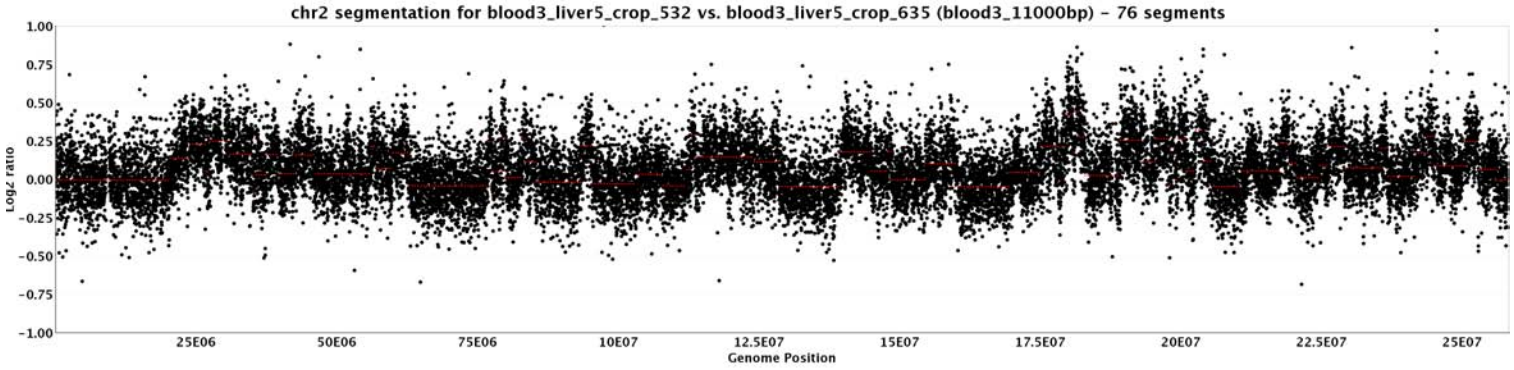
chr2 segmentation for blood3_liver5_crop_532 vs. blood3_liver5_crop_635 (blood3_11000bp) - 76 segments
Log2 ratio
1.00
0.75
0.50
0.25
0.00
-0.25
-0.50
-0.75
-1.00
25E06
50E06
75E06
10E07
12.5E07
15E07
17.5E07
20E07
22.5E07
25E07
Genome Position

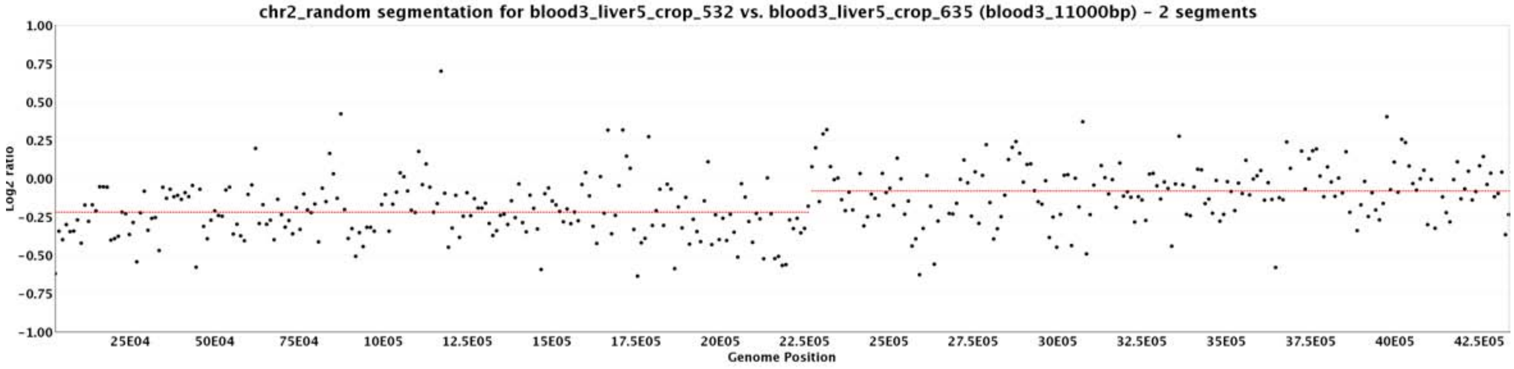
chr2_random segmentation for blood3_liver5_crop_532 vs. blood3_liver5_crop_635 (blood3_11000bp) - 2 segments
Log2 ratio
1.00
0.75
0.50
0.25
0.00
-0.25
-0.50
-0.75
-1.00
25E04
50E04
75E04
10E05
12.5E05
15E05
17.5E05
20E05
22.5E05
25E05
27.5E05
30E05
32.5E05
35E05
37.5E05
40E05
42.5E05
Genome Position

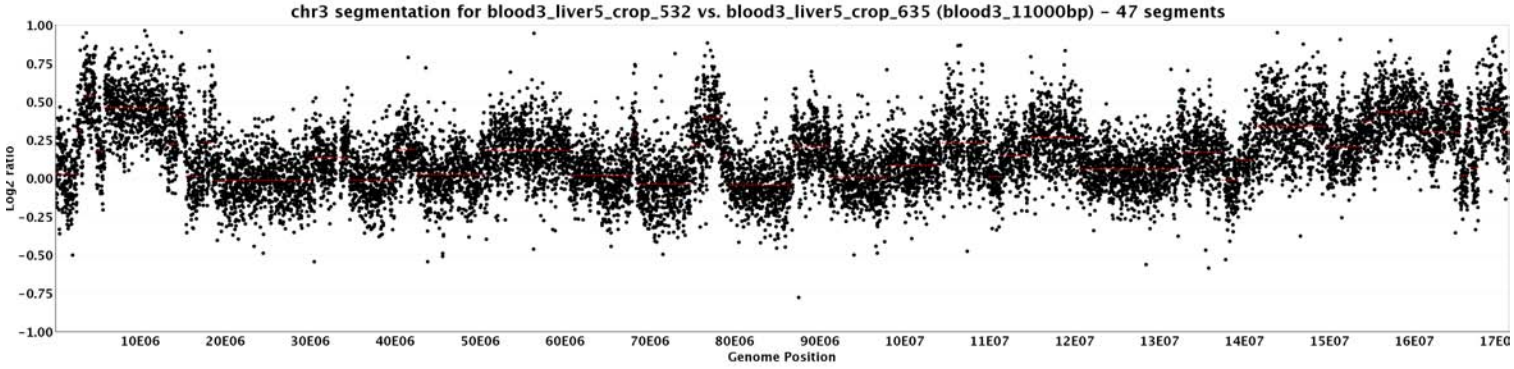
chr3 segmentation for blood3_liver5_crop_532 vs. blood3_liver5_crop_635 (blood3_11000bp) - 47 segments
Log2 ratio
1.00
0.75
0.50
0.25
0.00
-0.25
-0.50
-0.75
-1.00
10E06
20E06
30E06
40E06
50E06
60E06
70E06
80E06
90E06
10E07
11E07
12E07
13E07
14E07
15E07
16E07
Genome Position

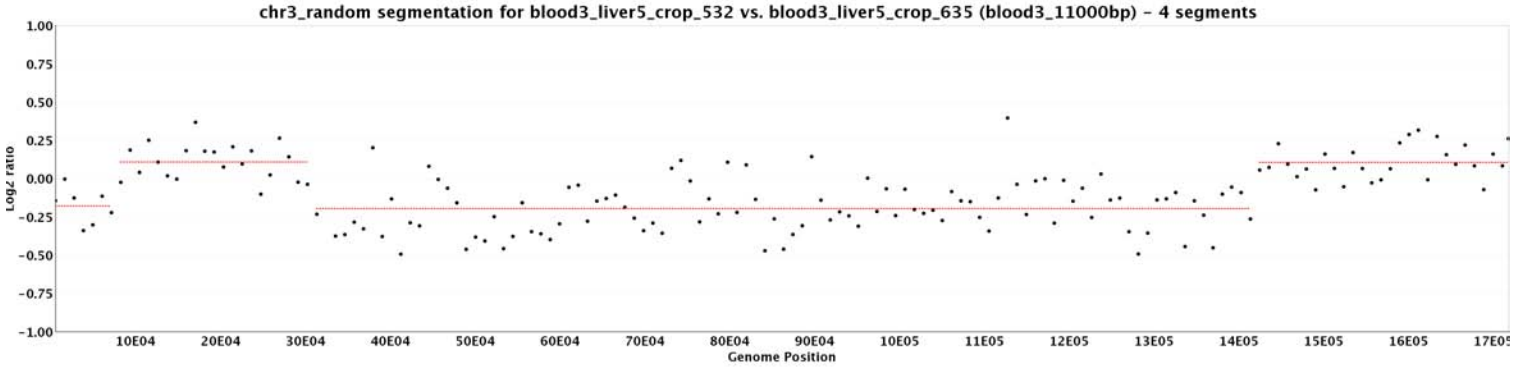
chr3_random segmentation for blood3_liver5_crop_532 vs. blood3_liver5_crop_635 (blood3_11000bp) - 4 segments
Log2 ratio
Genome Position

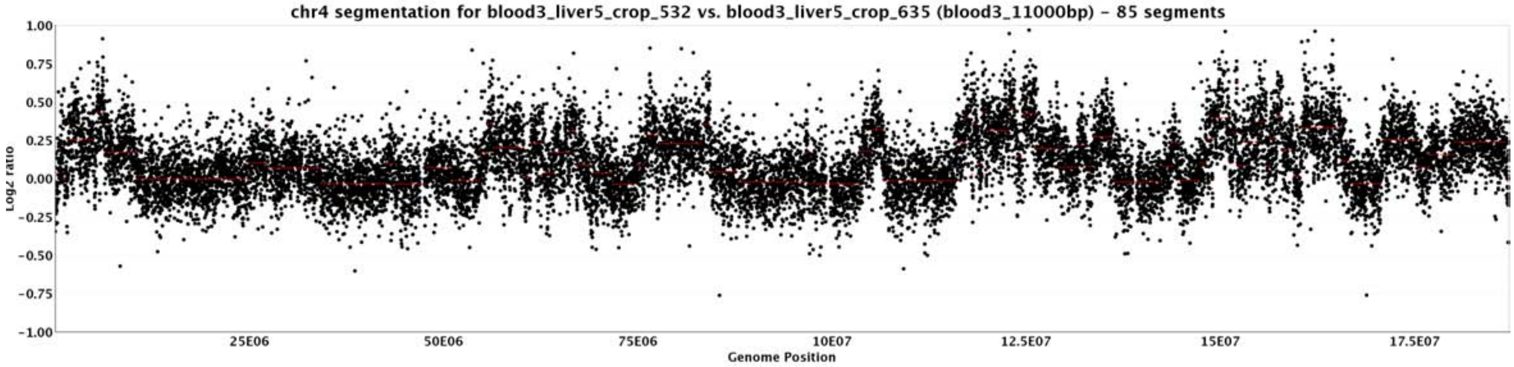
chr4 segmentation for blood3_liver5_crop_532 vs. blood3_liver5_crop_635 (blood3_11000bp) - 85 segments
Log2 ratio
Genome Position

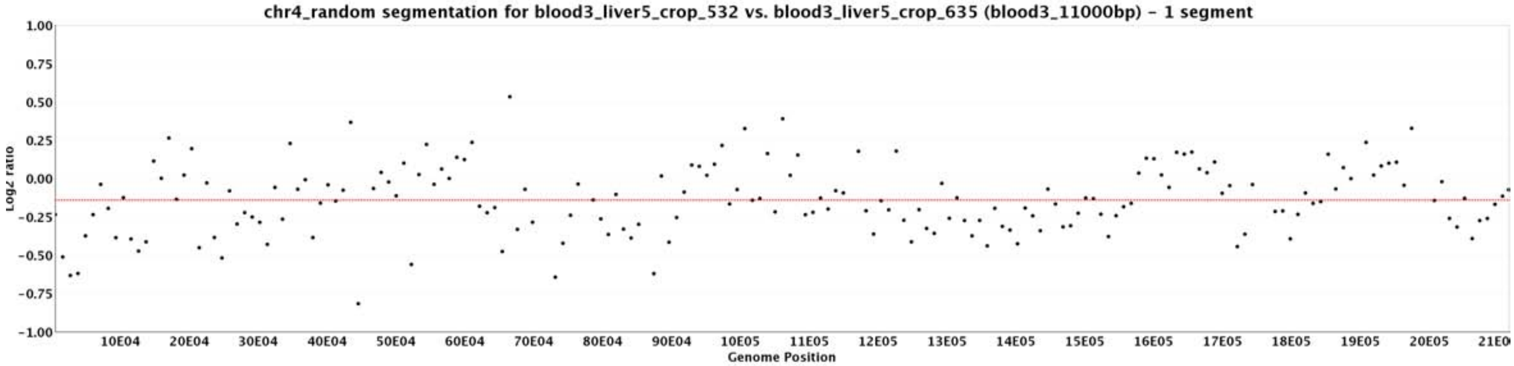
chr4_random segmentation for blood3_liver5_crop_532 vs. blood3_liver5_crop_635 (blood3_11000bp) - 1 segment
Log2 ratio
Genome Position

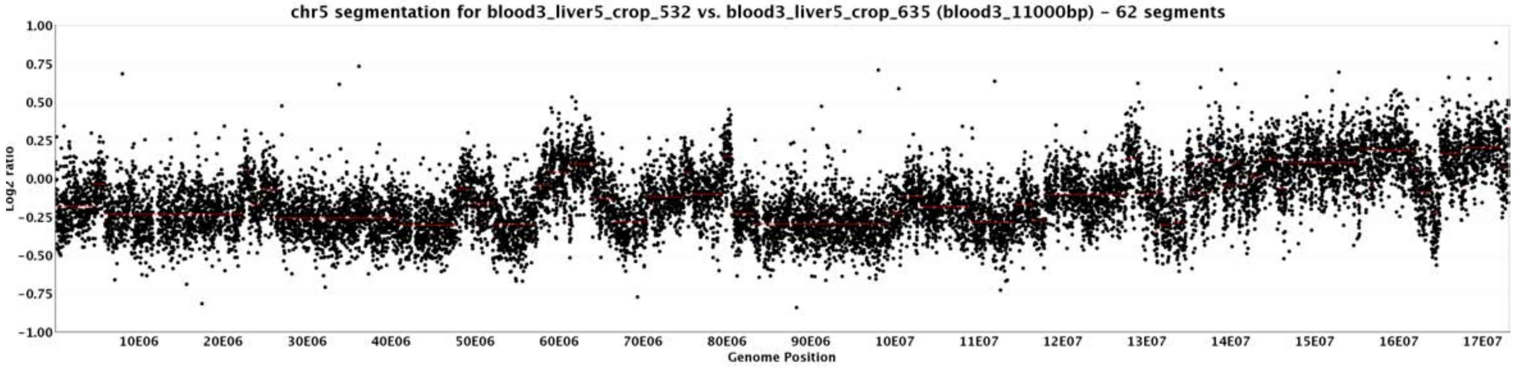
chr5 segmentation for blood3_liver5_crop_532 vs. blood3_liver5_crop_635 (blood3_11000bp) - 62 segments
Log2 ratio
Genome Position

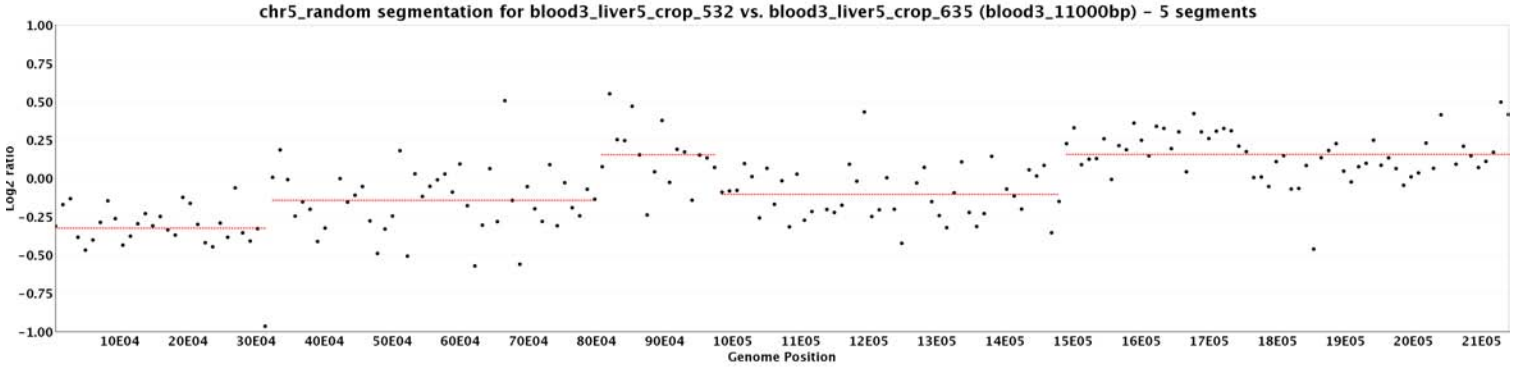
chr5_random segmentation for blood3_liver5_crop_532 vs. blood3_liver5_crop_635 (blood3_11000bp) - 5 segments
Log2 ratio
Genome Position

chr6 segmentation for blood3_liver5_crop_532 vs. blood3_liver5_crop_635 (blood3_11000bp) - 85 segments

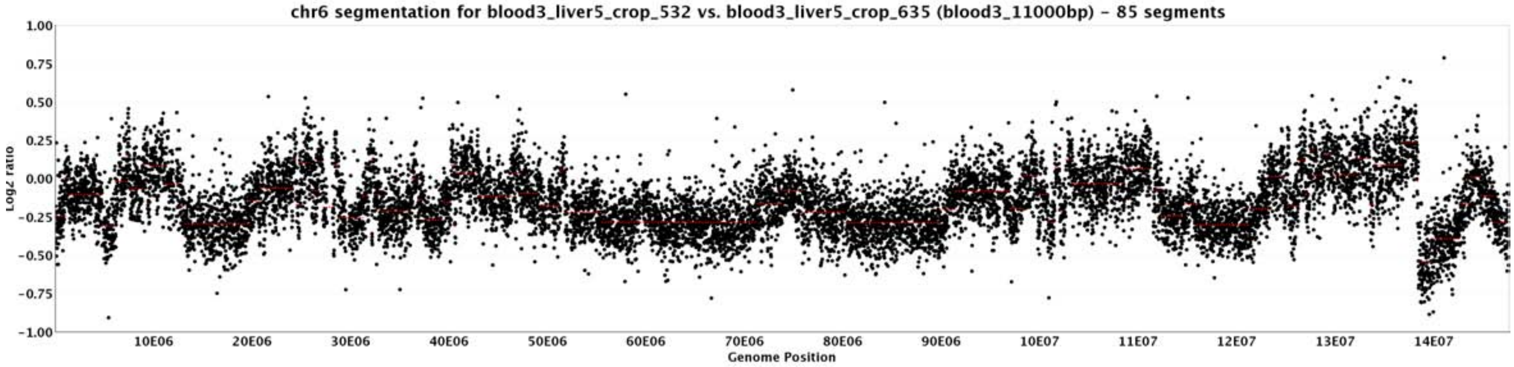

chr6_random segmentation for blood3_liver5_crop_532 vs. blood3_liver5_crop_635 (blood3_11000bp) - 1 segment

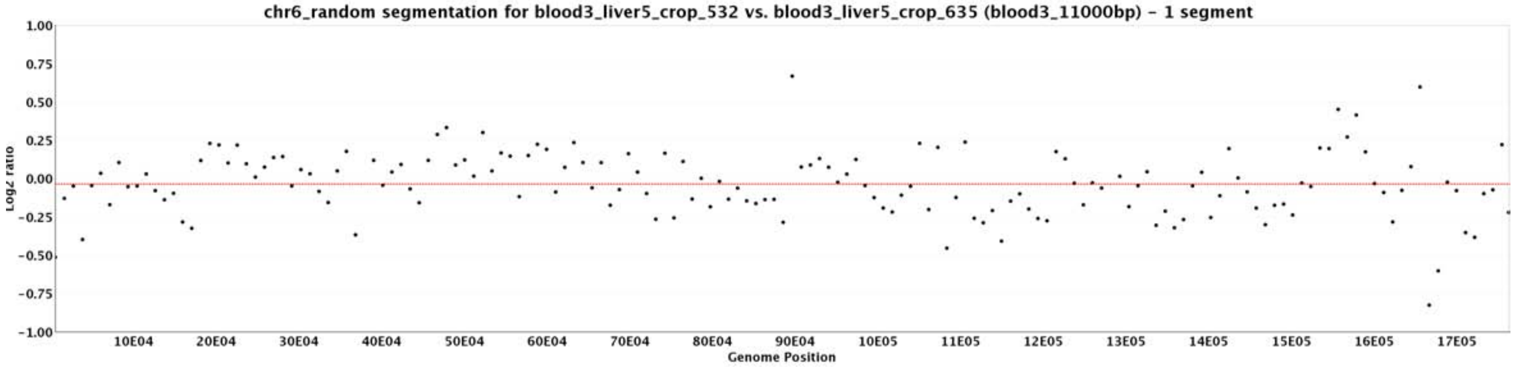

chr7 segmentation for blood3_liver5_crop_532 vs. blood3_liver5_crop_635 (blood3_11000bp) - 57 segments

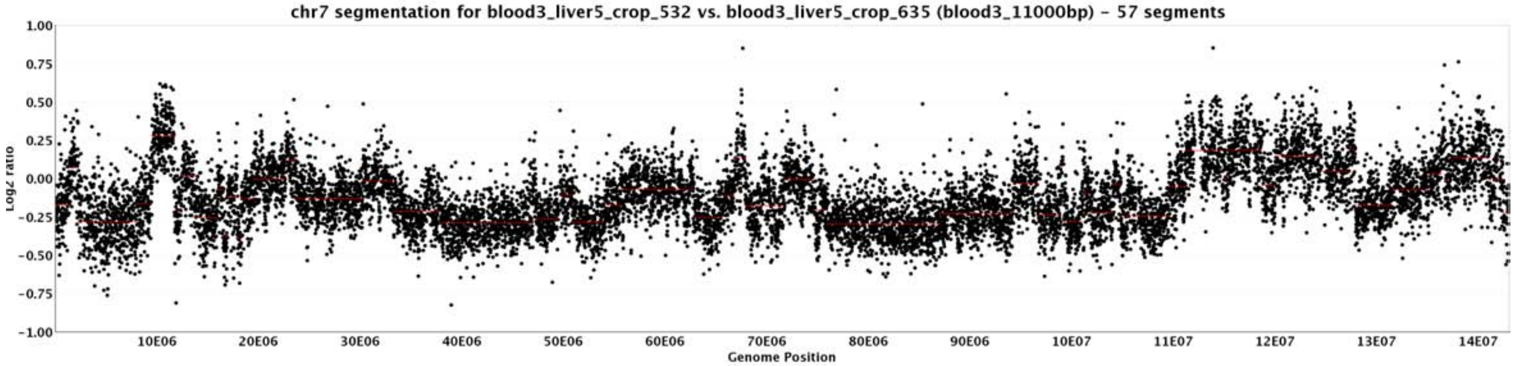

chr7_random segmentation for blood3_liver5_crop_532 vs. blood3_liver5_crop_635 (blood3_11000bp) - 1 segment

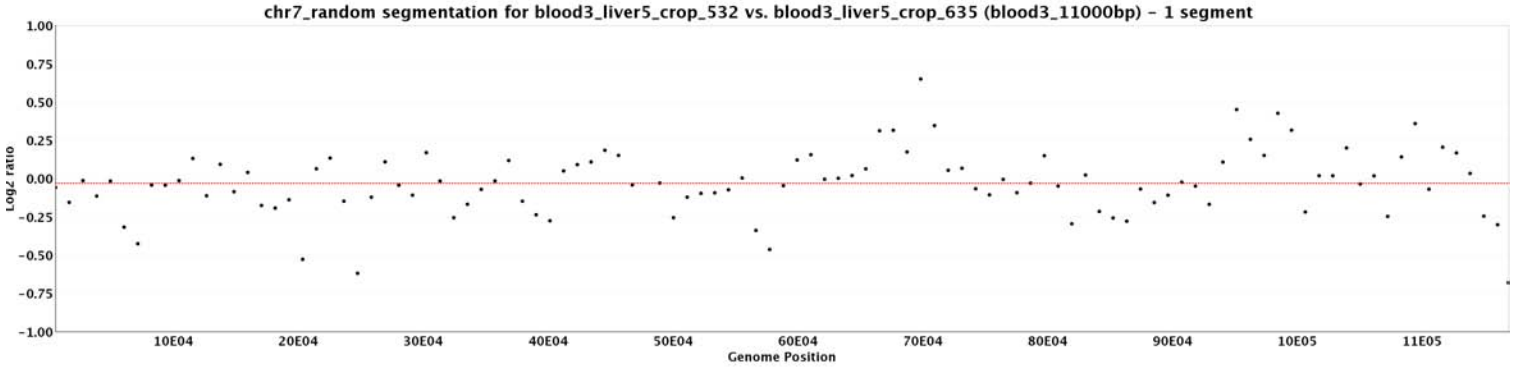

chr8 segmentation for blood3_liver5_crop_532 vs. blood3_liver5_crop_635 (blood3_11000bp) - 72 segments

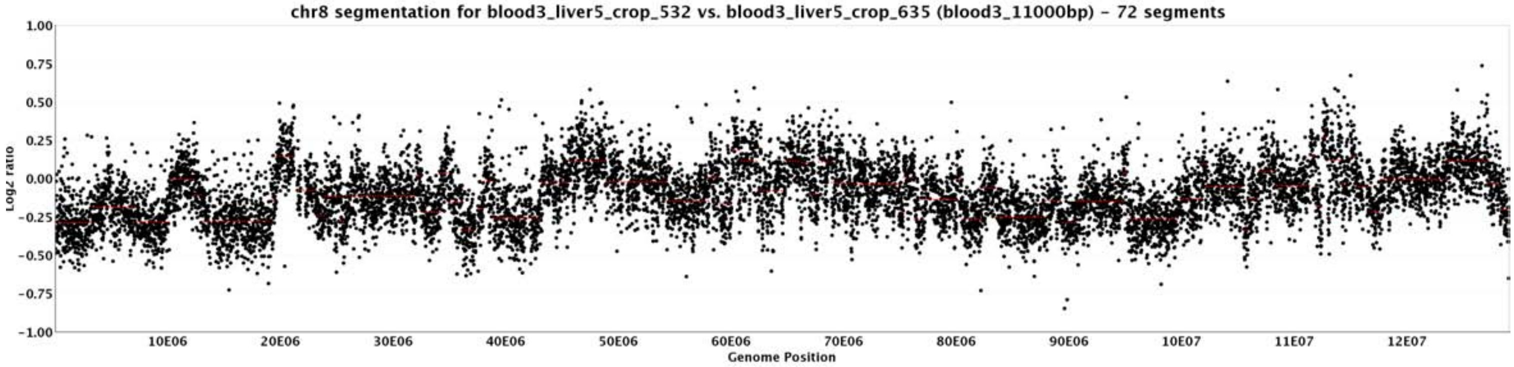

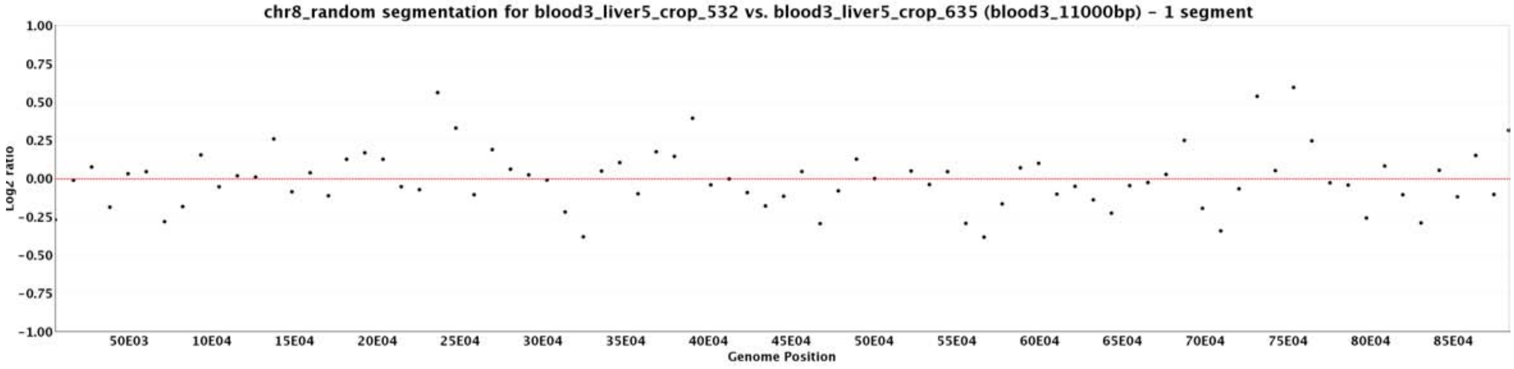
chr8_random segmentation for blood3_liver5_crop_532 vs. blood3_liver5_crop_635 (blood3_11000bp) - 1 segment
Log2 ratio
Genome Position

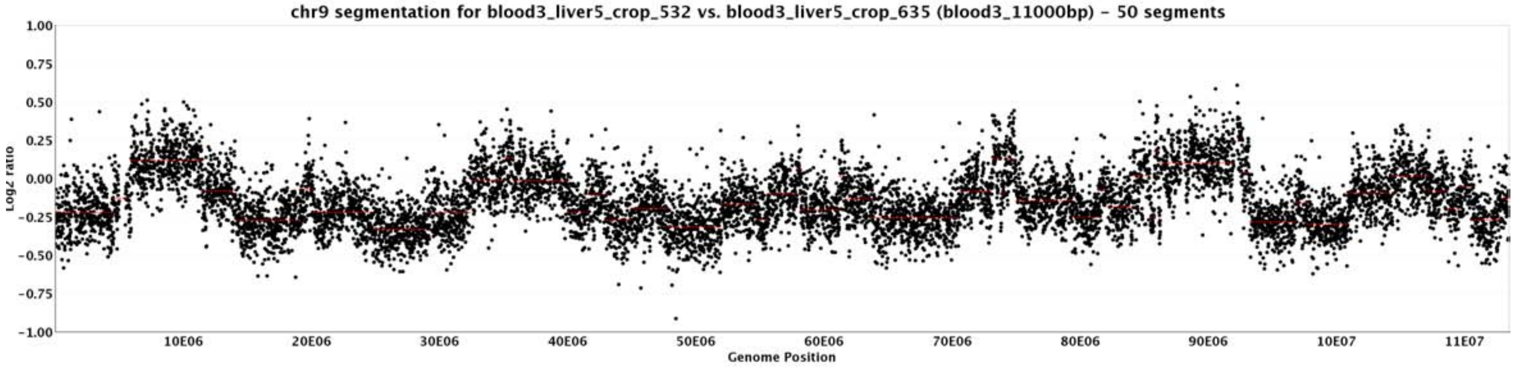
chr9 segmentation for blood3_liver5_crop_532 vs. blood3_liver5_crop_635 (blood3_11000bp) - 50 segments
Log2 ratio
Genome Position

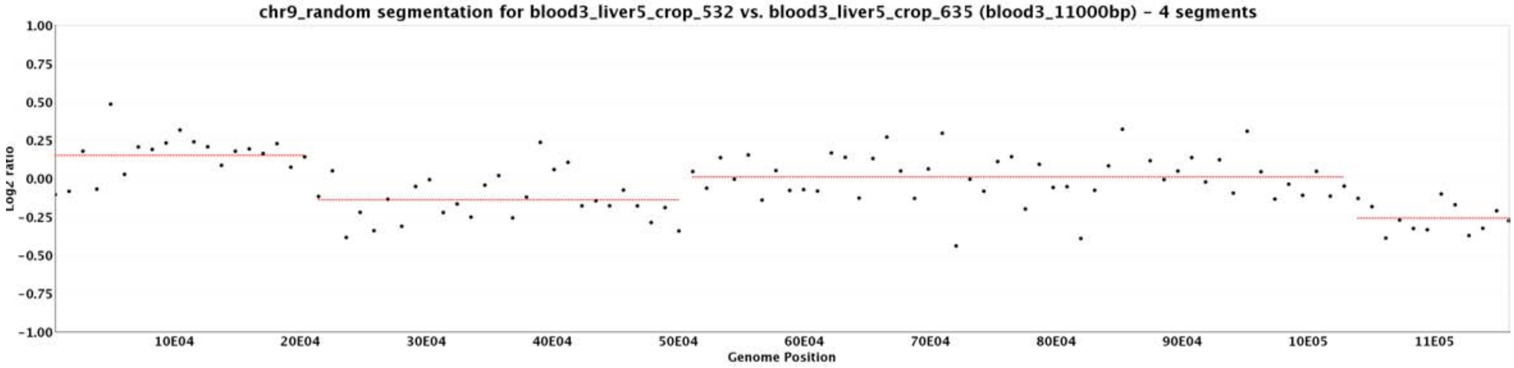
chr9_random segmentation for blood3_liver5_crop_532 vs. blood3_liver5_crop_635 (blood3_11000bp) - 4 segments
Log2 ratio
Genome Position

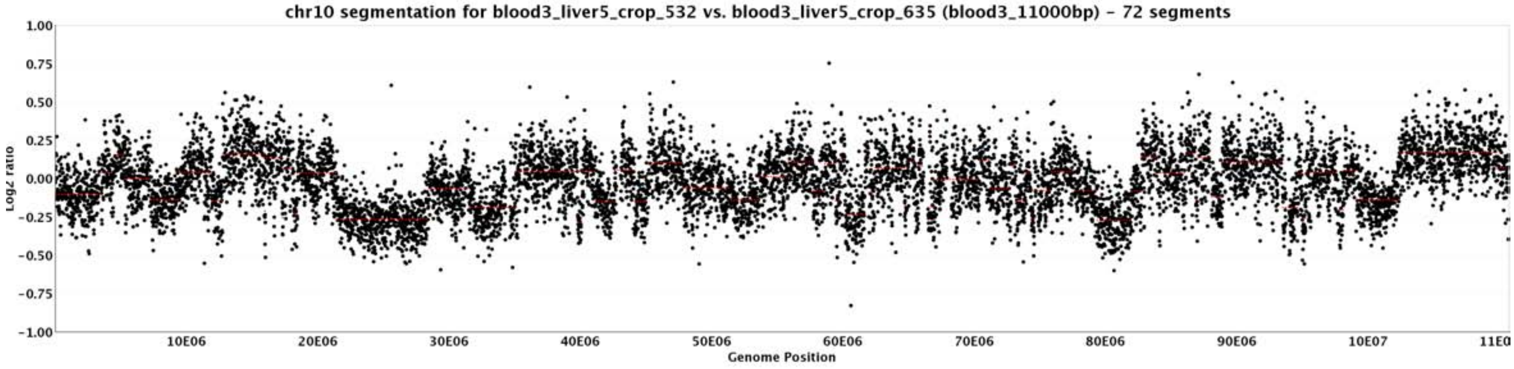
chr10 segmentation for blood3_liver5_crop_532 vs. blood3_liver5_crop_635 (blood3_11000bp) - 72 segments
Log2 ratio
Genome Position

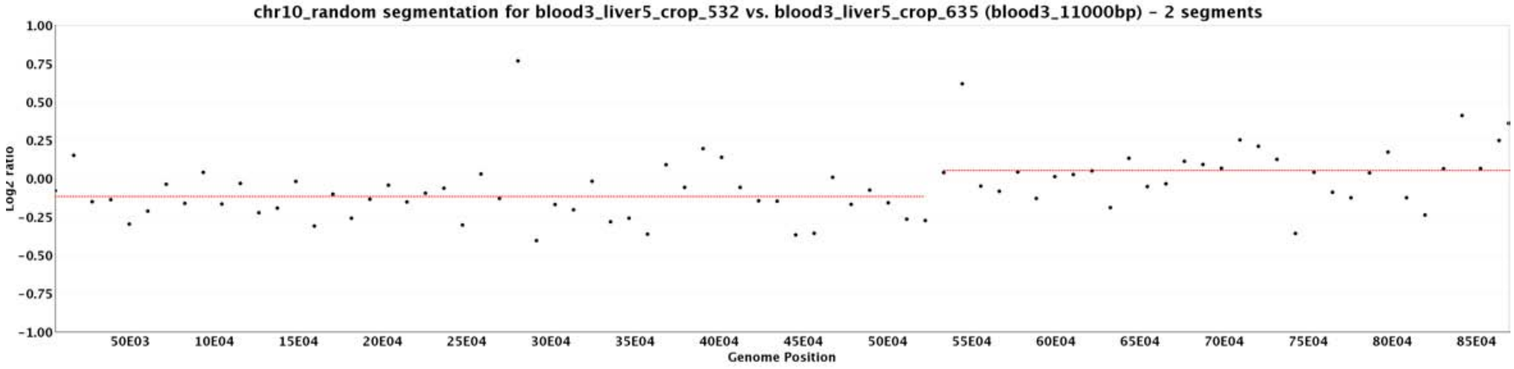
chr10_random segmentation for blood3_liver5_crop_532 vs. blood3_liver5_crop_635 (blood3_11000bp) - 2 segments
Log2 ratio
Genome Position

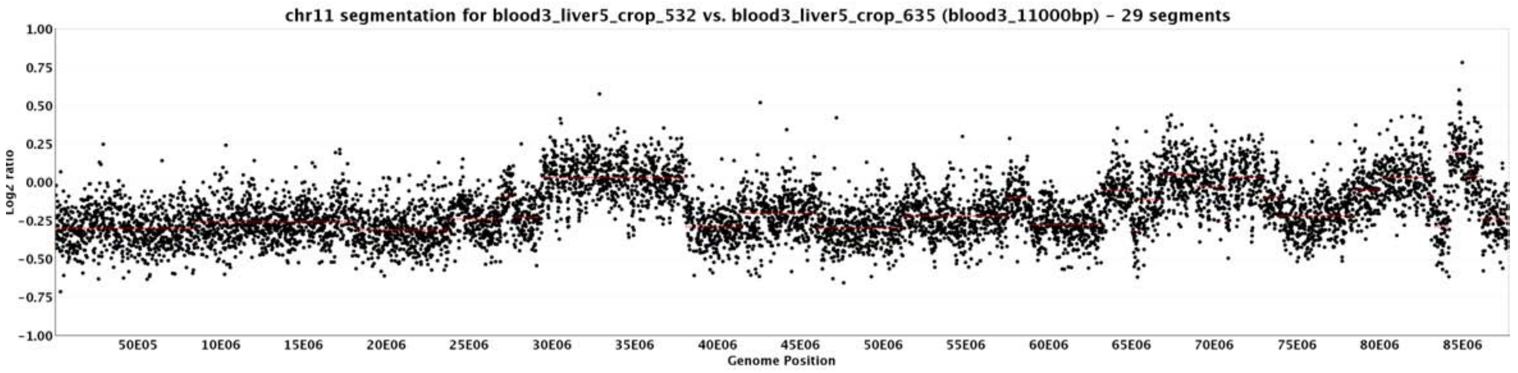
chr11 segmentation for blood3_liver5_crop_532 vs. blood3_liver5_crop_635 (blood3_11000bp) - 29 segments
Log2 ratio
Genome Position

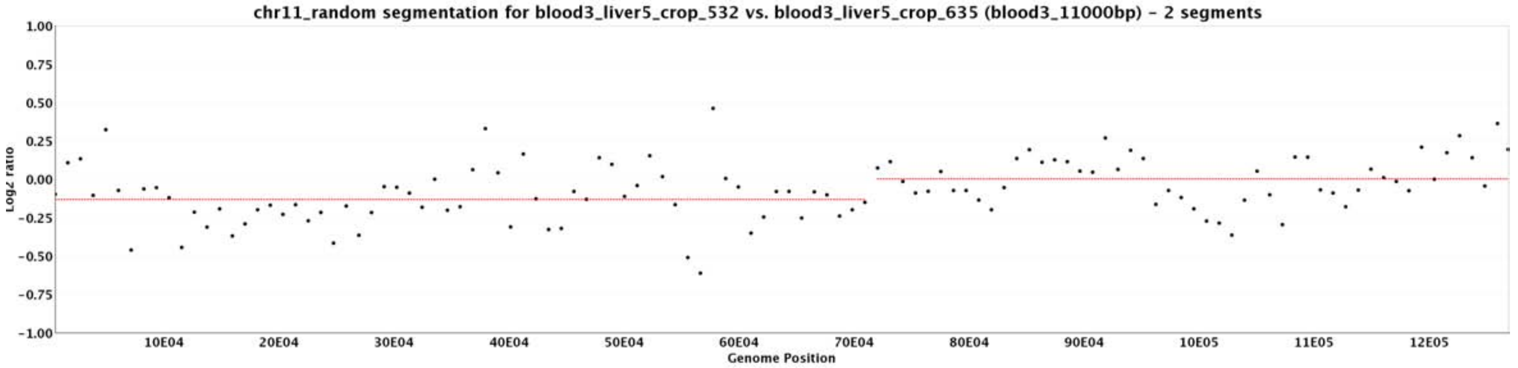
chr11_random segmentation for blood3_liver5_crop_532 vs. blood3_liver5_crop_635 (blood3_11000bp) - 2 segments
Log2 ratio
Genome Position

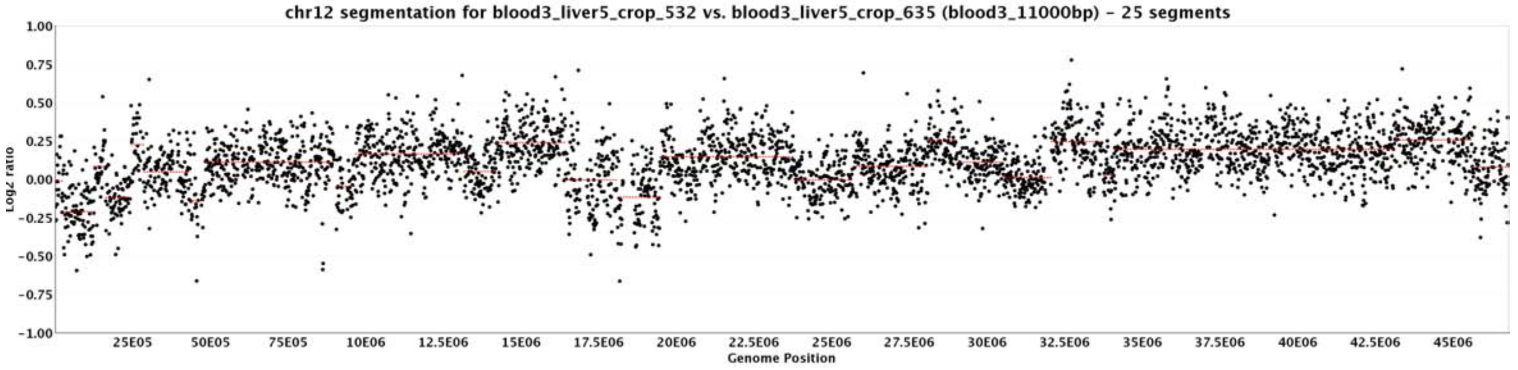
chr12 segmentation for blood3_liver5_crop_532 vs. blood3_liver5_crop_635 (blood3_11000bp) - 25 segments
Log2 ratio
Genome Position

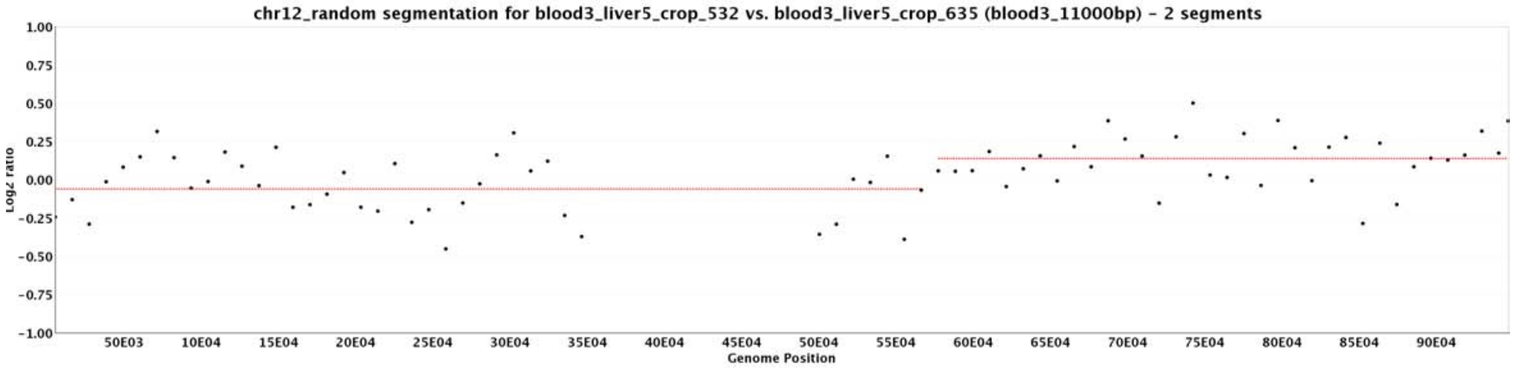
chr12_random segmentation for blood3_liver5_crop_532 vs. blood3_liver5_crop_635 (blood3_11000bp) - 2 segments
Log2 ratio
Genome Position

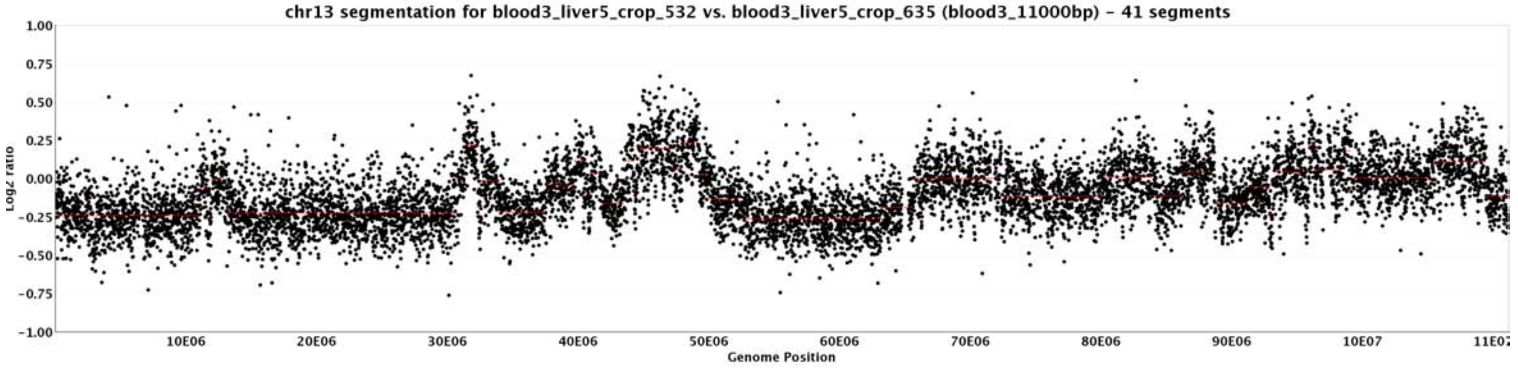
chr13 segmentation for blood3_liver5_crop_532 vs. blood3_liver5_crop_635 (blood3_11000bp) - 41 segments
Log2 ratio
Genome Position

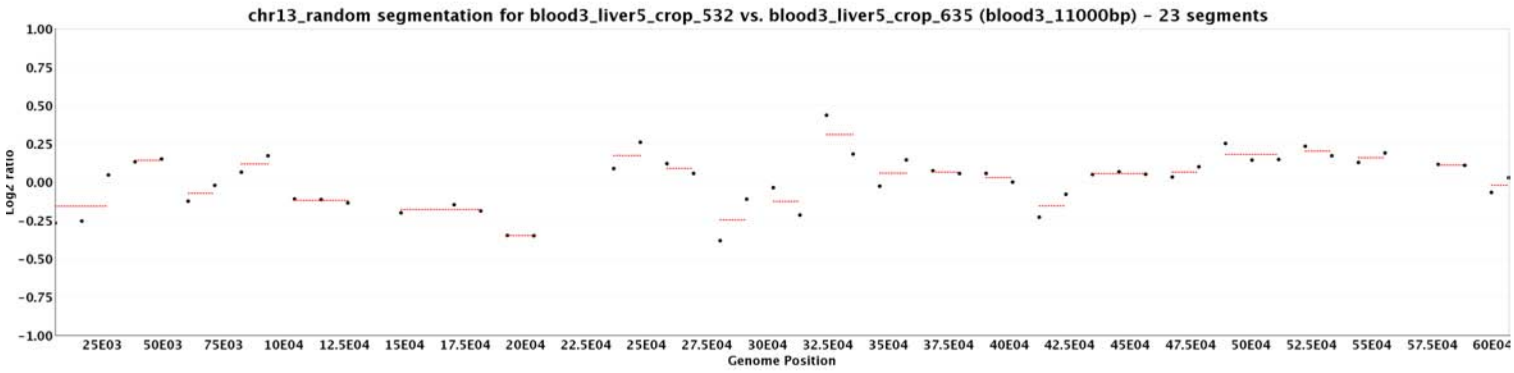
chr13_random segmentation for blood3_liver5_crop_532 vs. blood3_liver5_crop_635 (blood3_11000bp) - 23 segments
Log2 ratio
Genome Position

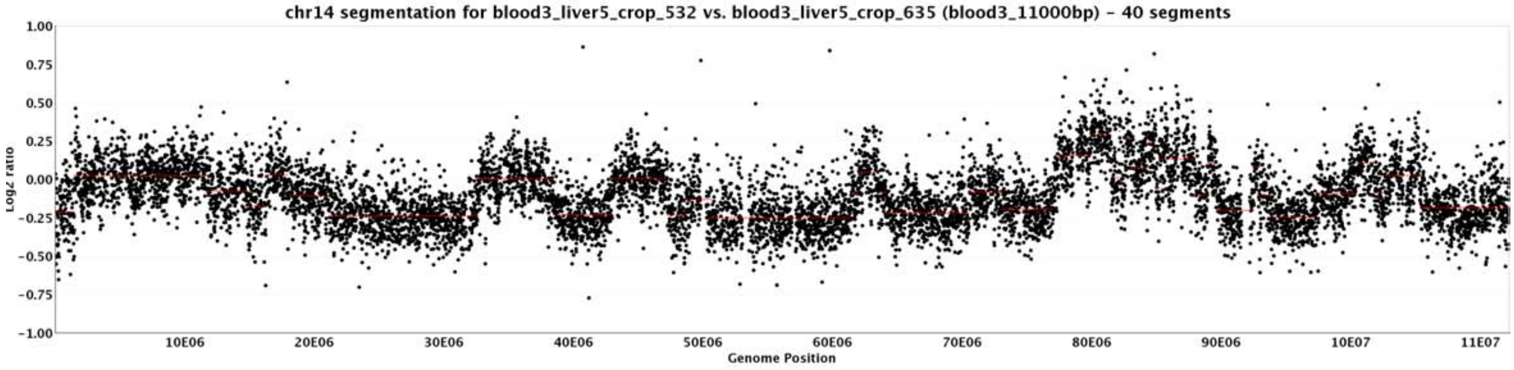
chr14 segmentation for blood3_liver5_crop_532 vs. blood3_liver5_crop_635 (blood3_11000bp) - 40 segments
Log2 ratio
Genome Position

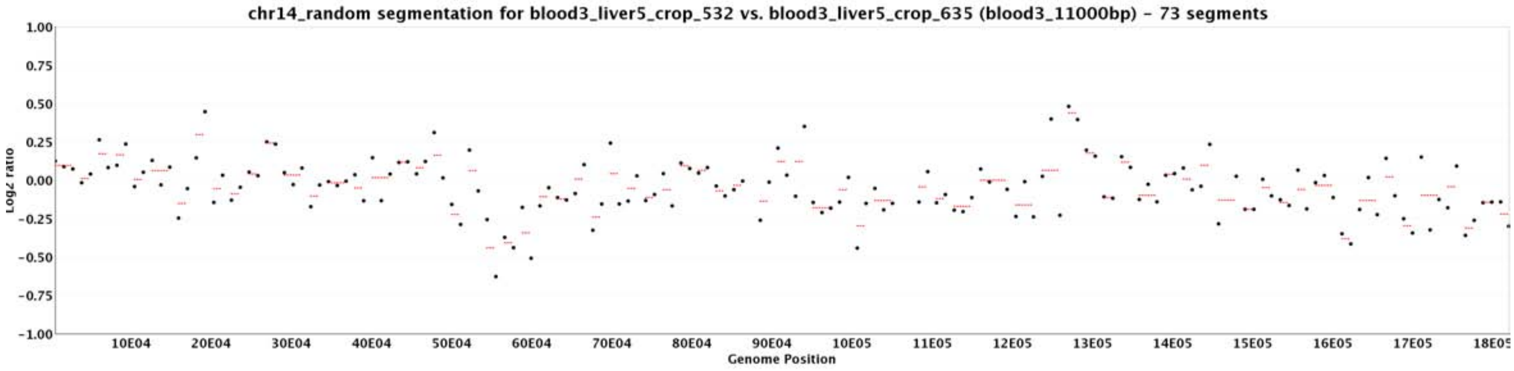
chr14_random segmentation for blood3_liver5_crop_532 vs. blood3_liver5_crop_635 (blood3_11000bp) - 73 segments
Log2 ratio
Genome Position

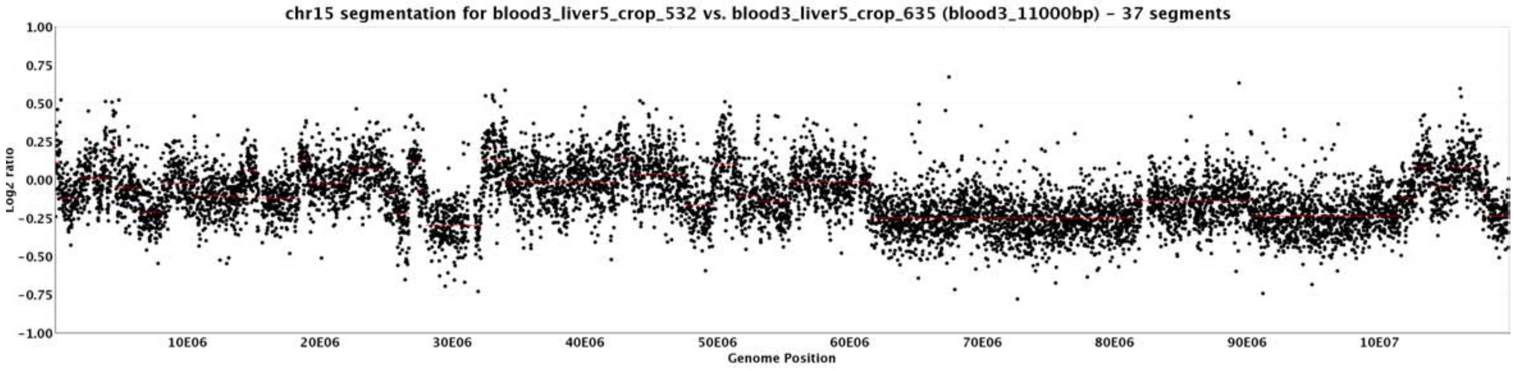
chr15 segmentation for blood3_liver5_crop_532 vs. blood3_liver5_crop_635 (blood3_11000bp) - 37 segments
Log2 ratio
Genome Position

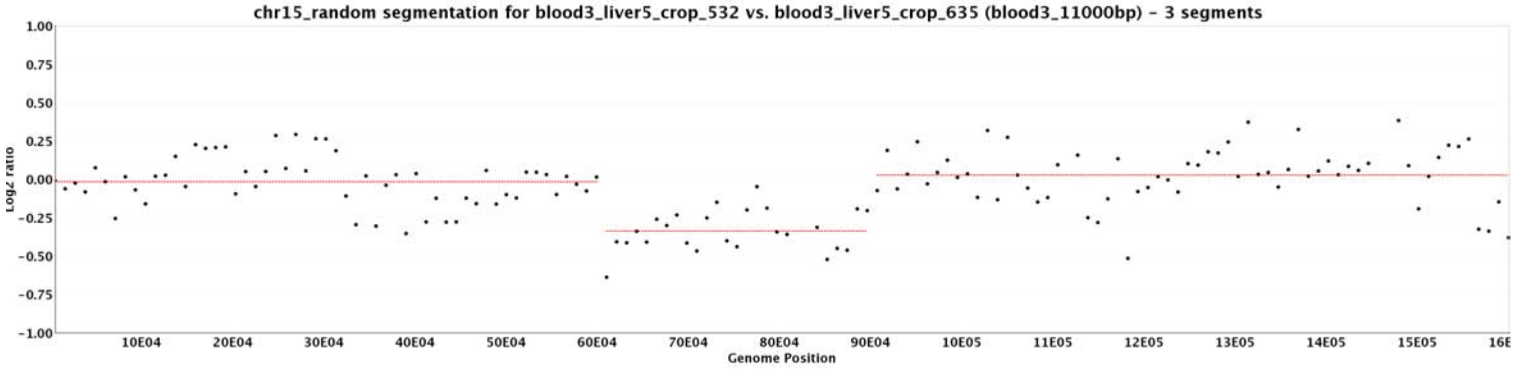
chr15_random segmentation for blood3_liver5_crop_532 vs. blood3_liver5_crop_635 (blood3_11000bp) - 3 segments
Log2 ratio
Genome Position

chr16 segmentation for blood3_liver5_crop_532 vs. blood3_liver5_crop_635 (blood3_11000bp) - 49 segments

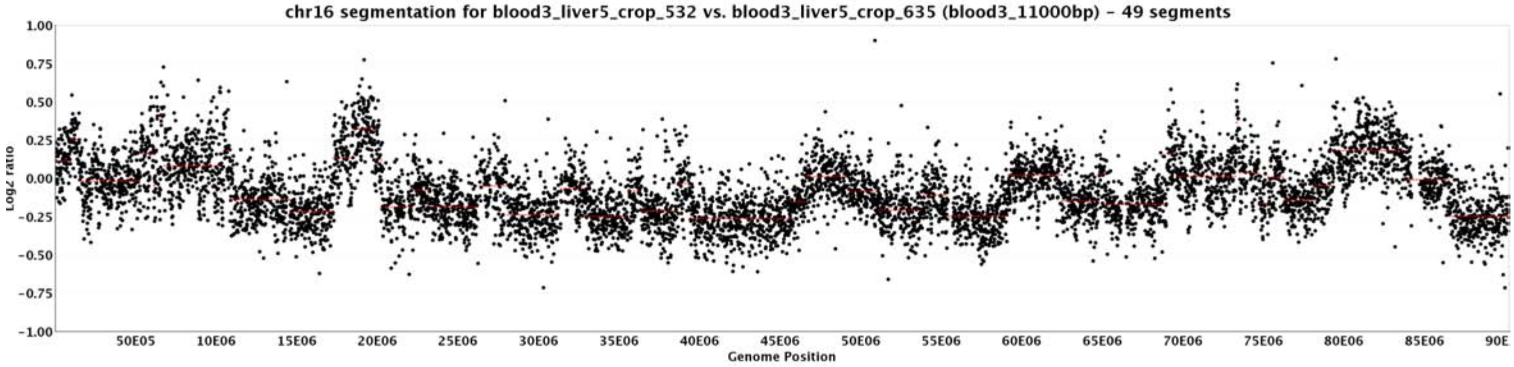

chr16_random segmentation for blood3_liver5_crop_532 vs. blood3_liver5_crop_635 (blood3_11000bp) - 5 segments

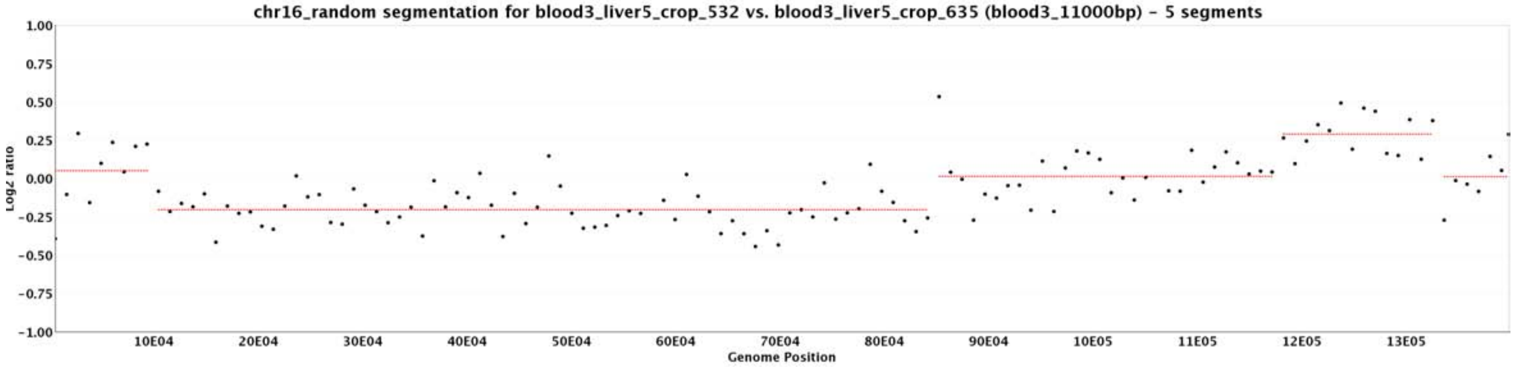

chr17 segmentation for blood3_liver5_crop_532 vs. blood3_liver5_crop_635 (blood3_11000bp) - 41 segments

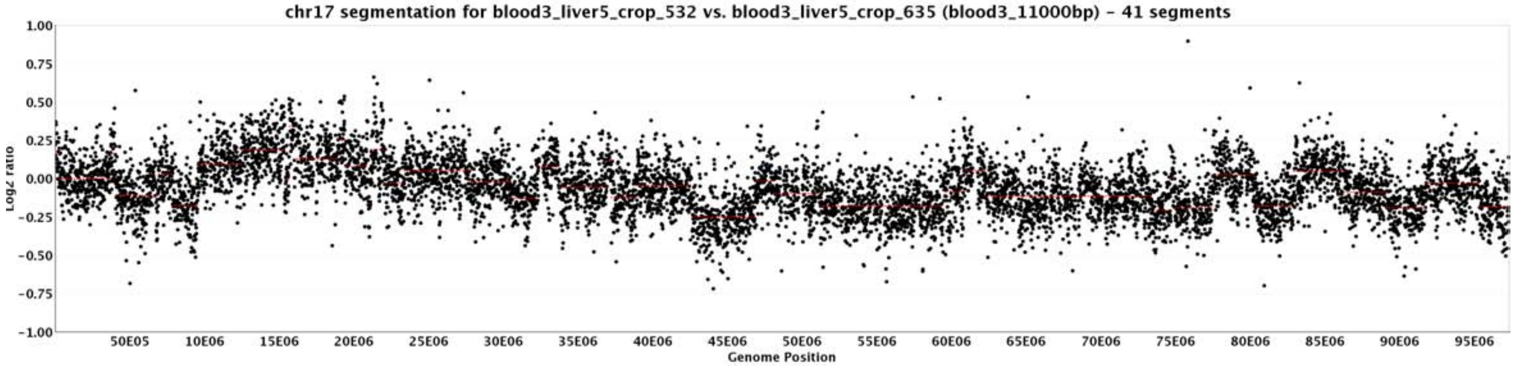

chr17_random segmentation for blood3_liver5_crop_532 vs. blood3_liver5_crop_635 (blood3_11000bp) - 1 segment

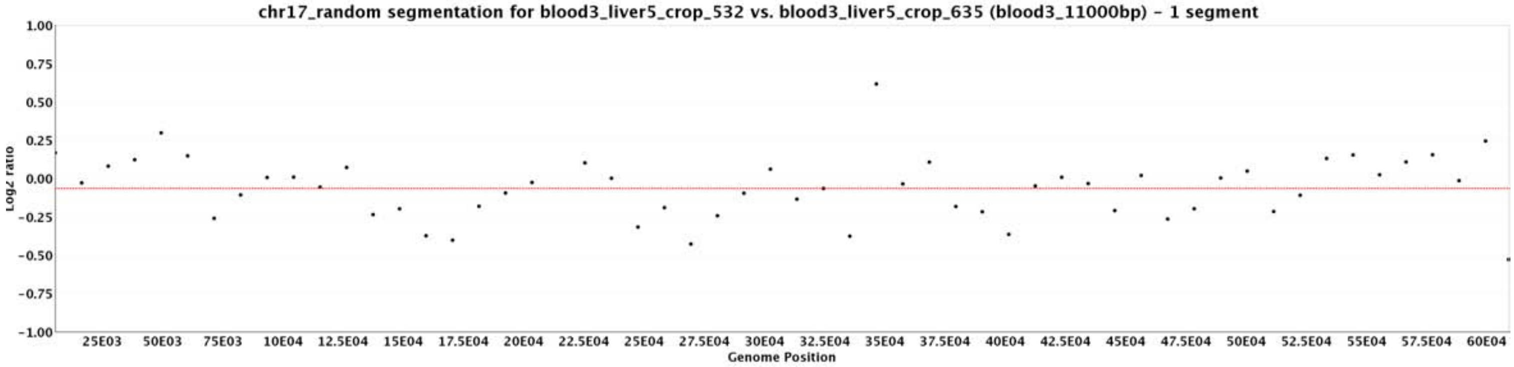

chr18 segmentation for blood3_liver5_crop_532 vs. blood3_liver5_crop_635 (blood3_11000bp) - 37 segments

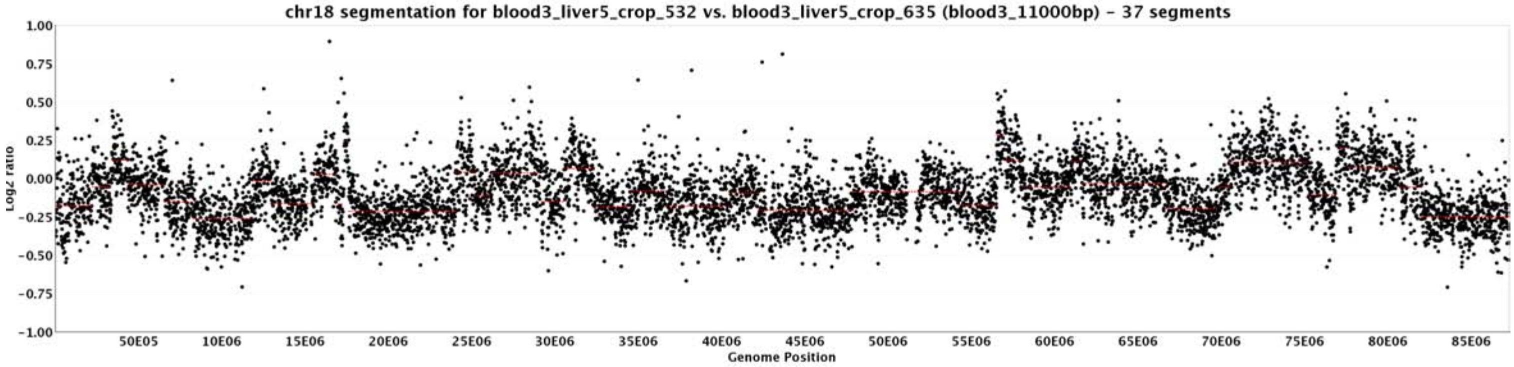

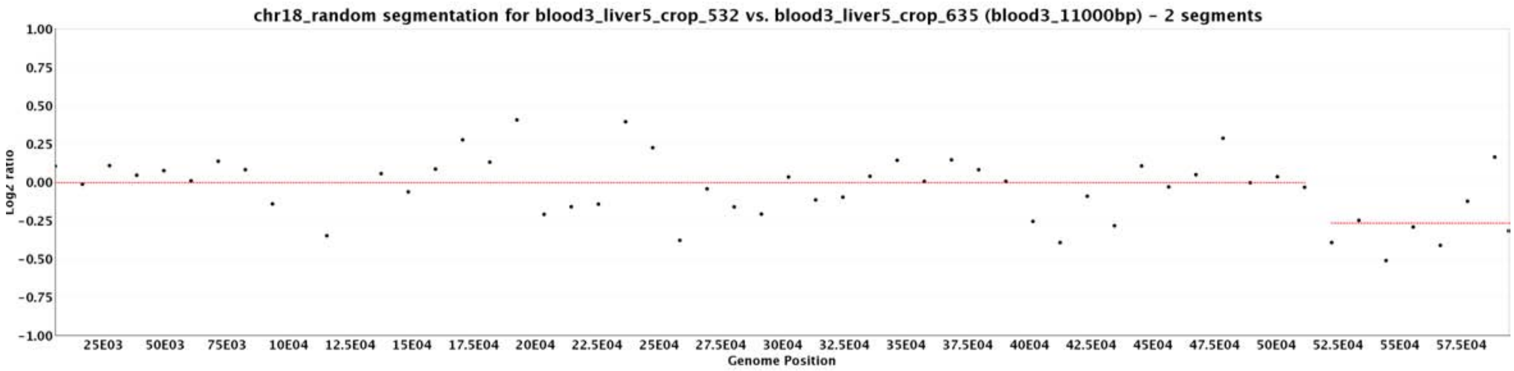
chr18_random segmentation for blood3_liver5_crop_532 vs. blood3_liver5_crop_635 (blood3_11000bp) - 2 segments
Log2 ratio
1.00
0.75
0.50
0.25
0.00
-0.25
-0.50
-0.75
-1.00
25E03 50E03 75E03 10E04 12.5E04 15E04 17.5E04 20E04 22.5E04 25E04 27.5E04 30E04 32.5E04 35E04 37.5E04 40E04 42.5E04 45E04 47.5E04 50E04 52.5E04 55E04 57.5E04
Genome Position

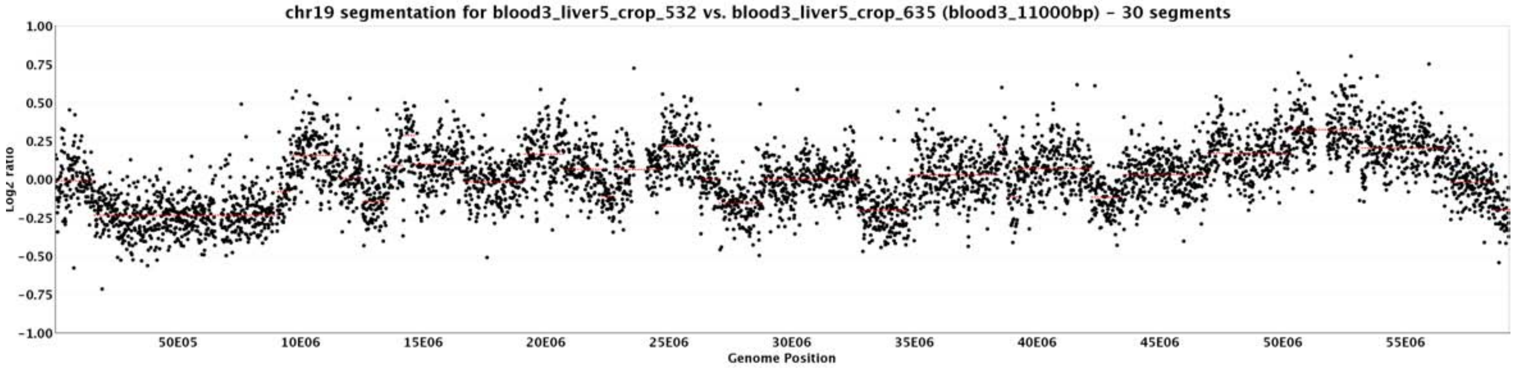
chr19 segmentation for blood3_liver5_crop_532 vs. blood3_liver5_crop_635 (blood3_11000bp) - 30 segments
Log2 ratio
1.00
0.75
0.50
0.25
0.00
-0.25
-0.50
-0.75
-1.00
50E05 10E06 15E06 20E06 25E06 30E06 35E06 40E06 45E06 50E06 55E06
Genome Position

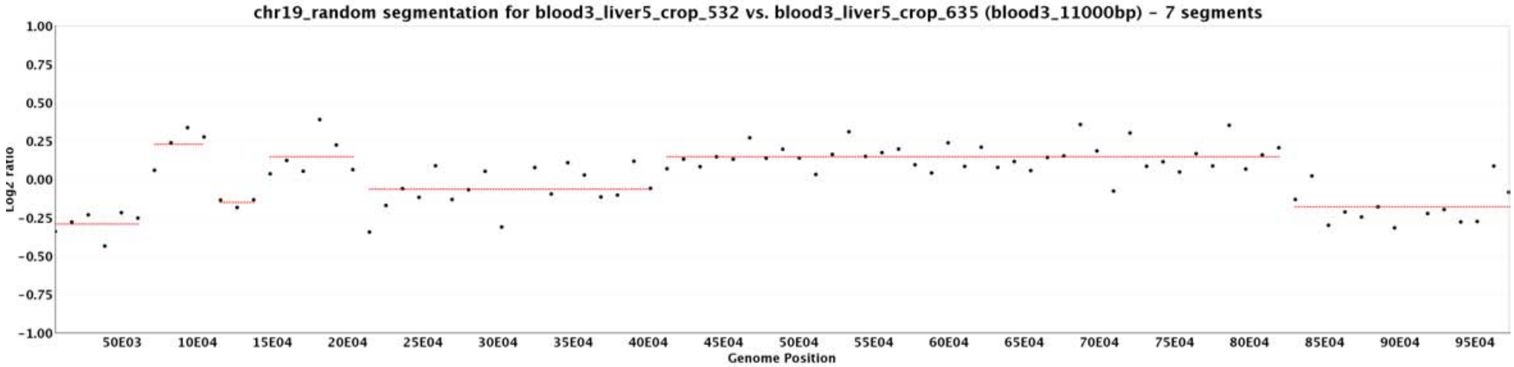
chr19_random segmentation for blood3_liver5_crop_532 vs. blood3_liver5_crop_635 (blood3_11000bp) - 7 segments
Log2 ratio
1.00
0.75
0.50
0.25
0.00
-0.25
-0.50
-0.75
-1.00
50E03 10E04 15E04 20E04 25E04 30E04 35E04 40E04 45E04 50E04 55E04 60E04 65E04 70E04 75E04 80E04 85E04 90E04 95E04
Genome Position

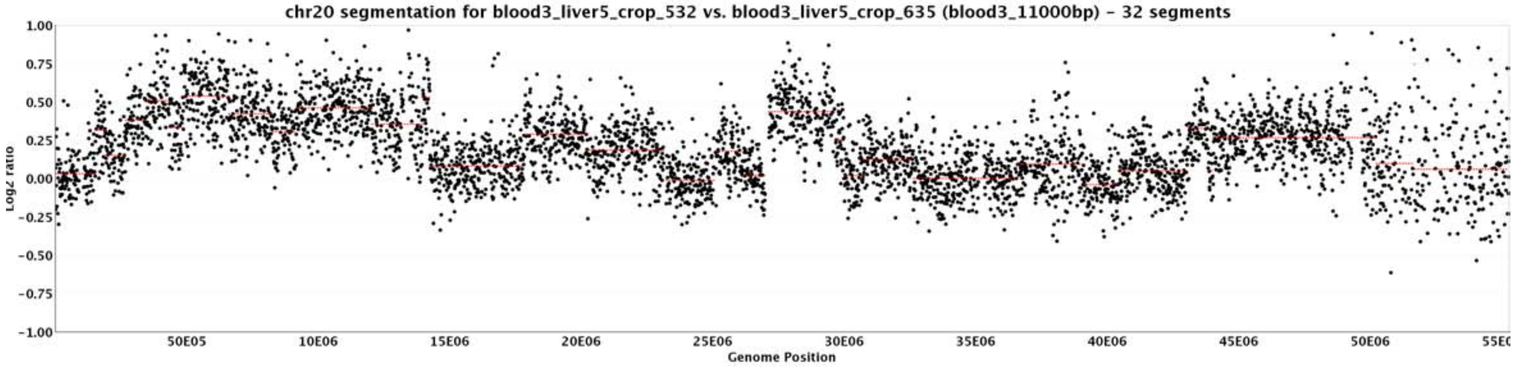
chr20 segmentation for blood3_liver5_crop_532 vs. blood3_liver5_crop_635 (blood3_11000bp) - 32 segments
Log2 ratio
1.00
0.75
0.50
0.25
0.00
-0.25
-0.50
-0.75
-1.00
50E05 10E06 15E06 20E06 25E06 30E06 35E06 40E06 45E06 50E06
Genome Position

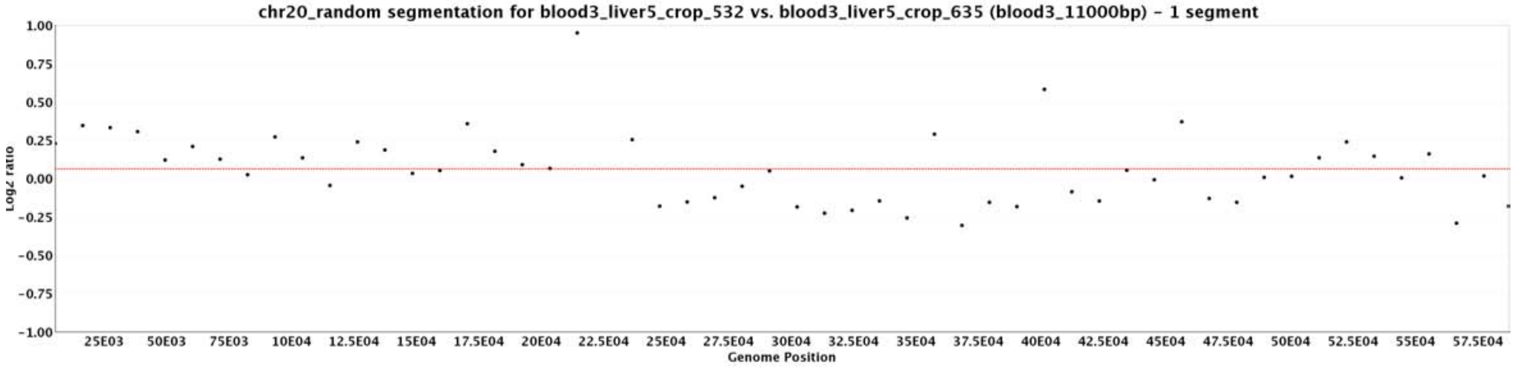
chr20_random segmentation for blood3_liver5_crop_532 vs. blood3_liver5_crop_635 (blood3_11000bp) - 1 segment
Log2 ratio
1.00
0.75
0.50
0.25
0.00
-0.25
-0.50
-0.75
-1.00
25E03 50E03 75E03 10E04 12.5E04 15E04 17.5E04 20E04 22.5E04 25E04 27.5E04 30E04 32.5E04 35E04 37.5E04 40E04 42.5E04 45E04 47.5E04 50E04 52.5E04 55E04 57.5E04
Genome Position

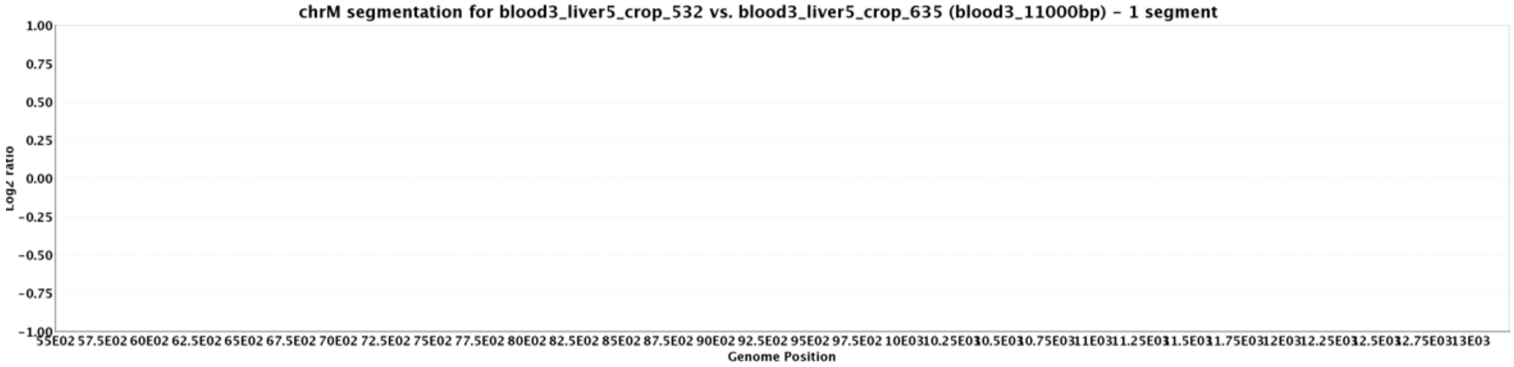
chrM segmentation for blood3_liver5_crop_532 vs. blood3_liver5_crop_635 (blood3_11000bp) - 1 segment
Log2 ratio
Genome Position

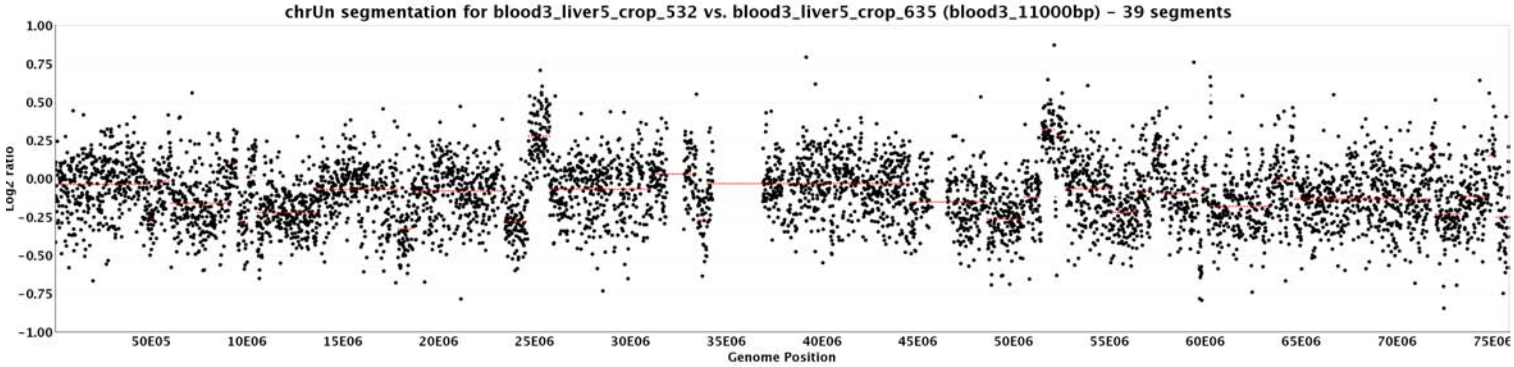
chrUn segmentation for blood3_liver5_crop_532 vs. blood3_liver5_crop_635 (blood3_11000bp) - 39 segments
Log2 ratio
Genome Position

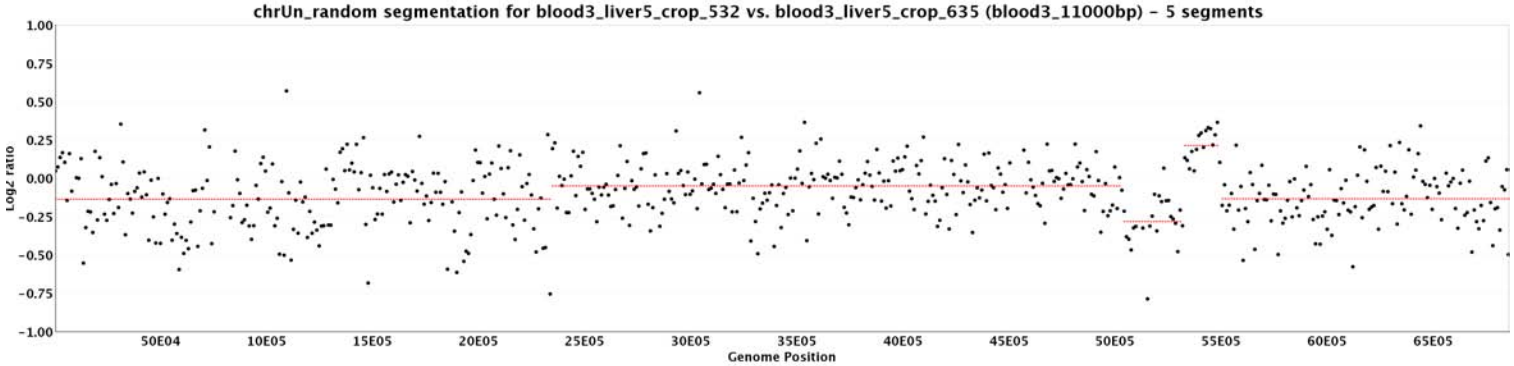
chrUn_random segmentation for blood3_liver5_crop_532 vs. blood3_liver5_crop_635 (blood3_11000bp) - 5 segments
Log2 ratio
Genome Position

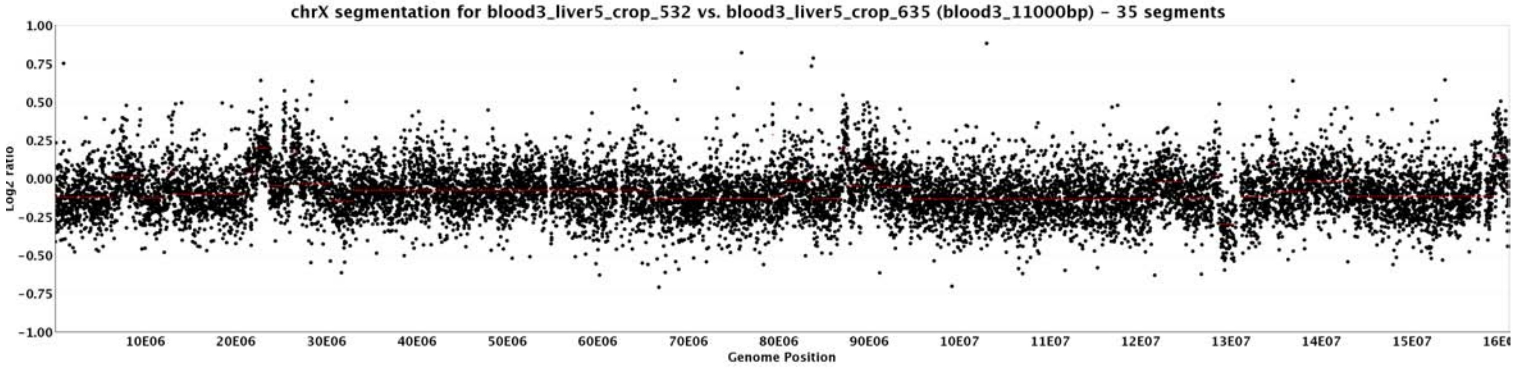
chrX segmentation for blood3_liver5_crop_532 vs. blood3_liver5_crop_635 (blood3_11000bp) - 35 segments
Log2 ratio
Genome Position

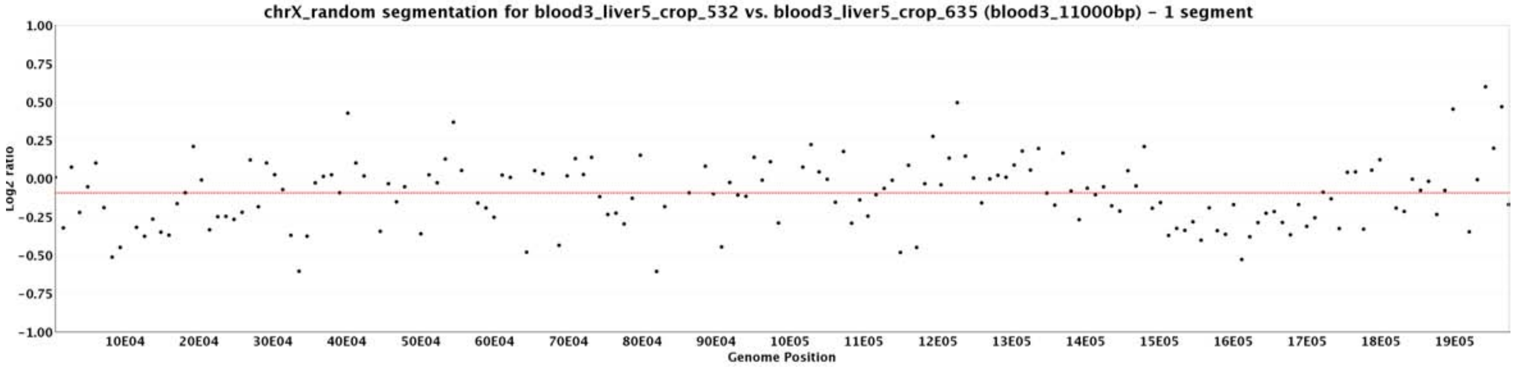
chrX_random segmentation for blood3_liver5_crop_532 vs. blood3_liver5_crop_635 (blood3_11000bp) - 1 segment
Log2 ratio
Genome Position

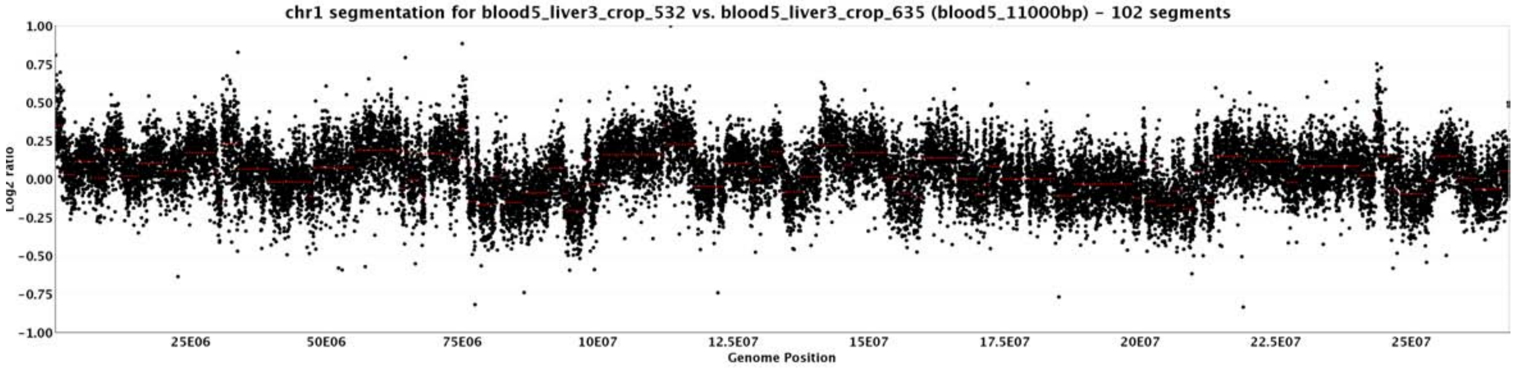
chr1 segmentation for blood5_liver3_crop_532 vs. blood5_liver3_crop_635 (blood5_11000bp) - 102 segments
Log2 ratio
Genome Position
25E06 50E06 75E06 10E07 12.5E07 15E07 17.5E07 20E07 22.5E07 25E07

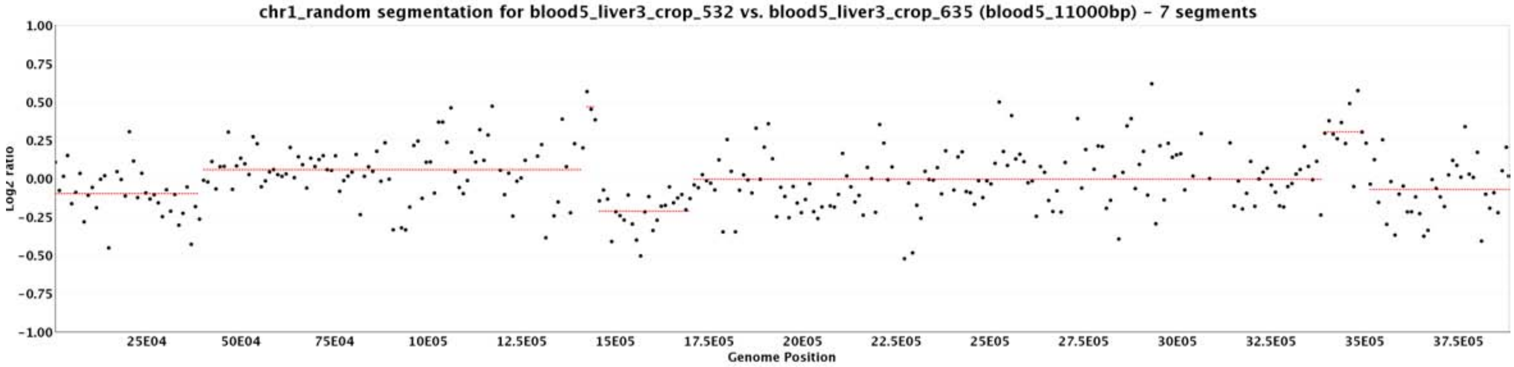
chr1_random segmentation for blood5_liver3_crop_532 vs. blood5_liver3_crop_635 (blood5_11000bp) - 7 segments
Log2 ratio
Genome Position
25E04 50E04 75E04 10E05 12.5E05 15E05 17.5E05 20E05 22.5E05 25E05 27.5E05 30E05 32.5E05 35E05 37.5E05

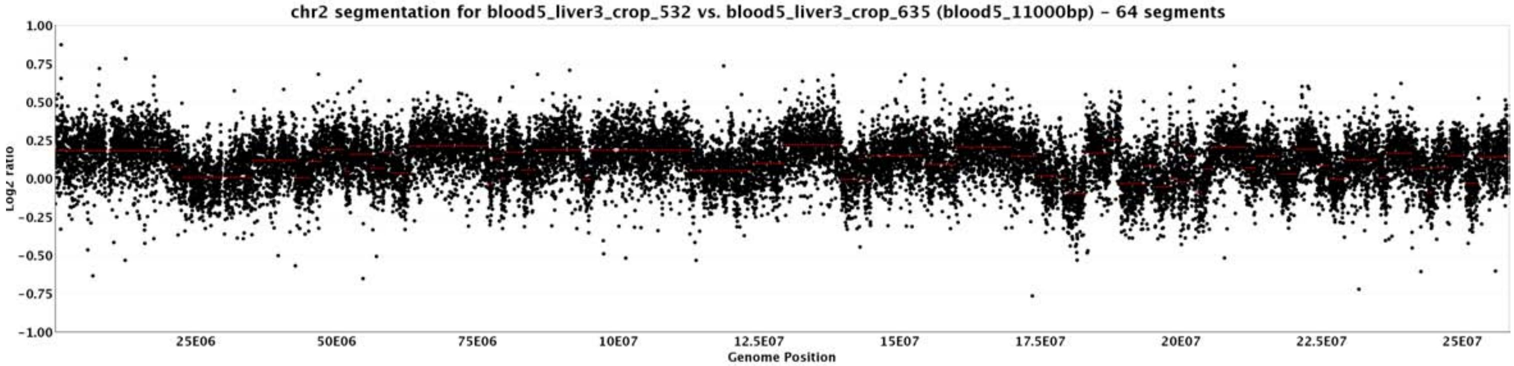
chr2 segmentation for blood5_liver3_crop_532 vs. blood5_liver3_crop_635 (blood5_11000bp) - 64 segments
Log2 ratio
Genome Position
25E06 50E06 75E06 10E07 12.5E07 15E07 17.5E07 20E07 22.5E07 25E07

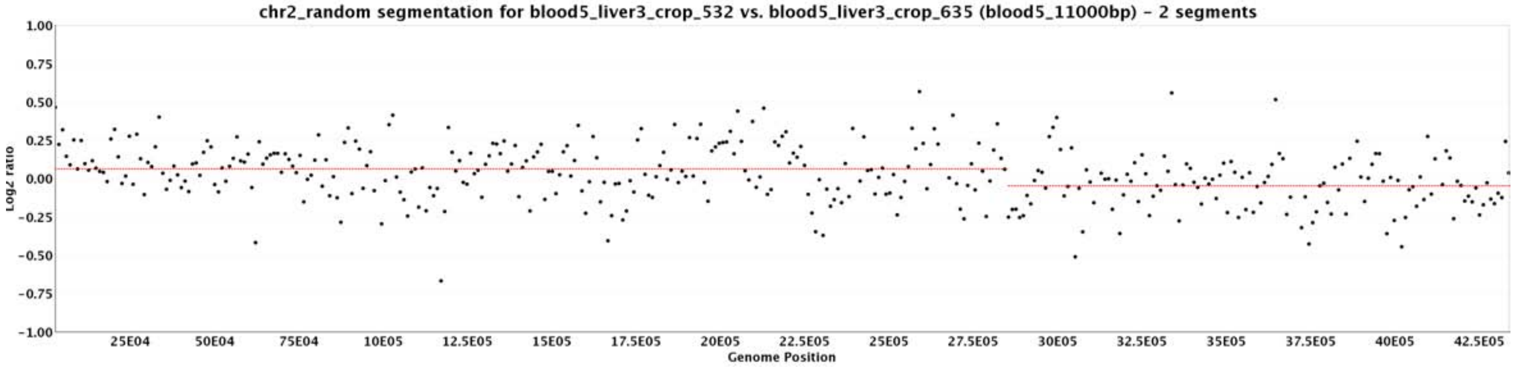
chr2_random segmentation for blood5_liver3_crop_532 vs. blood5_liver3_crop_635 (blood5_11000bp) - 2 segments
Log2 ratio
Genome Position
25E04 50E04 75E04 10E05 12.5E05 15E05 17.5E05 20E05 22.5E05 25E05 27.5E05 30E05 32.5E05 35E05 37.5E05 40E05 42.5E05

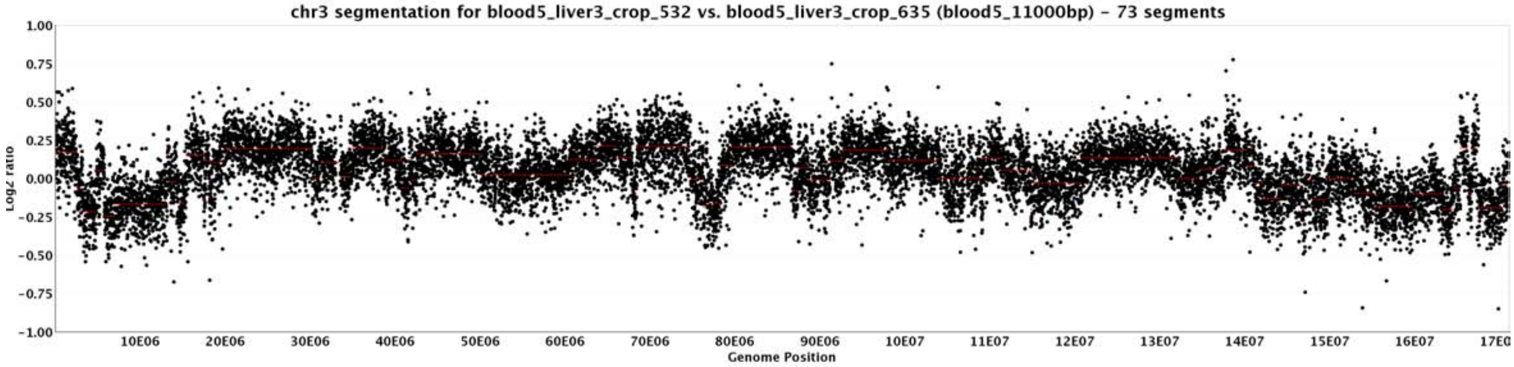
chr3 segmentation for blood5_liver3_crop_532 vs. blood5_liver3_crop_635 (blood5_11000bp) - 73 segments
Log2 ratio
Genome Position
10E06 20E06 30E06 40E06 50E06 60E06 70E06 80E06 90E06 10E07 11E07 12E07 13E07 14E07 15E07 16E07 17E0

chr3_random segmentation for blood5_liver3_crop_532 vs. blood5_liver3_crop_635 (blood5_11000bp) - 8 segments

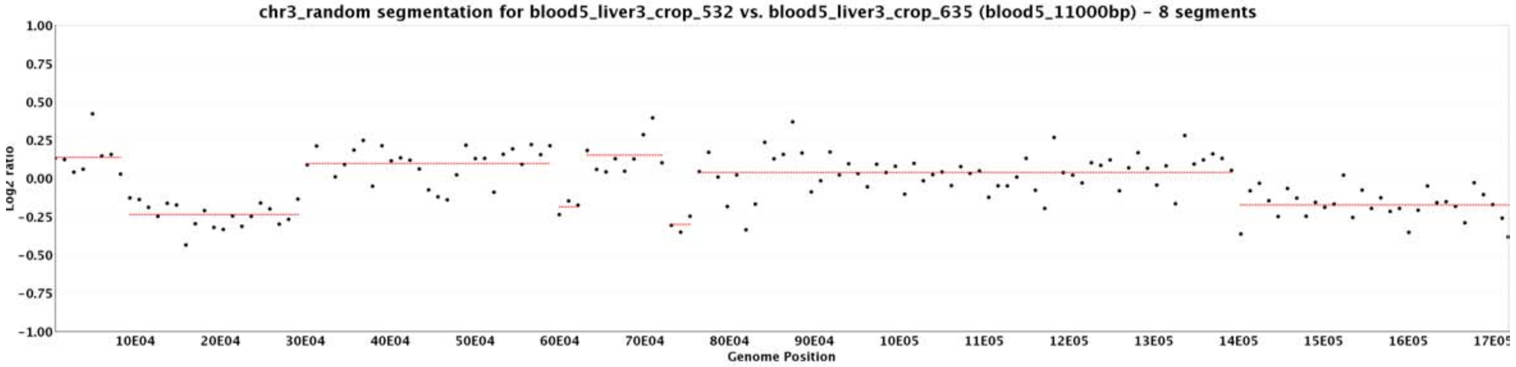

chr4 segmentation for blood5_liver3_crop_532 vs. blood5_liver3_crop_635 (blood5_11000bp) - 66 segments

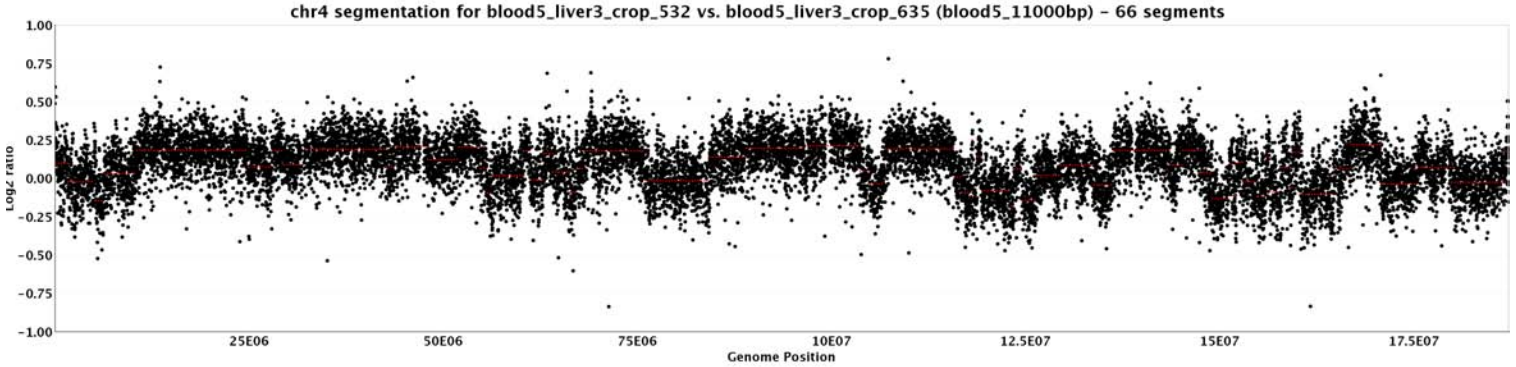

chr4_random segmentation for blood5_liver3_crop_532 vs. blood5_liver3_crop_635 (blood5_11000bp) - 4 segments

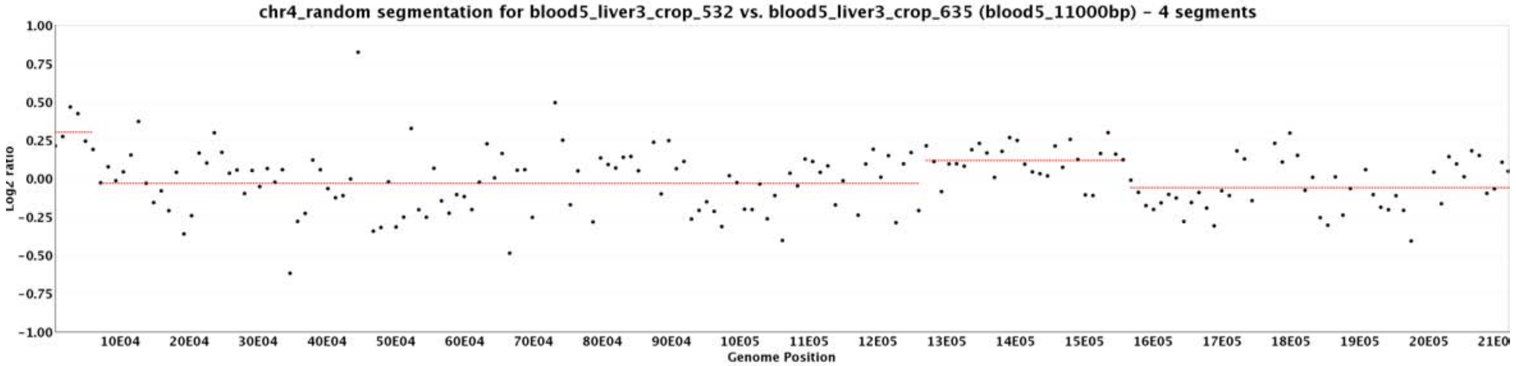

chr5 segmentation for blood5_liver3_crop_532 vs. blood5_liver3_crop_635 (blood5_11000bp) - 54 segments

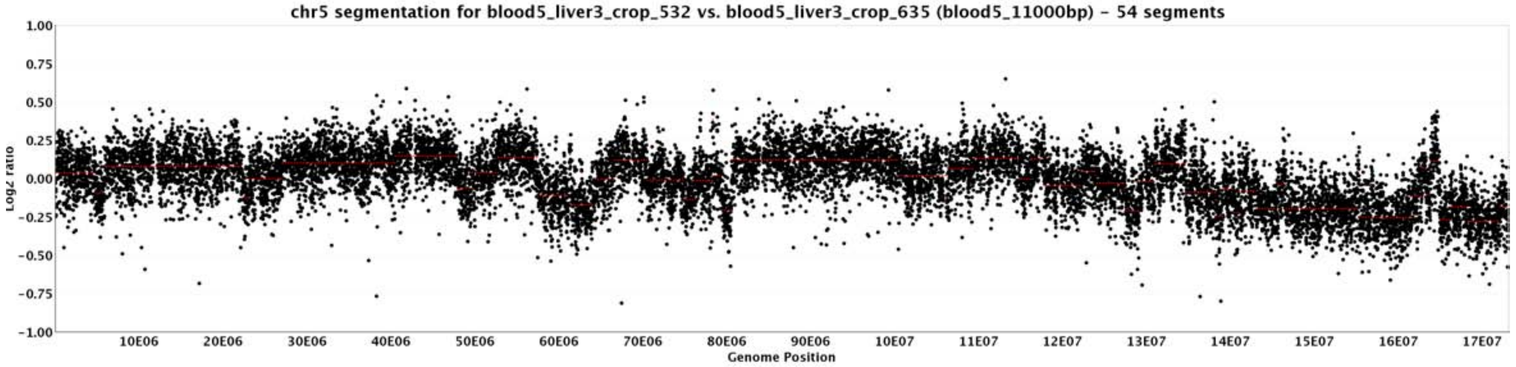

chr5_random segmentation for blood5_liver3_crop_532 vs. blood5_liver3_crop_635 (blood5_11000bp) - 2 segments

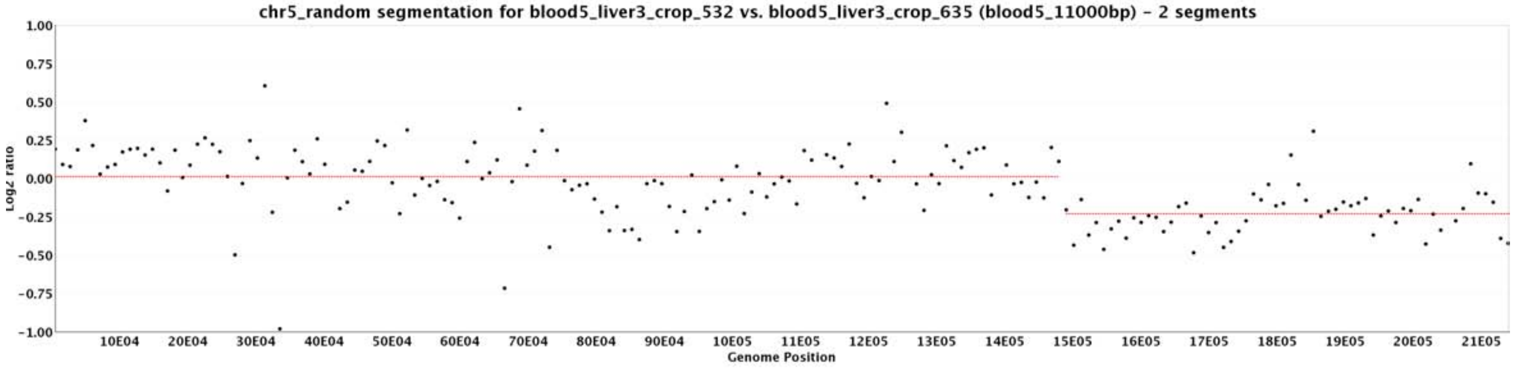

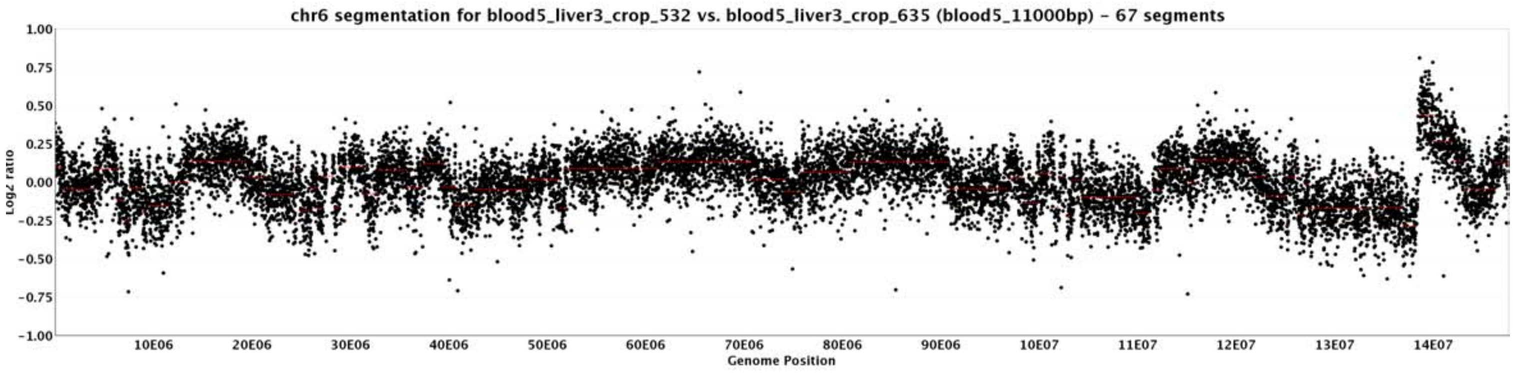
chr6 segmentation for blood5_liver3_crop_532 vs. blood5_liver3_crop_635 (blood5_11000bp) - 67 segments
Log2 ratio
1.00
0.75
0.50
0.25
0.00
-0.25
-0.50
-0.75
-1.00
10E06
20E06
30E06
40E06
50E06
60E06
70E06
80E06
90E06
10E07
11E07
12E07
13E07
14E07
Genome Position

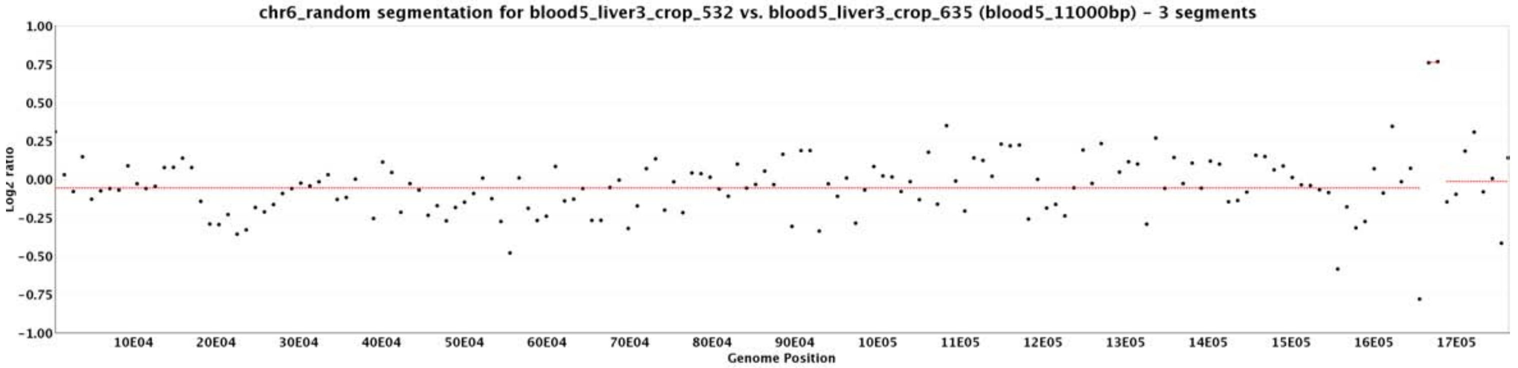
chr6_random segmentation for blood5_liver3_crop_532 vs. blood5_liver3_crop_635 (blood5_11000bp) - 3 segments
Log2 ratio
1.00
0.75
0.50
0.25
0.00
-0.25
-0.50
-0.75
-1.00
10E04
20E04
30E04
40E04
50E04
60E04
70E04
80E04
90E04
10E05
11E05
12E05
13E05
14E05
15E05
16E05
17E05
Genome Position

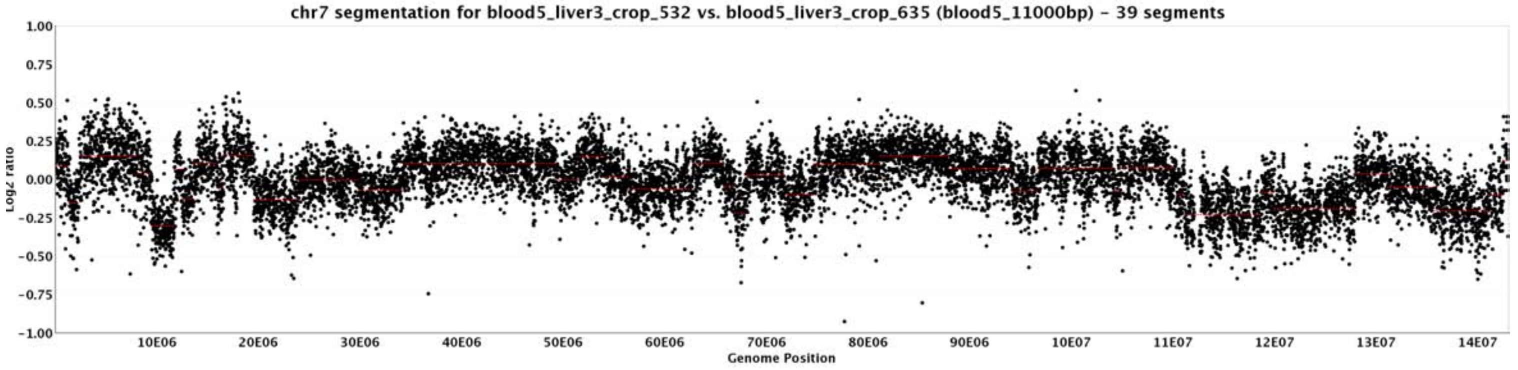
chr7 segmentation for blood5_liver3_crop_532 vs. blood5_liver3_crop_635 (blood5_11000bp) - 39 segments
Log2 ratio
1.00
0.75
0.50
0.25
0.00
-0.25
-0.50
-0.75
-1.00
10E06
20E06
30E06
40E06
50E06
60E06
70E06
80E06
90E06
10E07
11E07
12E07
13E07
14E07
Genome Position

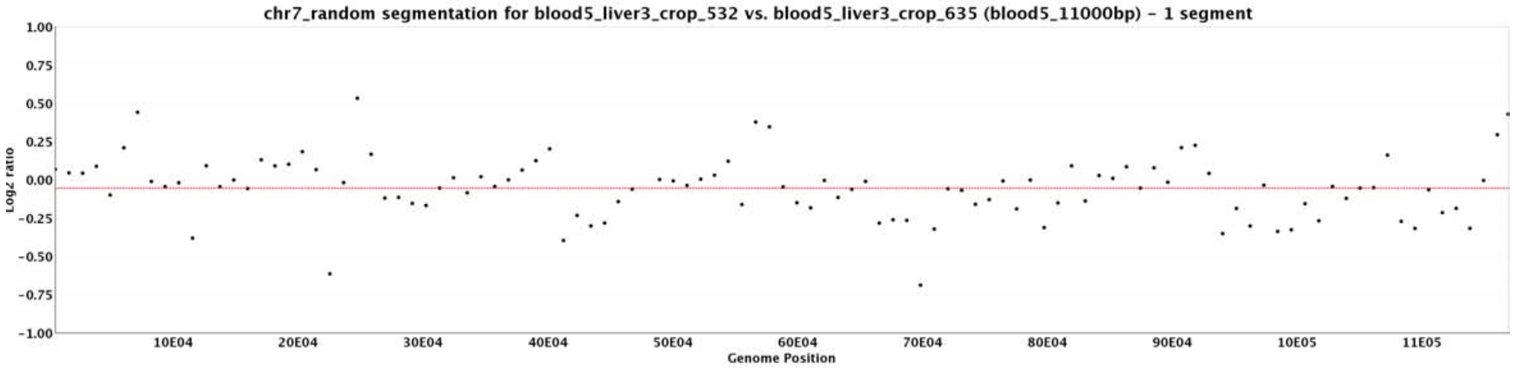
chr7_random segmentation for blood5_liver3_crop_532 vs. blood5_liver3_crop_635 (blood5_11000bp) - 1 segment
Log2 ratio
1.00
0.75
0.50
0.25
0.00
-0.25
-0.50
-0.75
-1.00
10E04
20E04
30E04
40E04
50E04
60E04
70E04
80E04
90E04
10E05
11E05
Genome Position

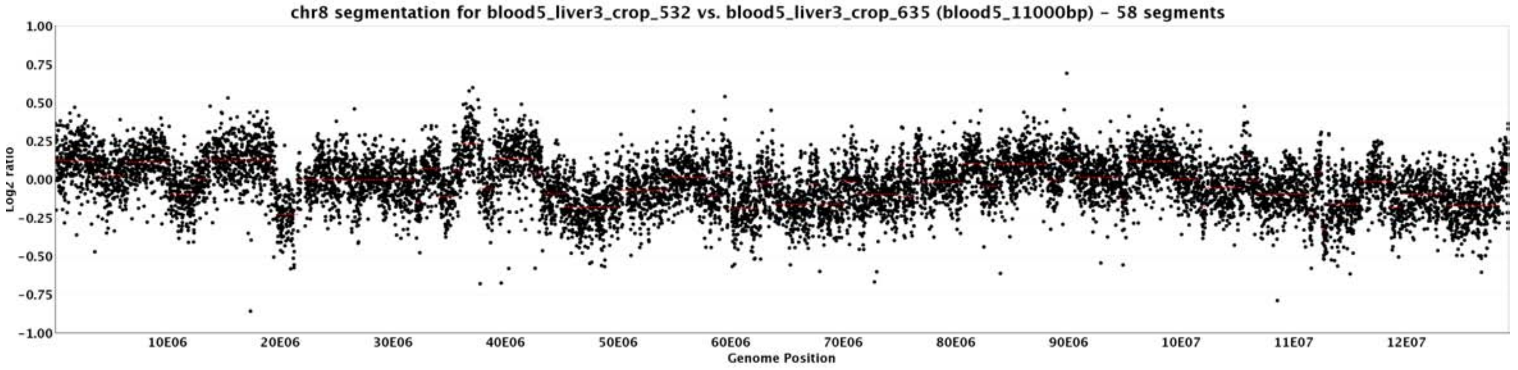
chr8 segmentation for blood5_liver3_crop_532 vs. blood5_liver3_crop_635 (blood5_11000bp) - 58 segments
Log2 ratio
1.00
0.75
0.50
0.25
0.00
-0.25
-0.50
-0.75
-1.00
10E06
20E06
30E06
40E06
50E06
60E06
70E06
80E06
90E06
10E07
11E07
12E07
Genome Position

chr8_random segmentation for blood5_liver3_crop_532 vs. blood5_liver3_crop_635 (blood5_11000bp) - 1 segment

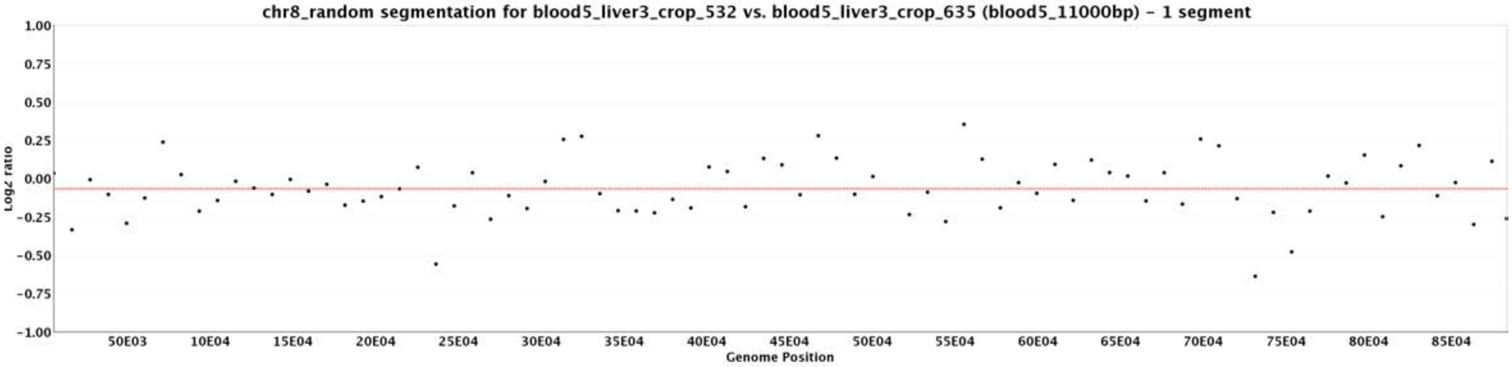

chr9 segmentation for blood5_liver3_crop_532 vs. blood5_liver3_crop_635 (blood5_11000bp) - 40 segments

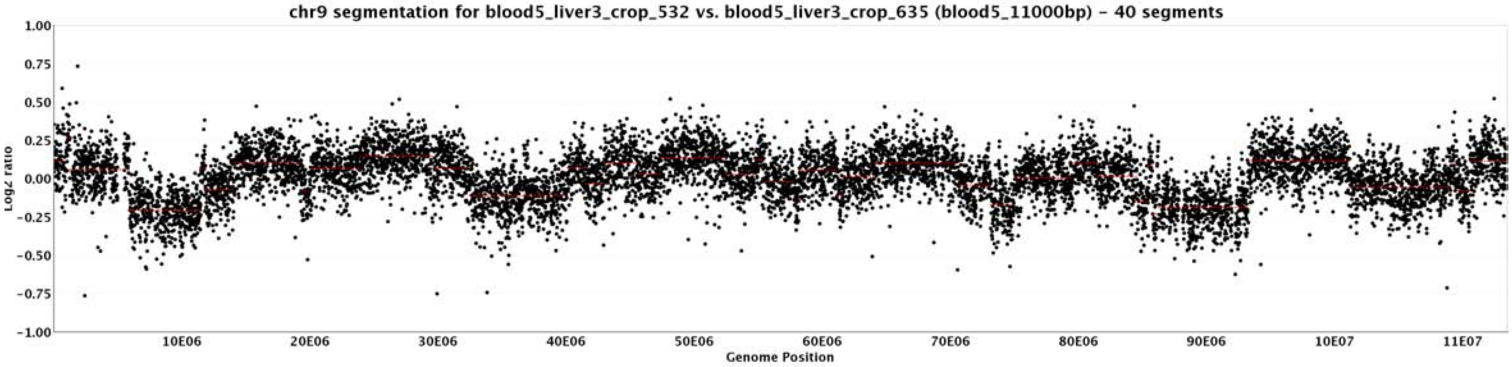

chr9_random segmentation for blood5_liver3_crop_532 vs. blood5_liver3_crop_635 (blood5_11000bp) - 2 segments

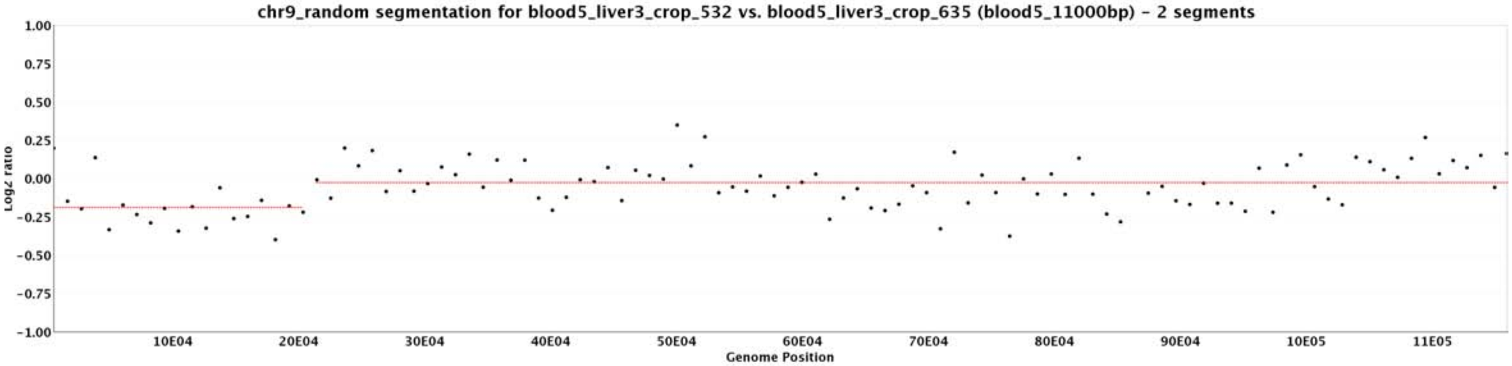

chr10 segmentation for blood5_liver3_crop_532 vs. blood5_liver3_crop_635 (blood5_11000bp) - 67 segments

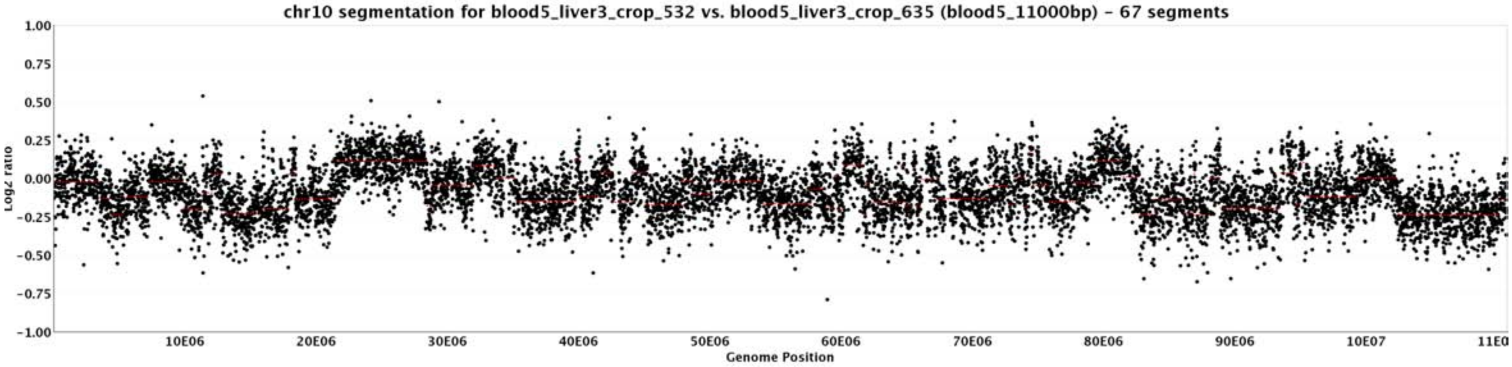

chr10_random segmentation for blood5_liver3_crop_532 vs. blood5_liver3_crop_635 (blood5_11000bp) - 37 segments

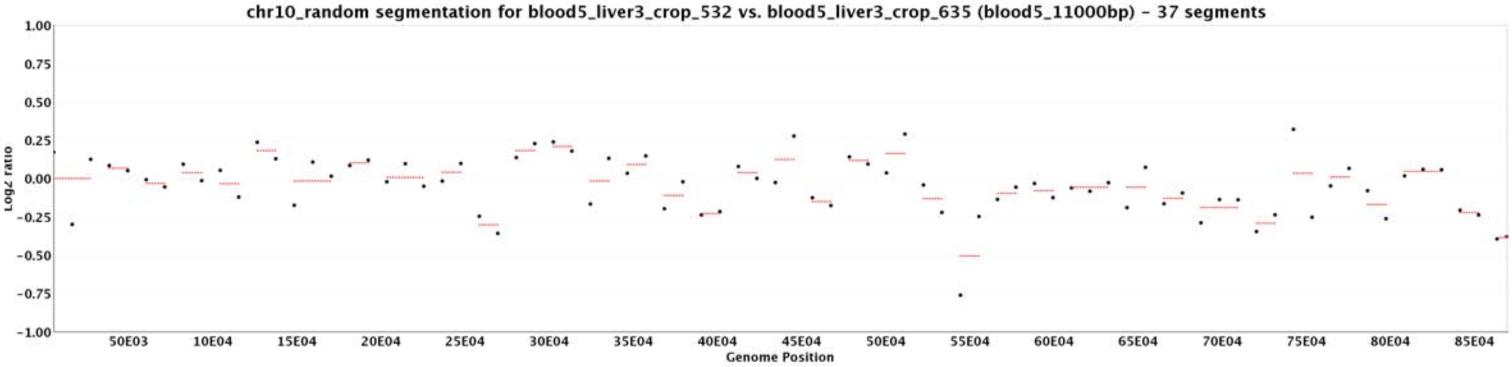

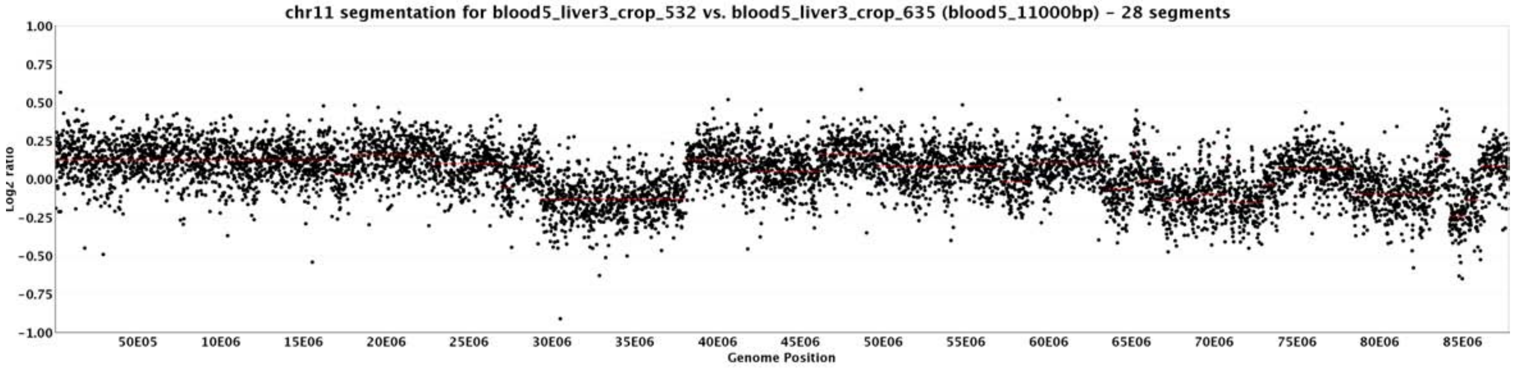
chr11 segmentation for blood5_liver3_crop_532 vs. blood5_liver3_crop_635 (blood5_11000bp) - 28 segments
Log2 ratio
Genome Position

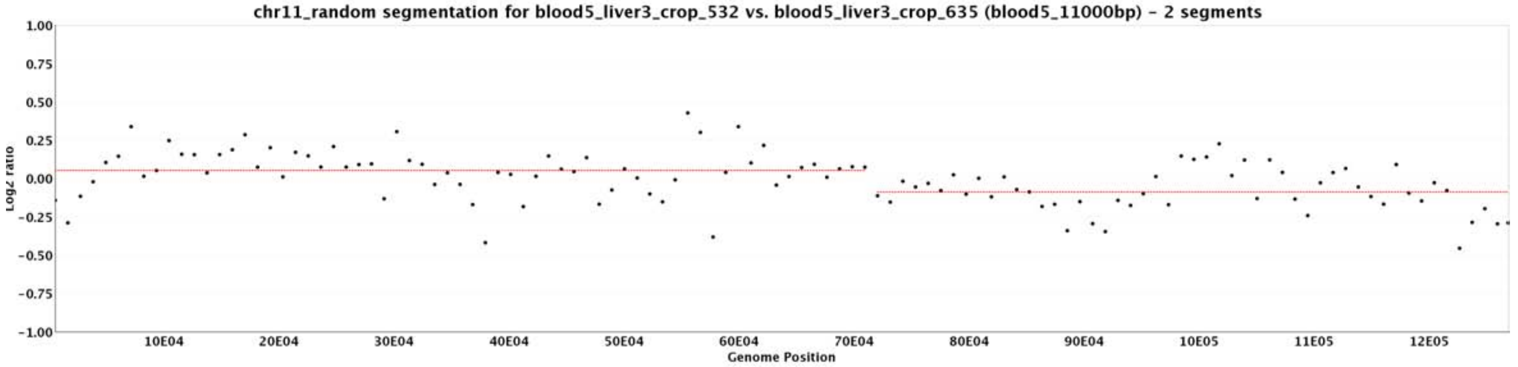
chr11_random segmentation for blood5_liver3_crop_532 vs. blood5_liver3_crop_635 (blood5_11000bp) - 2 segments
Log2 ratio
Genome Position

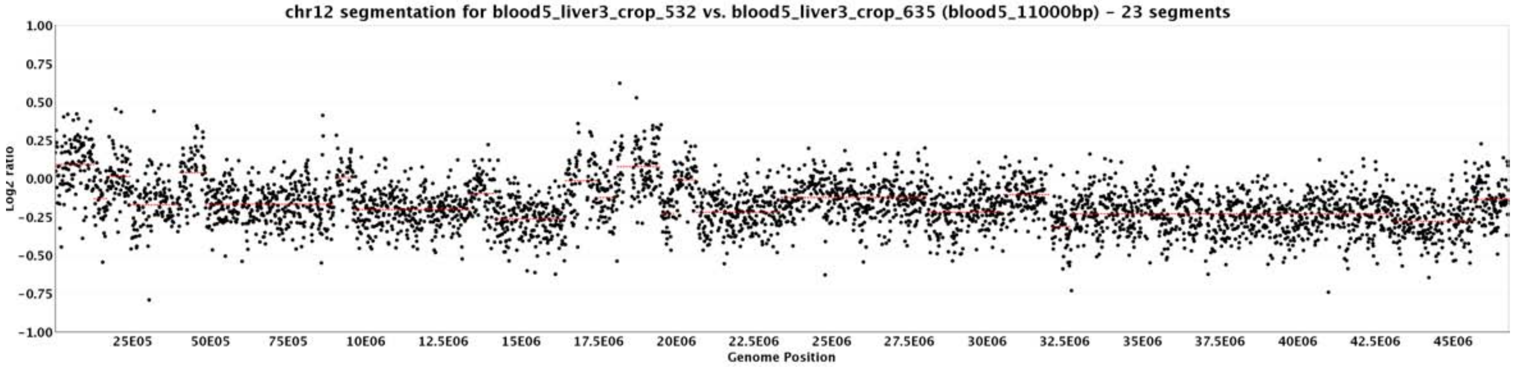
chr12 segmentation for blood5_liver3_crop_532 vs. blood5_liver3_crop_635 (blood5_11000bp) - 23 segments
Log2 ratio
Genome Position

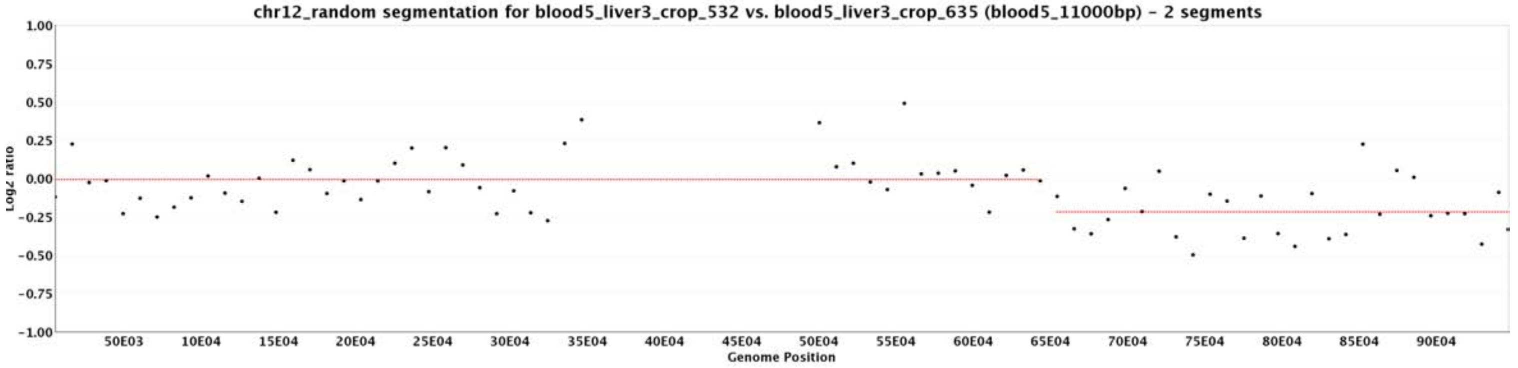
chr12_random segmentation for blood5_liver3_crop_532 vs. blood5_liver3_crop_635 (blood5_11000bp) - 2 segments
Log2 ratio
Genome Position

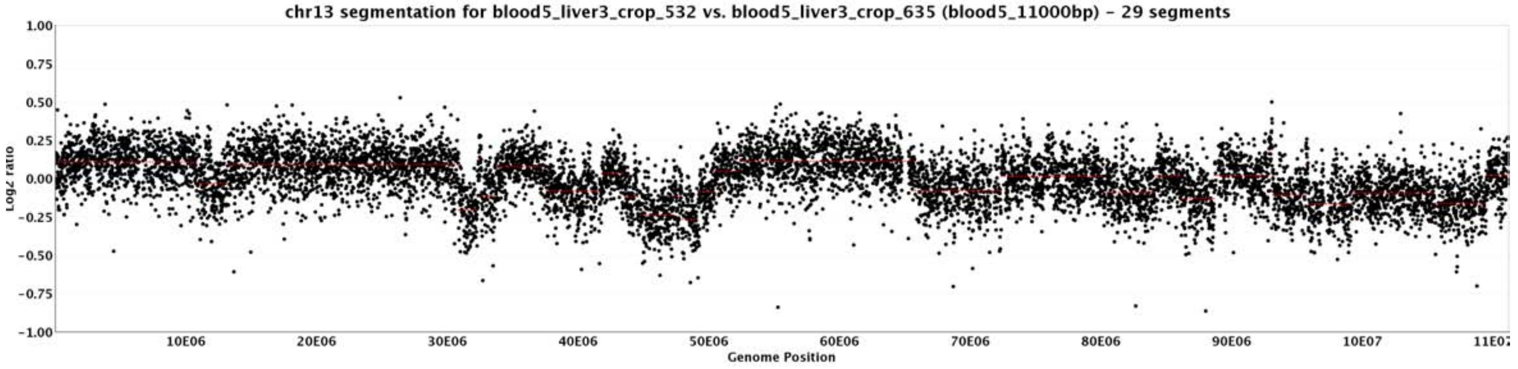
chr13 segmentation for blood5_liver3_crop_532 vs. blood5_liver3_crop_635 (blood5_11000bp) - 29 segments
Log2 ratio
Genome Position

chr13_random segmentation for blood5_liver3_crop_532 vs. blood5_liver3_crop_635 (blood5_11000bp) - 24 segments

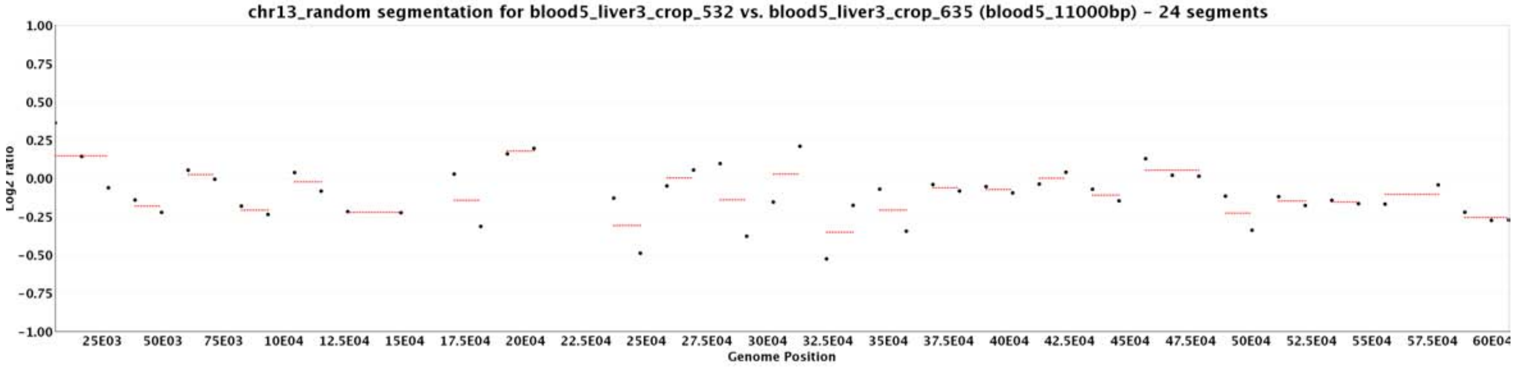

chr14 segmentation for blood5_liver3_crop_532 vs. blood5_liver3_crop_635 (blood5_11000bp) - 31 segments

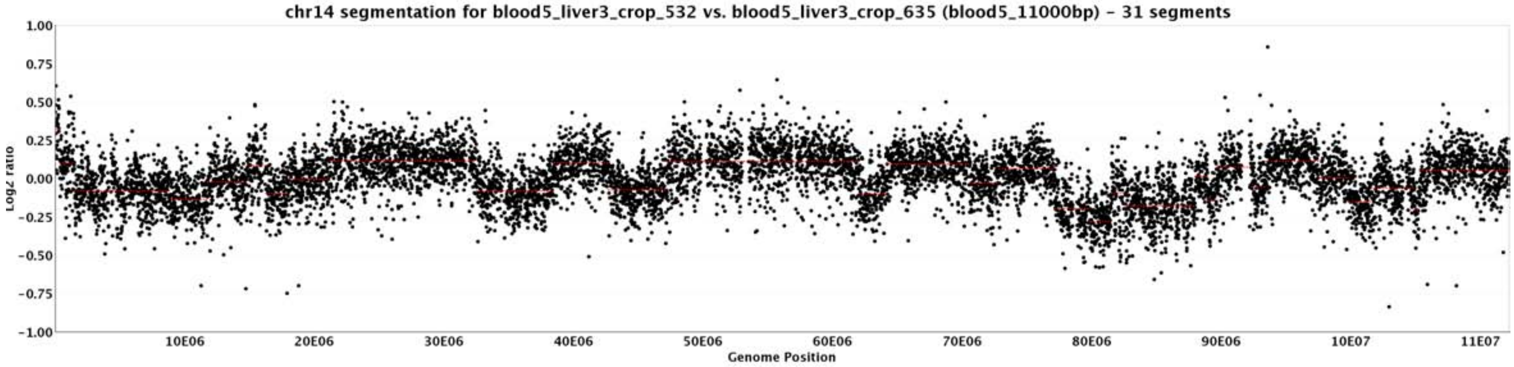

chr14_random segmentation for blood5_liver3_crop_532 vs. blood5_liver3_crop_635 (blood5_11000bp) - 1 segment

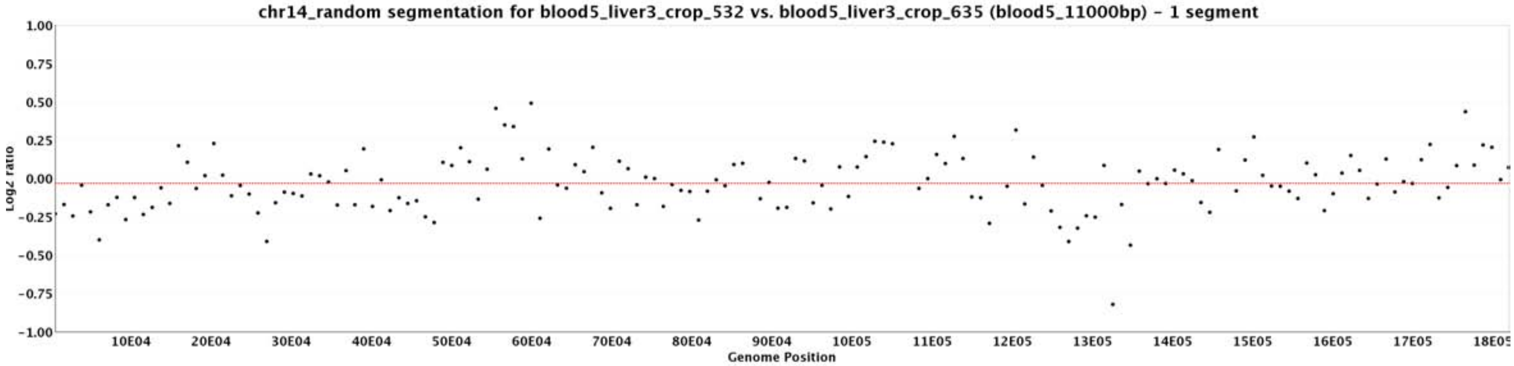

chr15 segmentation for blood5_liver3_crop_532 vs. blood5_liver3_crop_635 (blood5_11000bp) - 29 segments

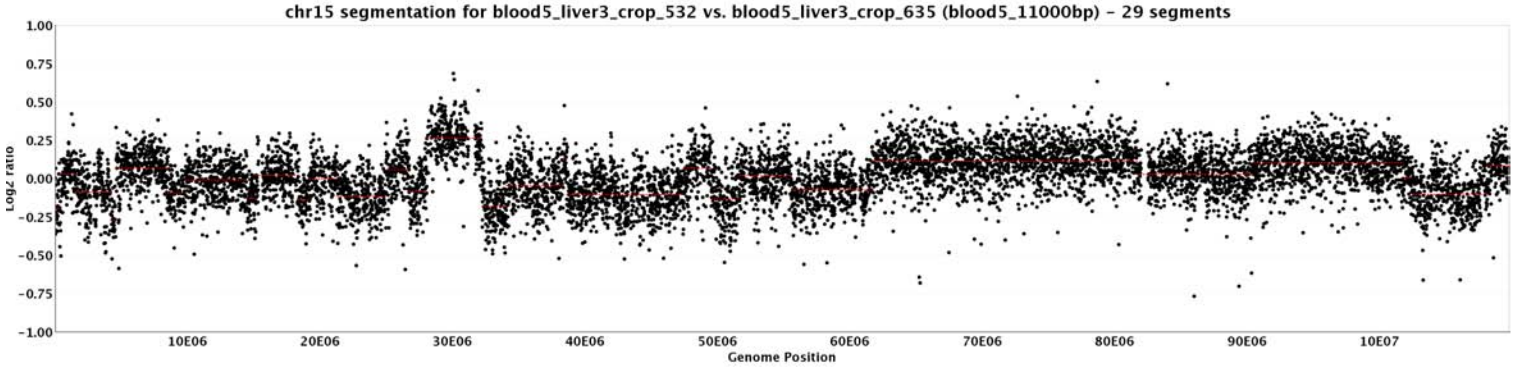

chr15_random segmentation for blood5_liver3_crop_532 vs. blood5_liver3_crop_635 (blood5_11000bp) - 3 segments

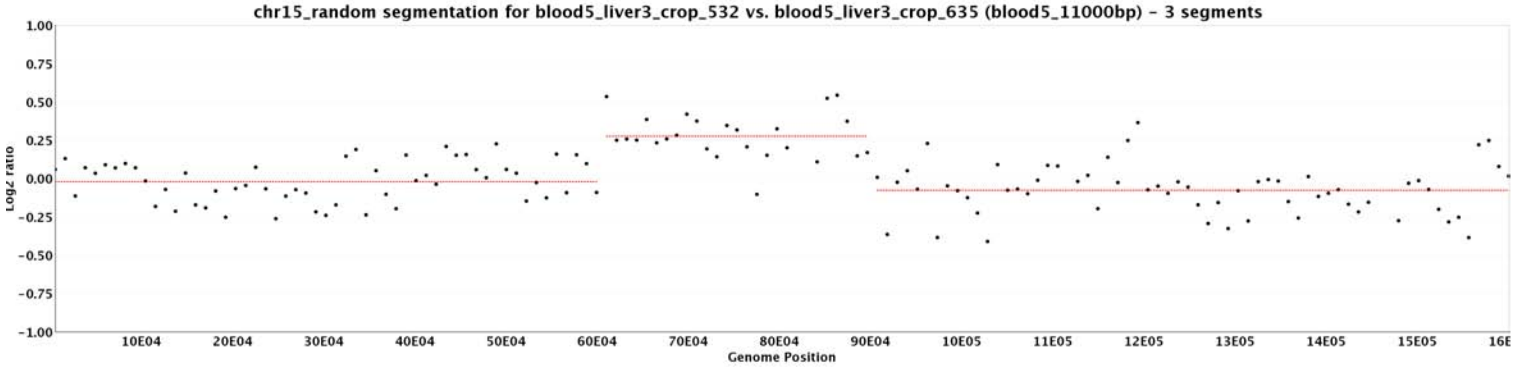

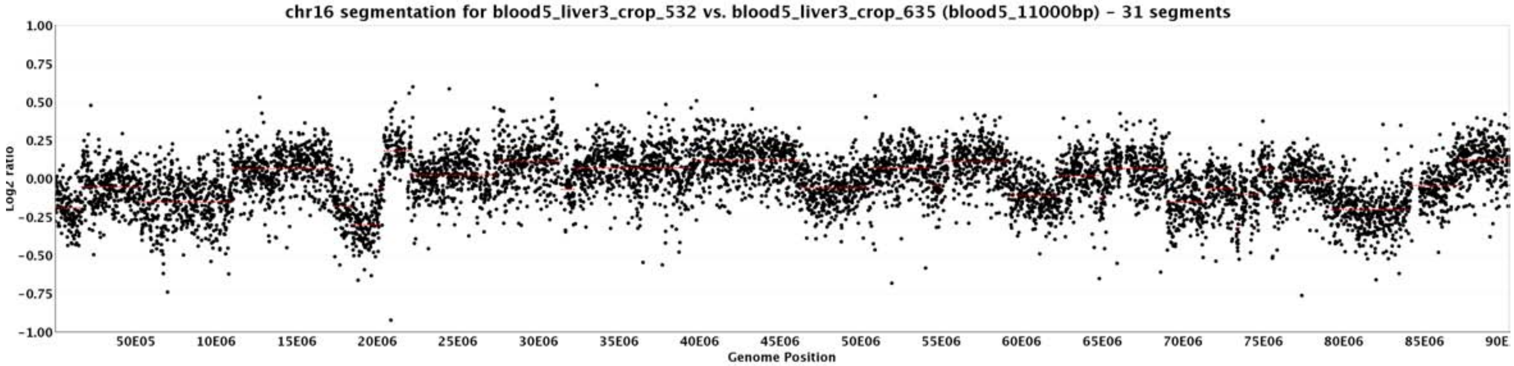
chr16 segmentation for blood5_liver3_crop_532 vs. blood5_liver3_crop_635 (blood5_11000bp) - 31 segments
Log2 ratio
1.00 0.75 0.50 0.25 0.00 -0.25 -0.50 -0.75 -1.00
50E05 10E06 15E06 20E06 25E06 30E06 35E06 40E06 45E06 50E06 55E06 60E06 65E06 70E06 75E06 80E06 85E06 90E
Genome Position

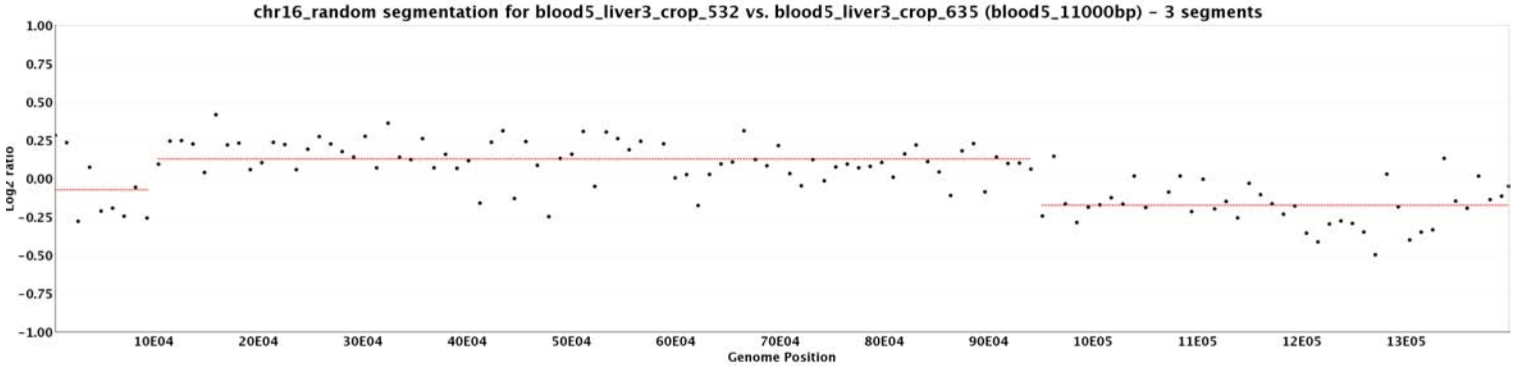
chr16_random segmentation for blood5_liver3_crop_532 vs. blood5_liver3_crop_635 (blood5_11000bp) - 3 segments
Log2 ratio
1.00 0.75 0.50 0.25 0.00 -0.25 -0.50 -0.75 -1.00
10E04 20E04 30E04 40E04 50E04 60E04 70E04 80E04 90E04 10E05 11E05 12E05 13E05
Genome Position

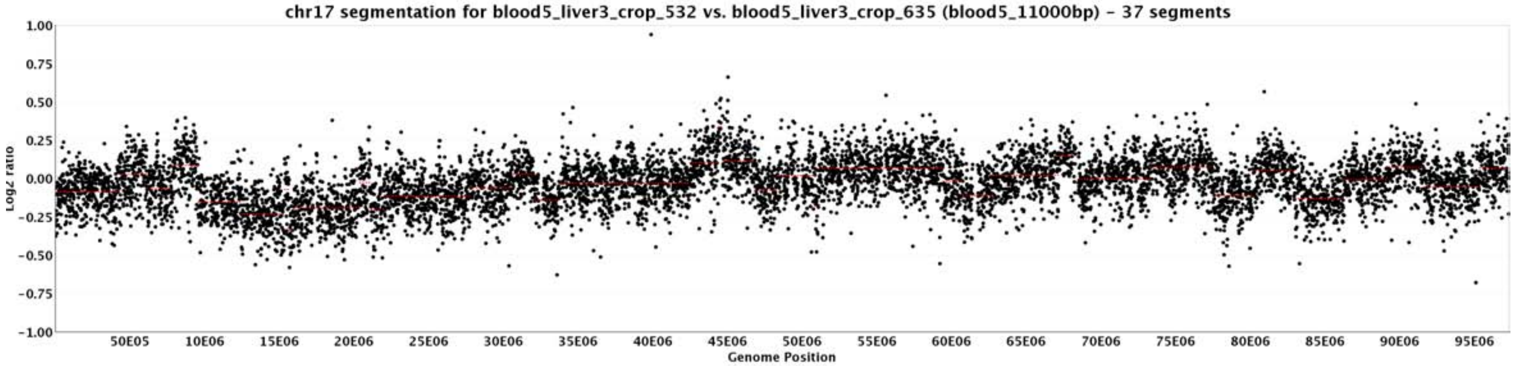
chr17 segmentation for blood5_liver3_crop_532 vs. blood5_liver3_crop_635 (blood5_11000bp) - 37 segments
Log2 ratio
1.00 0.75 0.50 0.25 0.00 -0.25 -0.50 -0.75 -1.00
50E05 10E06 15E06 20E06 25E06 30E06 35E06 40E06 45E06 50E06 55E06 60E06 65E06 70E06 75E06 80E06 85E06 90E06 95E06
Genome Position

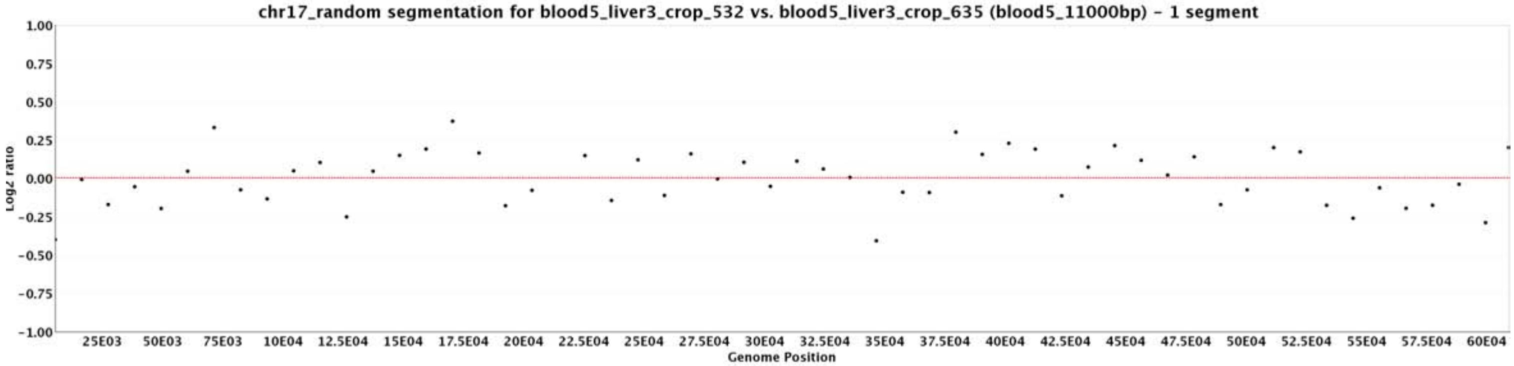
chr17_random segmentation for blood5_liver3_crop_532 vs. blood5_liver3_crop_635 (blood5_11000bp) - 1 segment
Log2 ratio
1.00 0.75 0.50 0.25 0.00 -0.25 -0.50 -0.75 -1.00
25E03 50E03 75E03 10E04 12.5E04 15E04 17.5E04 20E04 22.5E04 25E04 27.5E04 30E04 32.5E04 35E04 37.5E04 40E04 42.5E04 45E04 47.5E04 50E04 52.5E04 55E04 57.5E04 60E04
Genome Position

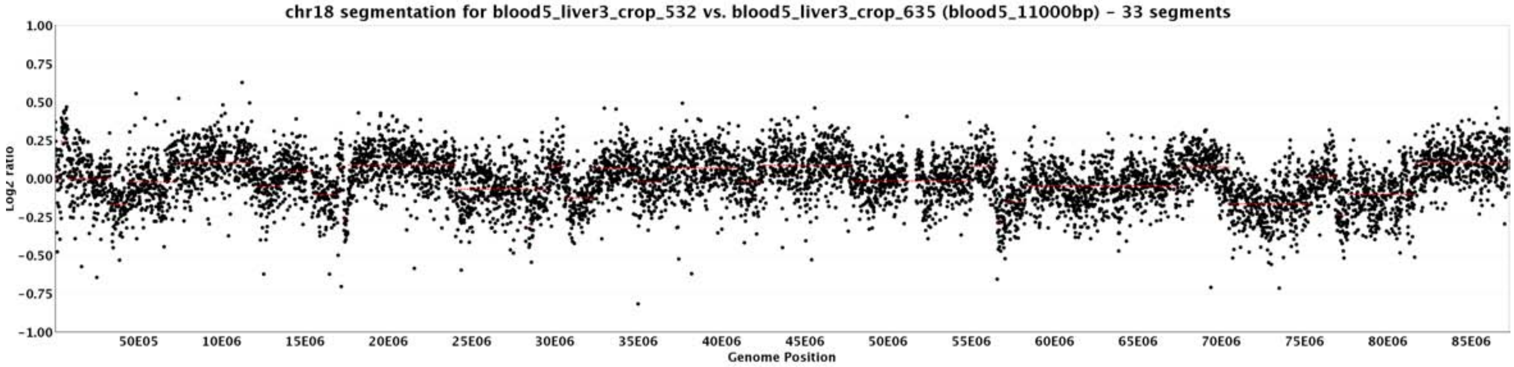
chr18 segmentation for blood5_liver3_crop_532 vs. blood5_liver3_crop_635 (blood5_11000bp) - 33 segments
Log2 ratio
1.00 0.75 0.50 0.25 0.00 -0.25 -0.50 -0.75 -1.00
50E05 10E06 15E06 20E06 25E06 30E06 35E06 40E06 45E06 50E06 55E06 60E06 65E06 70E06 75E06 80E06 85E06
Genome Position

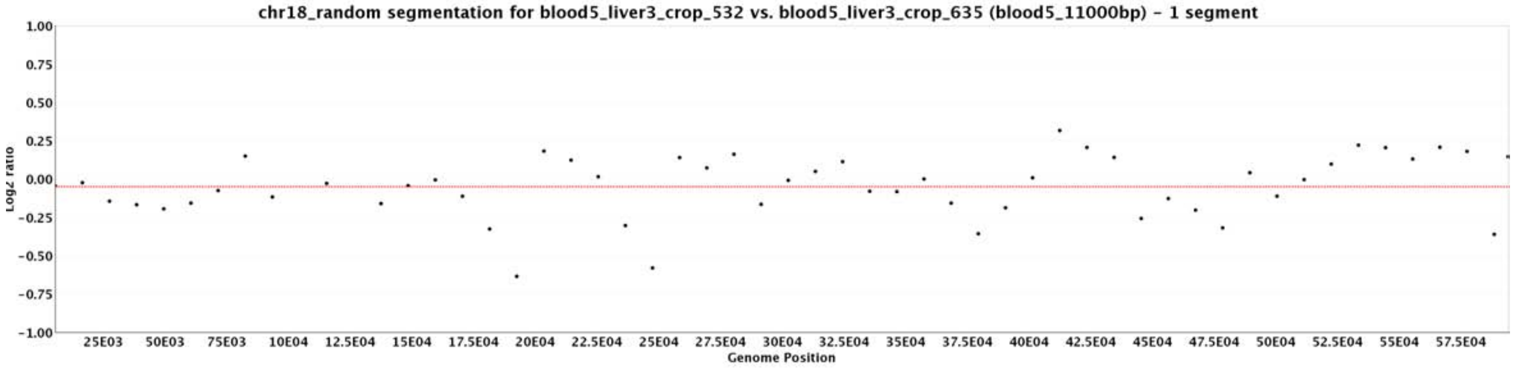
chr18_random segmentation for blood5_liver3_crop_532 vs. blood5_liver3_crop_635 (blood5_11000bp) - 1 segment
Log2 ratio
Genome Position

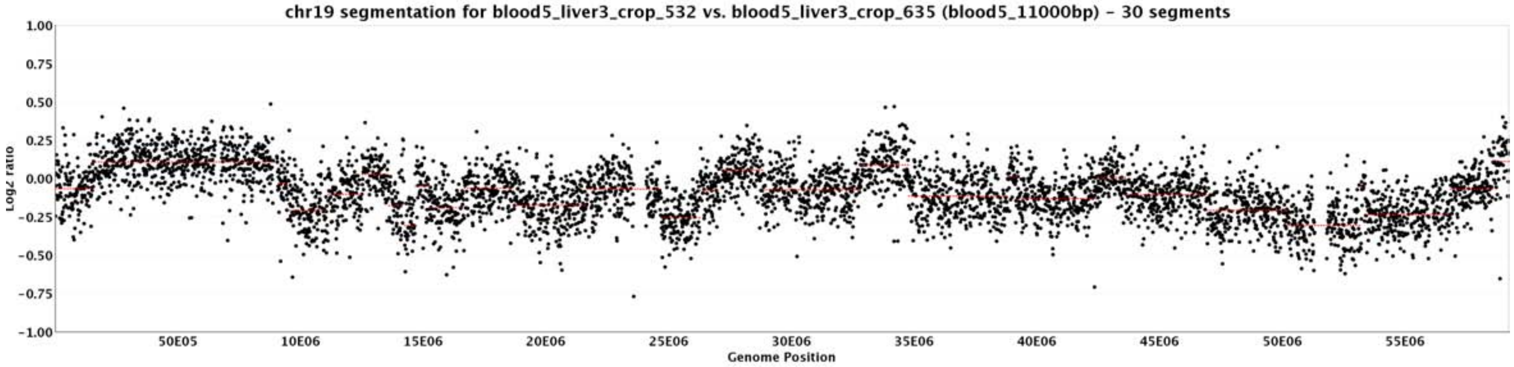
chr19 segmentation for blood5_liver3_crop_532 vs. blood5_liver3_crop_635 (blood5_11000bp) - 30 segments
Log2 ratio
Genome Position

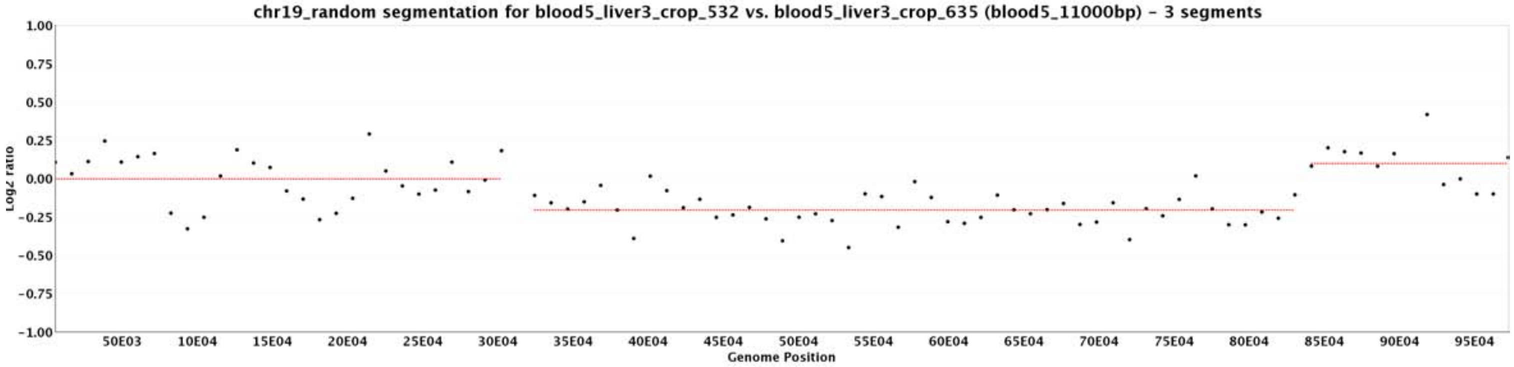
chr19_random segmentation for blood5_liver3_crop_532 vs. blood5_liver3_crop_635 (blood5_11000bp) - 3 segments
Log2 ratio
Genome Position

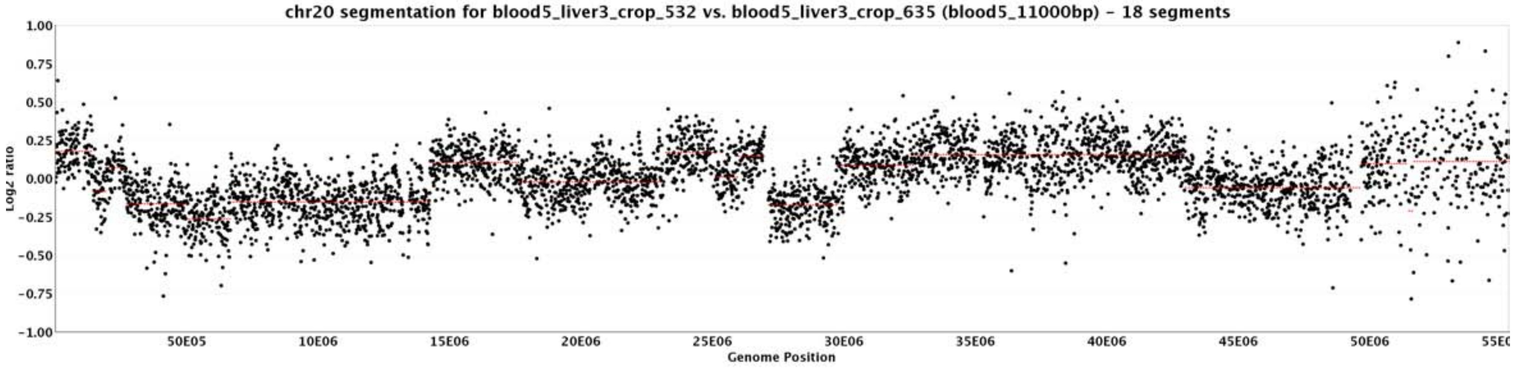
chr20 segmentation for blood5_liver3_crop_532 vs. blood5_liver3_crop_635 (blood5_11000bp) - 18 segments
Log2 ratio
Genome Position

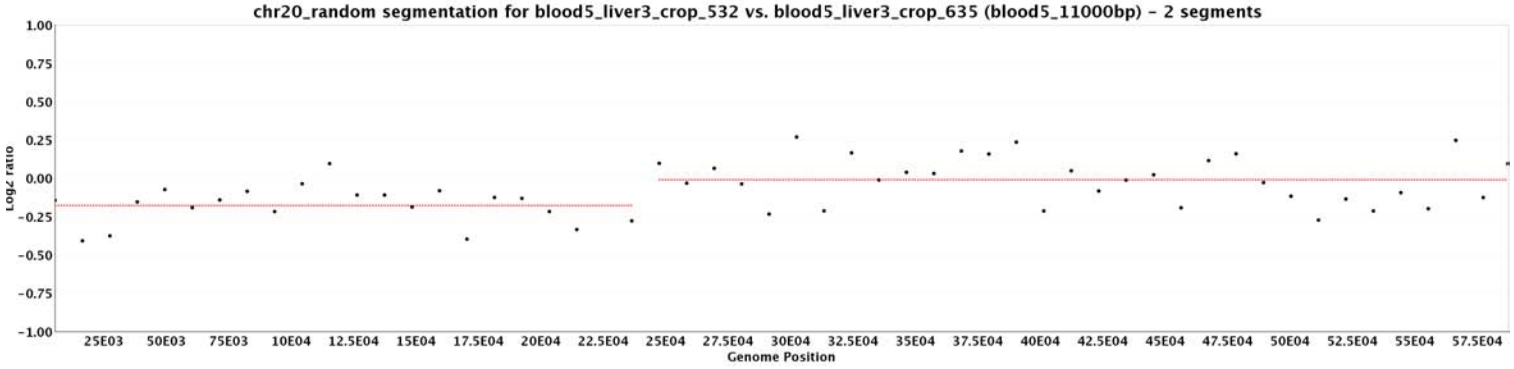
chr20_random segmentation for blood5_liver3_crop_532 vs. blood5_liver3_crop_635 (blood5_11000bp) - 2 segments
Log2 ratio
Genome Position

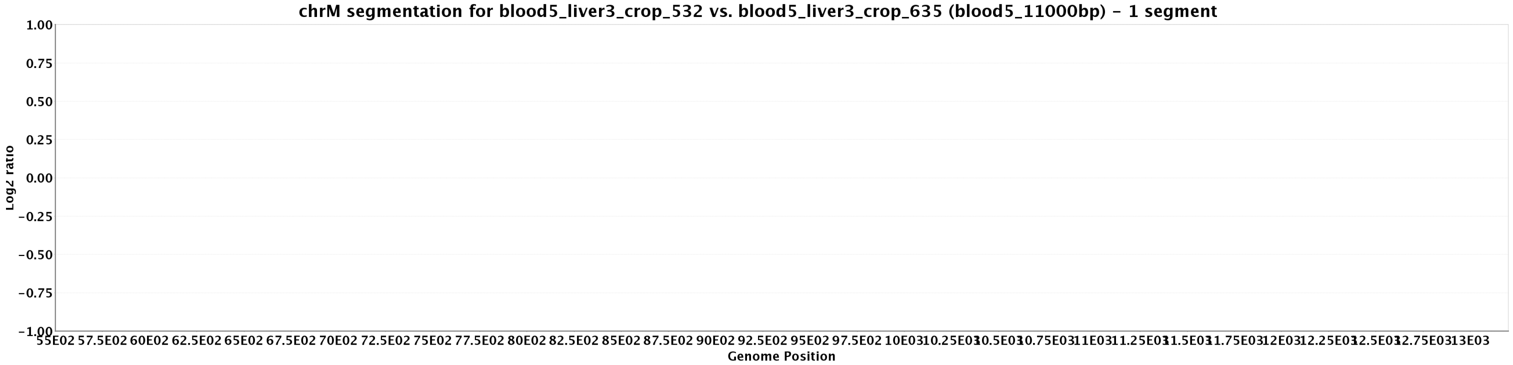
chrM segmentation for blood5_liver3_crop_532 vs. blood5_liver3_crop_635 (blood5_11000bp) - 1 segment
Log2 ratio
Genome Position

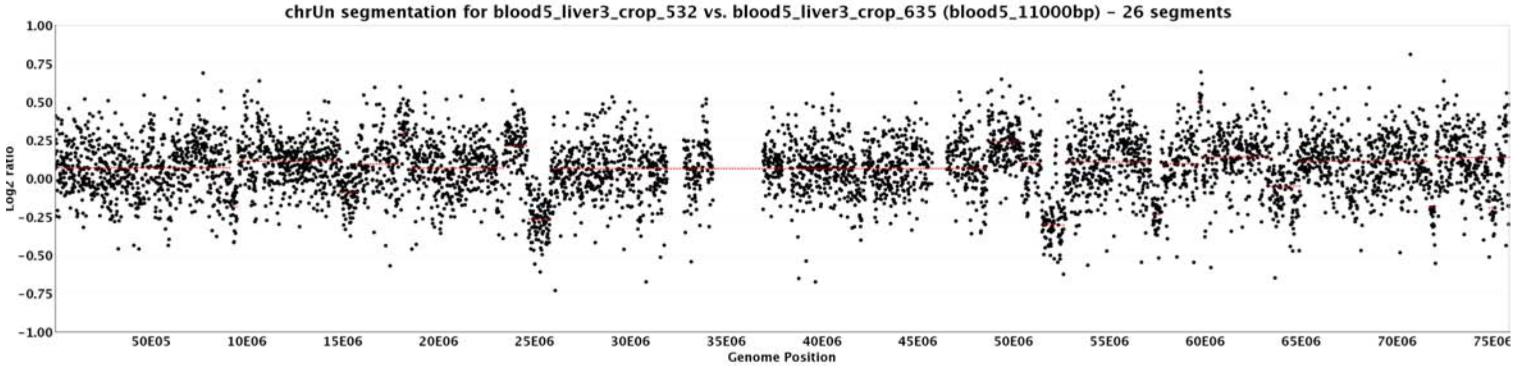
chrUn segmentation for blood5_liver3_crop_532 vs. blood5_liver3_crop_635 (blood5_11000bp) - 26 segments
Log2 ratio
Genome Position

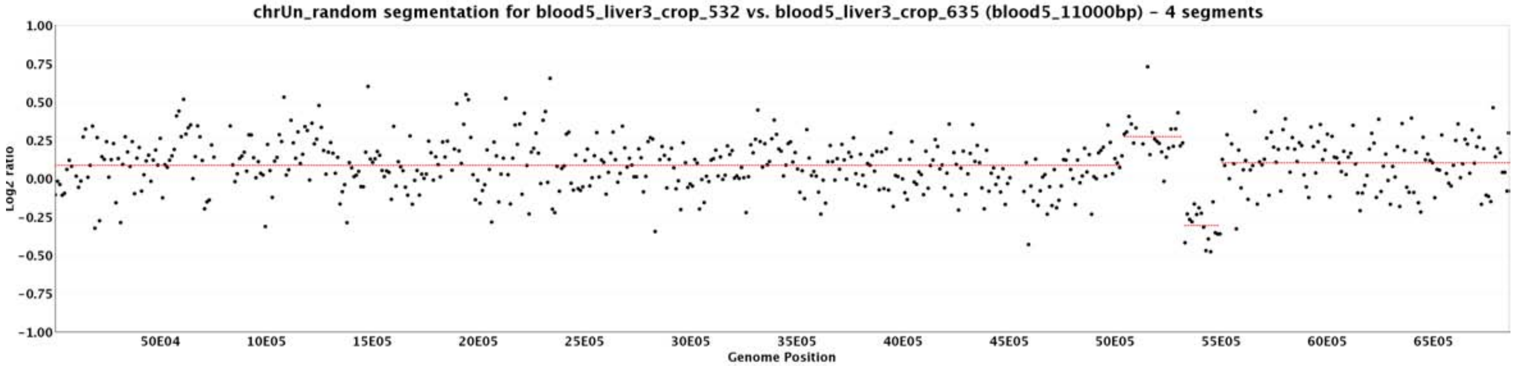
chrUn_random segmentation for blood5_liver3_crop_532 vs. blood5_liver3_crop_635 (blood5_11000bp) - 4 segments
Log2 ratio
Genome Position

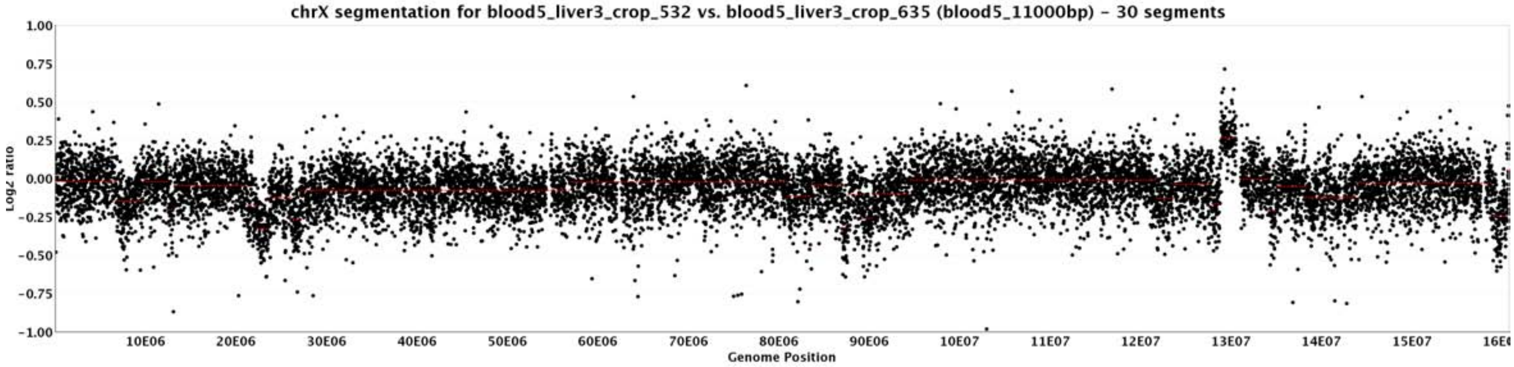
chrX segmentation for blood5_liver3_crop_532 vs. blood5_liver3_crop_635 (blood5_11000bp) - 30 segments
Log2 ratio
Genome Position

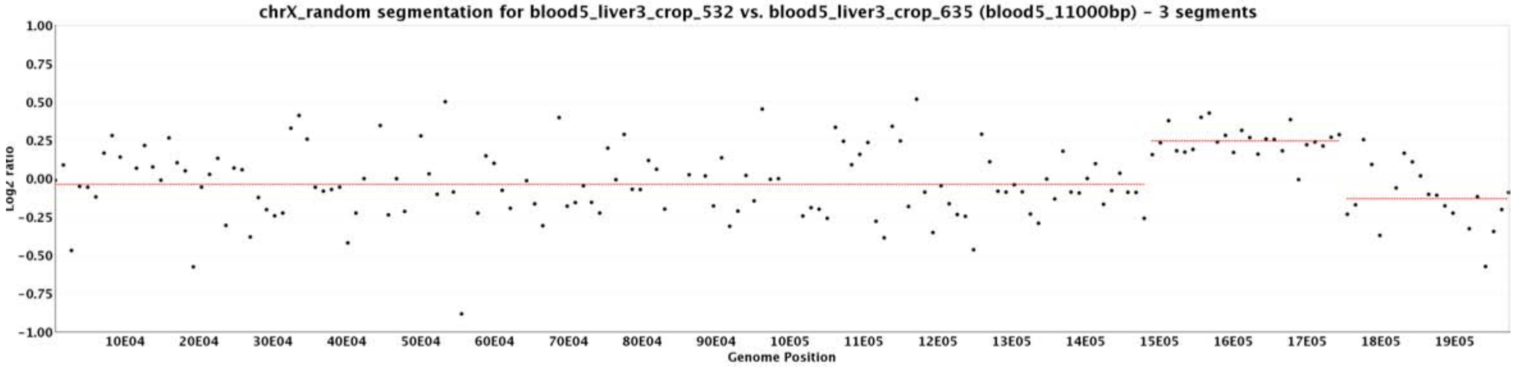
chrX_random segmentation for blood5_liver3_crop_532 vs. blood5_liver3_crop_635 (blood5_11000bp) - 3 segments
Log2 ratio
Genome Position
